# Synthesis, α-mannosidase inhibition studies and molecular modeling of 1,4-imino-ᴅ-lyxitols and their C-5-altered *N*-arylalkyl derivatives

**DOI:** 10.3762/bjoc.19.24

**Published:** 2023-03-06

**Authors:** Martin Kalník, Sergej Šesták, Juraj Kóňa, Maroš Bella, Monika Poláková

**Affiliations:** 1 Institute of Chemistry, Center for Glycomics, Slovak Academy of Sciences, Dúbravská cesta 9, 845 38 Bratislava, Slovakia; 2 Medical Vision, Civic Research Association, Záhradnícka 4837/55, 82108 Bratislava, Slovakia

**Keywords:** glycosidase inhibitor, Golgi mannosidase II, iminosugar, inhibition, molecular modeling

## Abstract

A synthesis of 1,4-imino-ᴅ-lyxitols and their *N*-arylalkyl derivatives altered at C-5 is reported. Their inhibitory activity and selectivity toward four GH38 α-mannosidases (two Golgi types: GMIIb from *Drosophila melanogaster* and AMAN-2 from *Caenorhabditis elegans*, and two lysosomal types: LManII from *Drosophila melanogaster* and JBMan from *Canavalia ensiformis*) were investigated. 6-Deoxy-DIM was found to be the most potent inhibitor of AMAN-2 (*K*_i_ = 0.19 μM), whose amino acid sequence and 3D structure of the active site are almost identical to the human α-mannosidase II (GMII). Although 6-deoxy-DIM was 3.5 times more potent toward AMAN-2 than DIM, their selectivity profiles were almost the same. *N*-Arylalkylation of 6-deoxy-DIM resulted only in a partial improvement as the selectivity was enhanced at the expense of potency. Structural and physicochemical properties of the corresponding inhibitor:enzyme complexes were analyzed by molecular modeling.

## Introduction

Iminosugars are analogs of monosaccharides in which the endocyclic oxygen atom is replaced with a nitrogen atom [1–5]. These compounds have been attracting attention due to their broad spectrum of biological activities [6]. A number of synthetic and naturally occurring iminosugars are able to inhibit various enzymes of medicinal interest including glycosidases, glycosyltransferases and many other carbohydrate processing enzymes that are involved in diseases such as viral infections, diabetes or cancer and lysosomal storage disorders [7–9]. Thus, iminosugar derivatives are promising candidates for pharmaceuticals, and many of them have already been approved for treatments, for example miglitol (type 2 diabetes), miglustat (lysosomal storage disorders, e.g., Gaucher disease) and migalstat (Fabry disease, an orphan drug) [8,10].

Natural iminosugars can be monocyclic or bicyclic compounds since the presence of the nitrogen atom allows for a formation of an additional cycle. In synthetic iminosugar analogs, various structural modifications are possible and many of these compounds inhibit glycoprocessing enzymes [11–14].

One of the best known iminosugars is the natural alkaloid (−)-swainsonine, which is a nanomolar inhibitor of human Golgi α-mannosidase II (GMII, GH38 family, E.C.3.2.1.114). Although such inhibition has been found to suppress metastasis, the potentially positive effect of swainsonine on cancer patients is strongly reduced by its severe side effects [15–16]. These are associated with the accumulation of oligomannoside structures in tissues, serum, and urine, which is caused by the co-inhibition of lysosomal α-mannosidase (LMan, GH38 family, E.C.3.2.1.24) [17] due to structural similarities between the active sites of GMII and LMan. Lysosomal α-mannosidases operate at a lower pH value (pH 4.5) compared to Golgi-type mannosidases (pH 6) and have a significantly broader substrate specificity. Another type of lysosomal α-mannosidase is Jack bean α-mannosidase from *Canavalia enciformis* (JBMan, GH38 family, E.C.3.2.1.24) which operates in the pH range 4–5. This class II mannosidase is inhibited by swainsonine very effectively (IC_50_ = 1–5 × 10^−7^ M) [18] and is frequently used as an acidic α-mannosidase model for structural and mechanistic inhibition studies [19–21]. On the other hand, *Caenorhabditis elegans* α-mannosidase II (AMAN-2) represents a Golgi-type α-mannosidase (GH38 family, E.C.3.2.1.114) and has the amino acid sequence and predicted 3D structure (based on a built homology model) of the active site almost identical to those of human GMII [22]. In addition, analysis of the available X-ray structures of GH38 enzymes such as dGMII [23], bovine lysosomal α-mannosidase II (bLMan) [17] and JBMan [24] showed that the active sites of Golgi and acidic α-mannosidases are structurally very similar. This explains why potent GMII inhibitors like swainsonine tend to lack significant selectivity. Therefore, the search for potent and selective GMII inhibitors is rather challenging.

**Figure 1:**
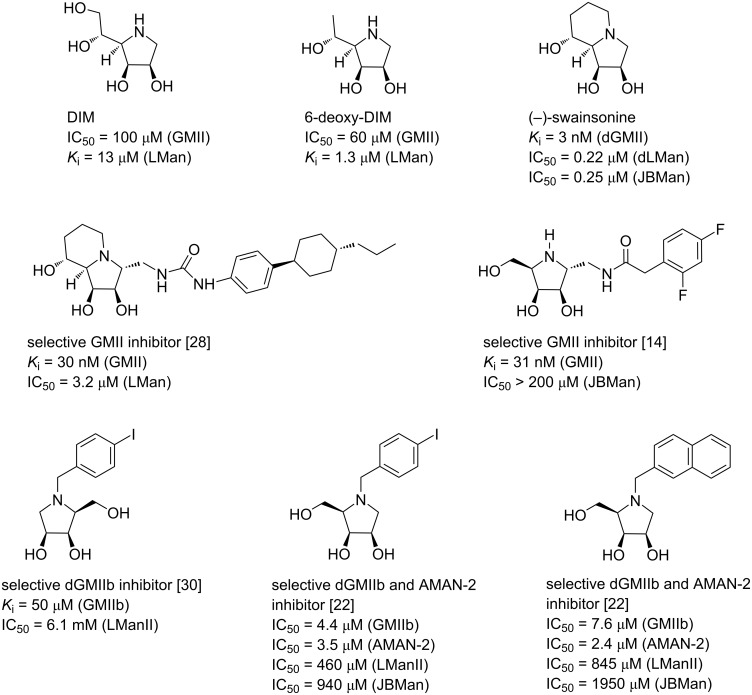
Natural iminosugars (1,4-dideoxy-1,4-imino-ᴅ-mannitol (DIM) and swainsonine) and selected examples of synthetic Golgi α-mannosidase II inhibitors with their activity profile toward the Golgi-type α-mannosidases.

Over the last decades, almost all attempts at overcoming the selectivity challenge posed by swainsonine have not been successful [25–27]. The only exception is the latest study by Cheng et al. who reported breakthrough findings in the development of highly potent and selective human GMII inhibitors. A combination of natural product-inspired combinatorial chemistry and computation-guided synthesis provided a nanomolar GMII inhibitor with a 106-fold selectivity over the human LMan while no oligomannose accumulation was observed in animal models [28] (Figure 1).

Another approach to developing selective GMII inhibitors could be based on screening derivatives of 1,4-dideoxy-1,4-imino-ᴅ-mannitol (DIM) as the configurations of all its stereogenic centers match those of swainsonine. Furthermore, DIM is a micromolar GMII inhibitor, which is easily accessible on a large scale from ᴅ-mannose by well-established methods [15,29]. In general, improvements of physicochemical and inhibitory properties of monocyclic iminosugars can be achieved by an alkylation of the endocyclic nitrogen. This reduces their high hydrophilicity which in turn may have a positive impact on the interactions with the hydrophobic pocket of the GMII active site. For example, *N*-benzylation of DIM afforded a slightly more potent GMII inhibitor than parent DIM [15]. Also, screening of a large library of *N*-alkyl and *N*-arylalkyl DIMs revealed that they are less effective inhibitors of JBMan than DIM, indicating that *N*-alkylation might lead to better selectivity profiles. However, this library has not been assayed for GMII and LM, therefore the compounds’ true inhibitory activity and selectivity toward these relevant GH38 enzymes remain unknown [29].

In this regard, another promising strategy seems to be a modification of the pyrrolidine core at the C-1 position. For example, attaching an amide moiety directly to C-1 in pyrrolidines possessing the ᴅ-*lyxo*-configuration and bearing a free endocyclic nitrogen resulted in micromolar GMII inhibitors. In addition, their potency toward GMII was found to be 2.4–3.8 times lower than toward *Drosophila melanogaster* α-mannosidase dGMII (dGMII) [26]. The incorporation of an *N*-acyl aminomethyl group onto C-1 further enhanced the potency and led to highly selective nanomolar GMII inhibitors [14] (Figure 1).

Our first investigations were focused on the development of selective GMII inhibitors derived from 1,4-imino-1,4-dideoxy-ʟ-lyxitol. Initially, we modified this core by an alkylation of the endocyclic nitrogen with a benzyl or alkyl unit functionalized either with non-polar hydrocarbons or a polar amine, amidine and guanidine group. The ensuing assay with the model GMIIb enzyme (fruit fly Golgi α*-*mannosidase II) revealed that *N*-substitution improved both potency and selectivity, achieving inhibition constants (*K*_i_) up to 4 µM and selectivity indices (SI) up to 350. However, these enhancements were found to be significant only for those *N*-substituted analogs that bore *N*-alkyl chains without an additional aryl moiety [30–32] (Figure 1).

Next, we turned our attention to modifications of 1,4-imino-ᴅ-lyxitol which configurationally better resembles swainsonine or DIM. The most promising *N*-substituents from the previous study were selected and a small library of *N*-substituted 1,4-imino-ᴅ-lyxitols was prepared [22] (Figure 1). In addition, more relevant *Caenorhabditis elegans* α-mannosidase II (AMAN-2) (GH38 family, E.C.3.2.1.114) was included in the biochemical assay as its active site is more similar to human GMII than that of GMIIb. The resulting biochemical evaluation revealed that imino-ᴅ-lyxitols with *N*-substituents possessing a polar basic functional group (amidine or guanidine) were 6–7 times less active toward AMAN-2 than GMIIb and had poorer selectivities (SI below 35). In contrast, imino-ᴅ-lyxitols bearing a non-polar *N*-arylalkyl chain (benzyl, *p*-iodobenzyl, 2-naphthylmethyl) showed slightly higher inhibitory activities toward AMAN-2 and good to excellent selectivities [22], indicating that they are more suitable candidates for the next-generation design of potent and selective GMII inhibitors.

Therefore, the current study is dedicated to designing further modifications of these analogs. This contribution deals with the synthesis of 1,4-imino-ᴅ-lyxitols altered at C-5 and substituted at the endocyclic nitrogen by the most successful arylalkyl chains (benzyl, *p*-iodobenzyl, 2-naphthylmethyl) found in our previous studies [22,30,33]. The biological activity of the novel synthesized compounds was evaluated toward the GH38 family enzymes (AMAN-2, GMIIb, fruit fly lysosomal α-mannosidase II (LManII) and JBMan). Finally, structural and physicochemical properties of inhibitor:enzyme complexes were investigated at the theoretical level using molecular docking, hybrid quantum mechanics/molecular mechanics (QM/MM) calculations and fragmented molecular orbital pair interaction energy decomposition analysis (FMO-PIEDA).

## Results and Discussion

### Chemistry

**Scheme 1:**
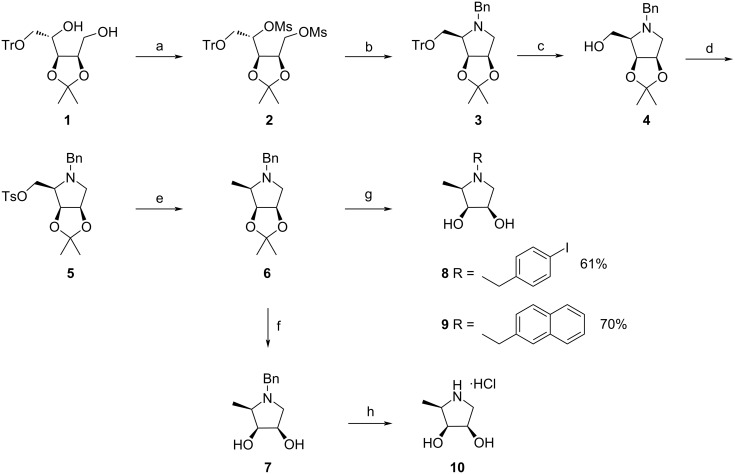
Synthesis of the key pyrrolidine **3** and the target pyrrolidines **7**–**10**. Reagents and conditions: (a) MsCl, Et_3_N, CH_2_Cl_2_, 0 °C–rt, overnight, 80%; (b) BnNH_2_, 120 °C, 7 h, quant.; (c) PTSA·H_2_O, CH_2_Cl_2_/MeOH 30:1, rt, 24 h, 69%; (d) TsCl, DMAP, CH_2_Cl_2_, 0 °C–rt, overnight, 88%; (e) LiBHEt_3_, THF, 0–40 °C, overnight, 83%; (f) 20% HCl, MeOH, rt, 72 h, 70%; (g) 1. H_2_, Pd/C, MeOH, rt, 48 h; 2. conc. HCl, 0–40 °C, 2 h, 3. ArCH_2_Br, K_2_CO_3_, DMF, 0 °C–rt, overnight.; (h) 1. H_2_, Pd/C, MeOH, rt, 2 h; 2. conc. HCl, 0–40 °C, 2 h, 68%.

The target *N*-arylalkyl 1,4-imino-ᴅ-lyxitol derivatives modified at C-5 were synthesized by the analogous approach that we had reported previously for their ʟ-enantiomers [33]. First, the key, fully protected *N*-benzylpyrrolidine **3** was prepared in two steps from known ʟ-ribitol **1** [34] in good overall yield. Next, it was converted to the C-5 deoxygenated *N*-benzylpyrrolidine **6** via trityl ether cleavage, tosylation of the deprotected OH group, and reduction of the tosylate **5**. Hydrogenolysis of the *N*-benzyl group in **6** followed by a removal of the acetonide and subsequent alkylation of the liberated amine with the corresponding arylalkyl bromides under basic conditions provided the desired *N*-arylalkyl iminosugars **8** and **9** in moderate yields. In addition, acidic hydrolysis of the acetonide group in **6** afforded the target derivative **7** which after hydrogenolysis and treatment with conc. HCl gave hydrochloride **10** (Scheme 1).

**Scheme 2:**
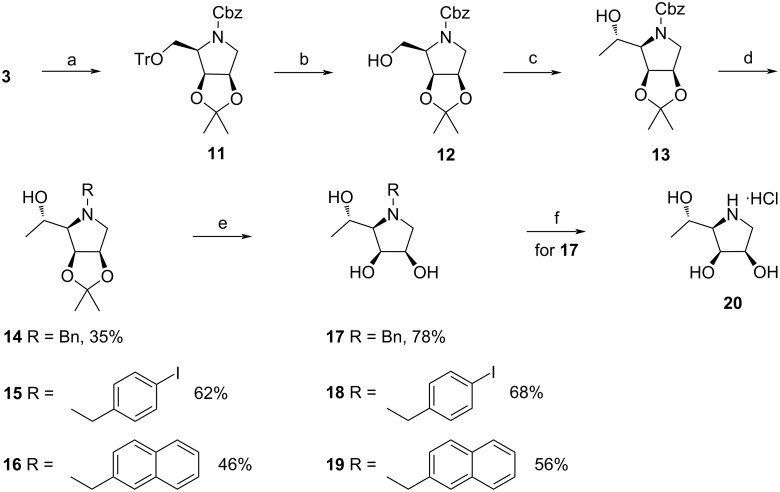
Synthesis of the intermediate **13** and the target pyrrolidines **17**–**20**. Reagents and conditions: (a) 1. H_2_, Pd/C, MeOH, rt, 48 h; 2. CbzCl, Et_3_N, CH_2_Cl_2_, 0 °C–rt, 2 h, 91% over two steps, (b) PTSA·H_2_O, CH_2_Cl_2_/MeOH 30:1, rt, 20 min, 90%; (c) 1. DMP, CH_2_Cl_2_, rt, 1 h; 2. MeMgBr, Et_2_O, 0 °C–rt, 1 h, 63% over two steps; (d) 1. H_2_, Pd/C, MeOH, rt, 2 h; 2. ArCH_2_Br, K_2_CO_3_, DMF, 0 °C–rt, overnight; (e) 20% HCl, MeOH, rt, 72 h; (f) H_2_, Pd/C, MeOH, rt, 5 h, then, conc. HCl, 0 °C, 76%.

The versatile intermediate **3** was further employed in the synthesis of the target compounds **17**–**20** (Scheme 2) homologated at the C-5 position. The transformation of pyrrolidine **3** to **13** included the replacement of the *N*-benzyl group with the Cbz group, trityl ether hydrolysis, oxidation of the liberated OH group, and stereoselective addition of MeMgBr to the resulting aldehyde functionality. Hydrogenolysis of the Cbz protecting group in **13** followed by *N*-alkylation afforded pyrrolidines **14**–**16** which after acidic hydrolysis of the isopropylidene moiety provided the desired derivatives **17**–**19**. The hydrochloride salt of the free iminosugar **20** was obtained from *N*-benzyl derivative **17** under the same reaction conditions as described for the hydrochloride **10**.

**Scheme 3:**
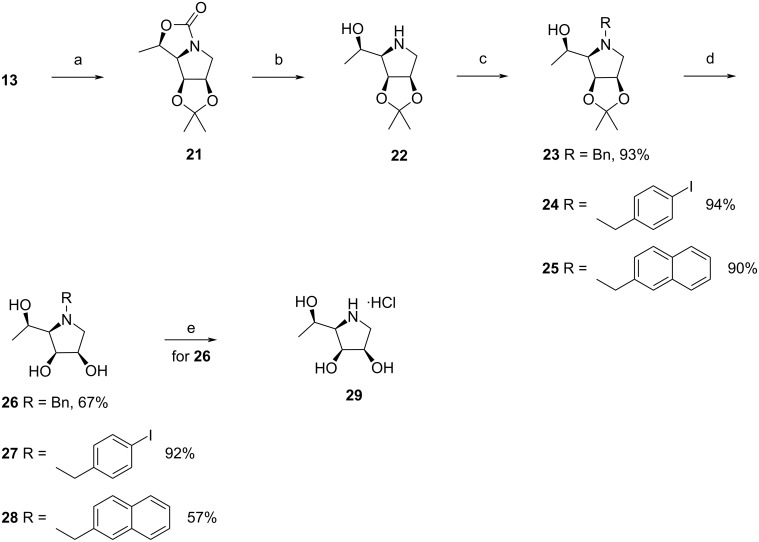
Synthesis of the target pyrrolidines **26**–**29**. Reagents and conditions: (a) Tf_2_O, pyridine, CH_2_Cl_2_, 0 °C, 1.5 h, 64%; (b) 10% aq NaOH, EtOH, reflux, 24 h, 67%; (c) ArCH_2_Br, K_2_CO_3_, DMF, 0 °C–rt, overnight; (d) 20% HCl, MeOH, rt, 72 h; (e) H_2_, Pd/C, MeOH, rt, 5 h, then, conc. HCl, 0 °C, 86%.

Next, the configuration at the C-5 stereocenter in **13** was inverted via cyclic carbamate **21**. Aminoalcohol **22** obtained after a basic hydrolysis of **21** was *N*-alkylated with the corresponding arylalkyl bromides to furnish derivatives **23**–**25**. These were subjected to an acidic hydrolysis of the acetonide moiety to give the target compounds **26**–**28**. Iminosugar **29** (6-deoxy-DIM) was obtained from *N*-benzylpyrrolidine **26** by the same procedure as described for the preparation of hydrochlorides **10** and **20** (Scheme 3). It should be noted that the hydrochlorides **20** and **29** were synthesized via derivatives **17** and **26** because the alternative approach through acetonides **13** and **22** involved tedious purifications of the final hydrochlorides.

### Enzyme assay

The potency and selectivity of the synthesized iminosugar hydrochlorides **10**, **20**, **29** and their *N*-arylalkyl analogs **7**–**9**, **17**–**19** and **26**–**28** were evaluated toward the class II GH38 α-mannosidases, and DIM and swainsonine were used as standards. The enzymes screened included two Golgi types (GMIIb and AMAN-2) and two lysosomal types (LManII and JBMan) (Table 1). As for the Golgi-type mannosidases, AMAN-2 is a more relevant enzyme because its amino acid sequence and the 3D structure of its active site are almost identical to those of human GMII [22].

**Table 1:** Inhibition (IC_50_, *K*_i_ values and selectivity index, SI) of class II GH38 α-mannosidases (GMIIb, AMAN-2, LManII and JBMan) by the synthesized iminosugar derivatives.

compound	IC_50_ [*K*_i_] (µM)				SI^a^

	GMIIb	AMAN-2	LManII	JBMan	

**7**	245 ± 35	975 ± 25	>4000	>4000	n.d.
**8**	205 ± 35	385 ± 15	>4000	>4000	n.d.
**9**	310 ± 22	605 ± 35	>4000	>4000	n.d.
**10**	525 ± 25	415 ± 15	>4000	2875 ± 275	n.d.
**17**	335 ± 37	990 ± 10	>4000	>4000	n.d.
**18**	170 ± 10	205 ± 5	>4000	>4000	n.d.
**19**	860 ± 140	1675 ± 75	>4000	>4000	n.d.
**20**	30 ± 5	48 ± 6	295 ± 20	105 ± 25	2.2^b^
**26**	33 ± 3.7 [16.2 ± 4.4 ]	115 ± 18 [58 ± 8]	505 ± 57 [270 ± 27]	298 ± 30 [80 ± 6]	1.4
**27**	13 ± 5.5 [4.2 ± 1.1]	66 ± 10 [34 ± 7]	410 ± 80 [263 ± 18]	164 ± 28 [73 ± 3]	2.1
**28**	13.5 ± 5.5 [5.2 ± 1.4]	22 ± 4 [18 ± 3]	118 ± 10 [98 ± 11]	78 ± 16 [44 ± 8]	2.4
**29**	0.12 ± 0.02 [0.065 ± 0.01]	0.24 ± 0.05 [0.19 ± 0.02]	0.82 ± 0.19 [0.38 ± 0.04]	0.32 ± 0.11 [0.12 ± 0.01]^c^	0.6
DIM	0.19 ± 0.04 [0.13 ± 0.02]	0.81 ± 0.03 [0.68 ± 0.03]	2.55 ± 0.10 [1.95 ± 0.35]	0.72 ± 0.03 [0.38 ± 0.03]	0.6
swainsonine	0.004 [0.0027]^d^	0.004 ± 0.02 0.01^e^	0.012 [0.0071]^d^	0.20^f^ [n.d.]	20
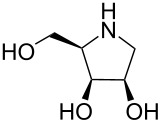 **30**	3.9 ± 0.1^g^	2.3 ± 0.1^g^	30.5 ± 3^g^	10.5 ± 1.3^g^	5^b^
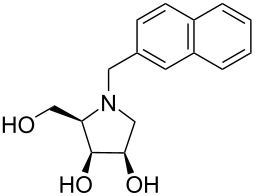 **31**	7.6 ± 1.1^g^	2.4 ± 0.1^g^	845 ± 170^g^	1950 ± 250^g^	812^b^

^a^Selectivity index, SI [*K*_i_ (JBMan)/*K*_i_ (AMAN-2)], n.d.: not determined; ^b^SI [IC_50_ (JBMan)/IC_50_ (AMAN-2)]; ^c^*K*_i_ = 0.5 µM measured by Eis [37]; ^d^IC_50_ and *K*_i_ measured by Nemčovičová et al. [35]; ^e^IC_50_ estimated from the inhibition assays measured by Paschinger et al. [36] who reported 45% inhibition of AMAN-2 by 10 nM concentration of swainsonine; ^f^IC_50_ measured by Poláková et al. [38]; ^g^IC_50_ measuared by Kóňa et al. [22].

Iminosugars **7**–**10** deoxygenated at C-5 were the least effective inhibitors toward the Golgi enzymes (IC_50_ in the range of 205–975 µM) while the lysosomal enzymes were virtually unaffected. As for the C-5 homologated imino-ᴅ-lyxitols, compounds **20** and **26**–**29** showed much higher potencies than analogues **17**–**19** toward the GH38 enzymes tested. Thus, it appears that *N*-substitution of the free iminosugar **20** considerably reduces the inhibitory activity. Among imino-ᴅ-lyxitols **26**–**29**, 6-deoxy-DIM **29** was found to be the most potent derivative (*K*_i_ = 0.065 μM and 0.19 μM for GMIIb and AMAN-2, respectively), being almost 4 times more active toward AMAN-2 than DIM. However, it also exhibited undesirably strong inhibition of LManII and JBMan, giving a low selectivity index. *N*-Arylalkylation of 6-deoxy-DIM **29** slightly enhanced the selectivity but significantly reduced the potency. Out of the *N*-arylalkylated iminosugars **26**–**28**, the naphthyl derivative **28** showed the strongest activity against AMAN-2 (*K*_i_ = 18 μM) and a selectivity similar to the *p*-iodobenzyl analog **27**. These findings suggest that both the C-5 hydroxy group and *R*-configuration at the corresponding carbon are necessary for retaining the inhibitory potential of the investigated imino-ᴅ-lyxitol derivatives.

### Molecular modeling

**Figure 2:**
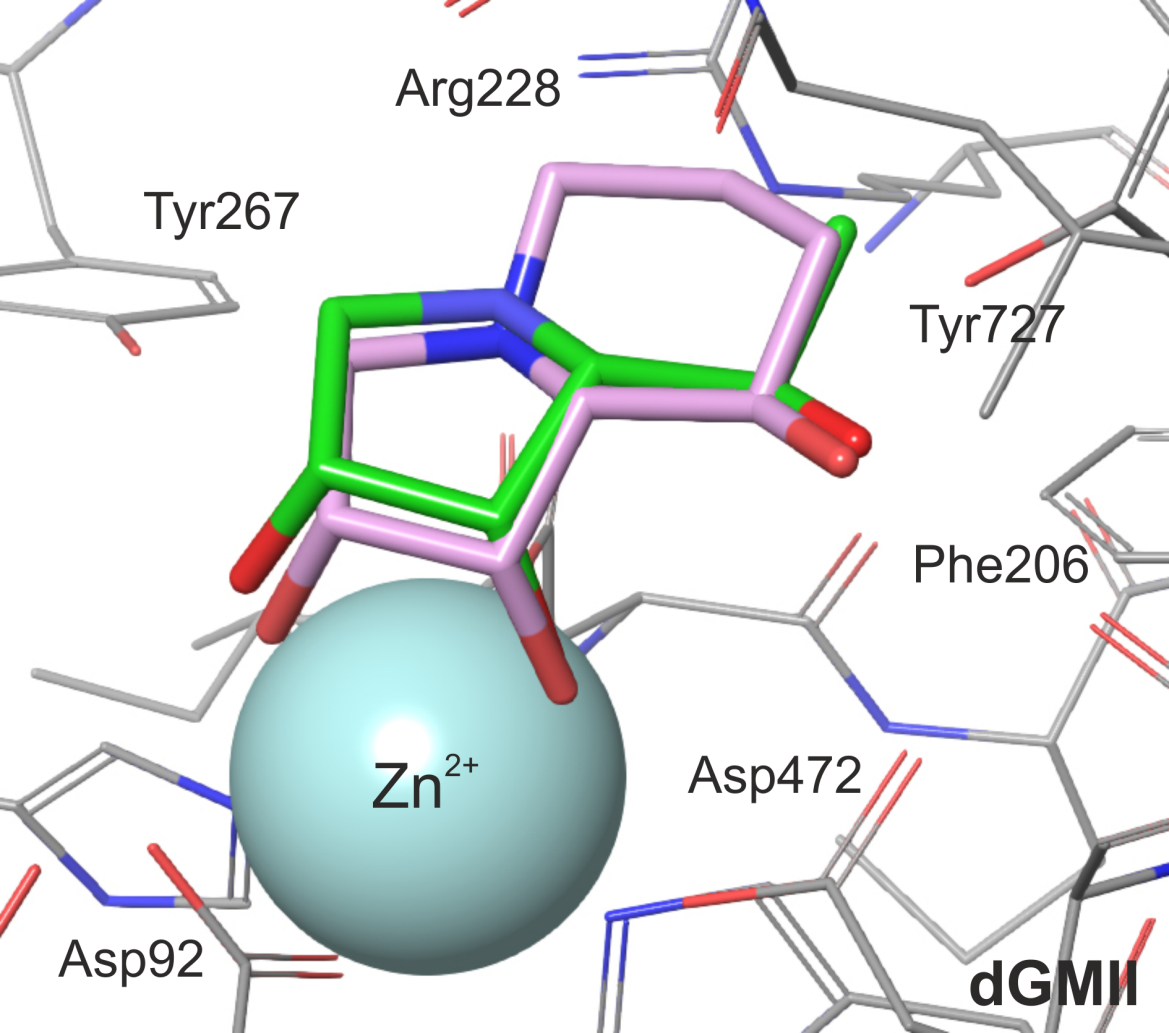
Superposition of the inhibitor **29** (green), docked into dGMII, with X-ray complexes of swainsonine (pink) with dGMII (PDB ID: 3BLB) [39]. The hydroxyethyl moiety at C-5 of **29** is placed at the same position as the hydroxypiperidine moiety of swainsonine.

In order to better understand the results of our inhibition study, the inhibitor:enzyme interactions were analyzed by molecular modeling. Structures of the inhibitors were docked into an X-ray structure of dGMII and geometries of the resulting inhibitor:dGMII complexes (for **10**, **20**, **28**–**30** and DIM) were optimized at the hybrid QM/MM level (BP86/LACVP*:OPLS2005). Based on the previous p*K*_a_ calculations [22] of DIM, **30** and **31** bound at the active site of dGMII (their p*K*_a_ = 4.9–5.4 at pH 6 of Golgi), all imino-ᴅ-lyxitol derivatives in this study were modeled in the neutral form (despite their p*K*_a_ values in aqueous solution may be higher than 7). Superimposing the binding pose of the most potent inhibitor **29** in the active site of dGMII on the bound swainsonine in the X-ray complex (PDB ID: 3BLB) [23,39] (Figure 2) showed that **29** and swainsonine bind to GMII in a similar manner. The pyrrolidine ring of **29** interacts with the Zn^2+^ ion cofactor, amino acid residues Asp92, Asp204 (catalytic nucleophile), Asp341 (catalytic acid), Asp472, and Trp 95. The (*R*)-1-hydroxyethyl group at the C-5 position of the ring forms hydrogen bonds with the side chains of Tyr727 and Asp472 [*d*(C5-OH···Tyr727-OH) = 1.48 Å, and *d*(C5-OH···Asp472-COO^−^) = 1.55 Å, BP86/LACVP*:OPLS2005] and interacts with the hydrophobic pocket created by Tyr727, Phe206 and Trp415. Also, the binding position of this side chain is in a good overlap with the hydroxyethylene part of the piperidine ring of swainsonine. This indicates that the (*R*)-1-hydroxyethyl group of **29** could mimic the interactions of the hydroxypiperidinyl moiety of swainsonine in the active site of dGMII.

**Figure 3:**
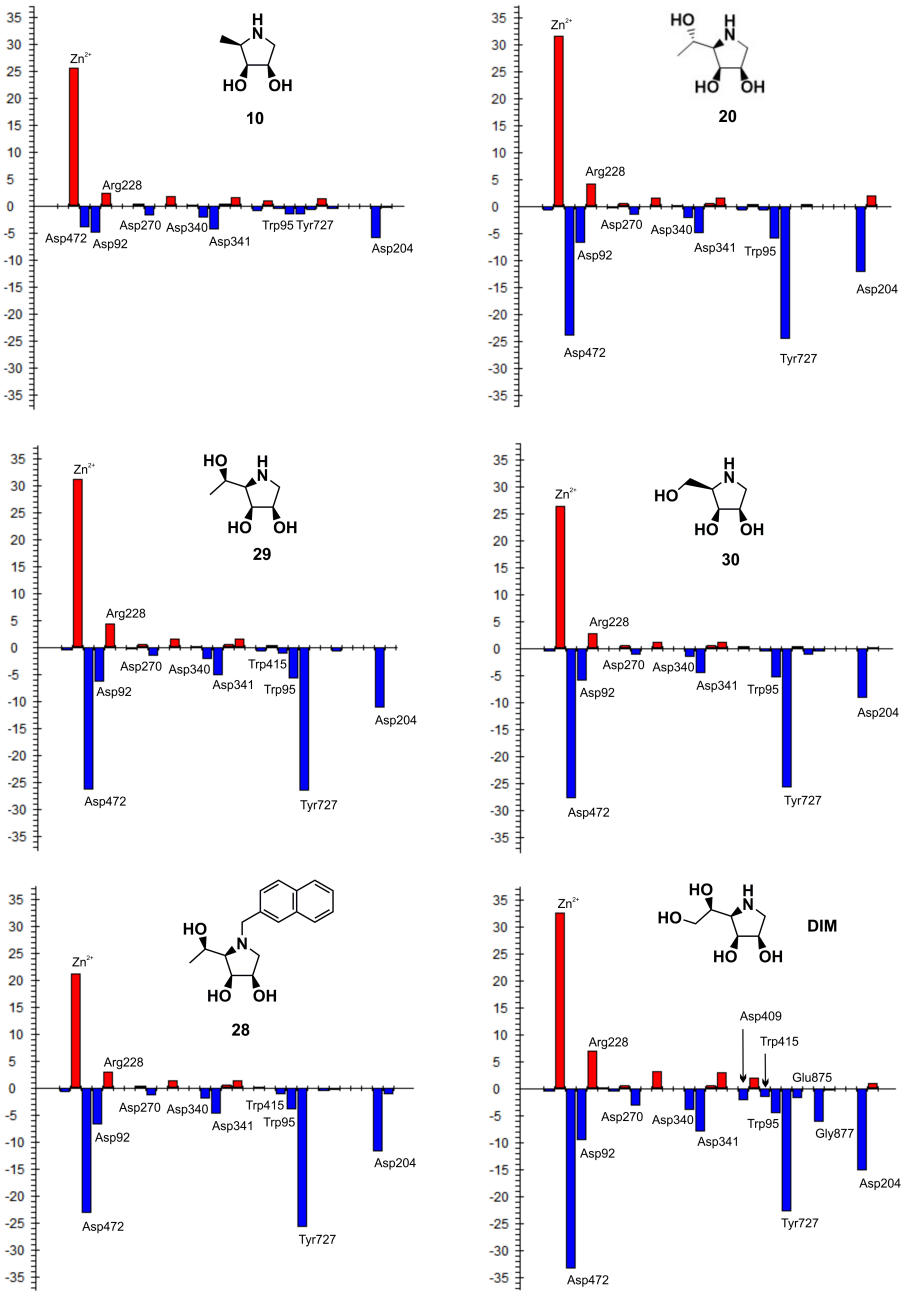
FMO-PIEDA total pair interaction energies (Δ*E*_linker-E_) (in kcal mol^−1^) between the structural moiety at C-5 of the inhibitor (**10**, **30**, **29**, **28**, **20** and DIM) and the active-site amino acid residues of dGMII. The most significant Δ*E*_linker-E_ are marked.

To evaluate the inhibitory effect of the (*R*)-1-hydroxyethyl substituent of **29** and other derivatives synthesized in this work, pair interaction energies between the bound inhibitors (**29**, **10**, **20**, **28**, **30** and DIM) and amino acid residues of dGMII were calculated at the quantum mechanics level (FMO-PIEDA-MP2/6-31G*) in an active-site model of the inhibitor:enzyme complexes optimized at the hybrid QM/MM level (BP86/LACVP*:OPLS2005). The FMO-PIEDA results are compiled in Table 2 and visualized in Figure 3. Firstly, the results for the complex **29**:dGMII were analyzed: the overall interaction energy (Δ*E*_I-E_) between **29** and the enzyme is −563.6 kcal mol^−1^, towards which the interaction energy between the (*R*)-1-hydroxyethyl group of **29** and the enzyme (Δ*E*_linker-E_ = −49.3 kcal mol^−1^) contributes only 9%. As can be seen in Figure 3, the main Δ*E*_linker-E_ contributors are Tyr727 (−26.7 kcal mol^−1^) and Asp472 (−26.3 kcal mol^−1^). Both of these amino acid residues use their side chains to interact with the hydroxy group of the (*R*)-1-hydroxyethyl moiety of **29**. The interaction with Trp95 is also significant but to a lesser extent (−5.8 kcal mol^−1^), and the overall interaction of **29** with the Phe206-Trp415 hydrophobic pocket is insignificant (less than −1.2 kcal mol^−1^). Therefore, the main contributors remain Tyr727 and Asp472 (two hydrogen bonds). A similar conclusion can also be made for the other calculated inhibitors with one (**20**, **28**, **30**) or two (DIM) hydroxy groups at C-5. This explains why the inhibitory activity of **29** [*K*_i_(AMAN-2) = 0.19 µM] is only slightly better than the previously synthesized derivative **30** (with a hydroxymethyl moiety at C-5) [*K*_i_(AMAN-2) = 2.3 µM] [22].

**Table 2:** Interaction energies (Δ*E*_I-E_, in kcal mol^−1^) for complexes (inhibitor:dGMII) calculated at the MP2//BP86 level. Also interaction energies between the enzyme and the inhibitor fragments [the pyrrolidine core (Δ*E*_ring-E_) or the structural moiety at C-5 (Δ*E*_linker-E_)] are also compiled.

	C-5 linker	*N*-substitution	ring conform.	Δ*E*_I-E_	Δ*E*_ring-E_	Δ*E*_linker-E_

**10**	-CH_3_	-H	*E*_1_/^2^*E*	−512.09	−516.22	4.13
**20**	(1*S*)-CH_2_(OH)-CH_3_	-H	^2^*E*/*E*_1_	−551.18	−508.17	−43.01
**30**	-CH_2_-OH	-H	*E*_1_	−569.30	−518.31	−50.99
**29**	(1*R*)-CH_2_(OH)-CH_3_	-H	*E*_1_	−563.60	−514.35	−49.25
**28**	(1*R*)-CH_2_(OH)-CH_3_	*N*-2-naphthylmethyl	*E*_1_	−558.78	−502.70	−56.07
DIM	-CH_2_(OH)-CH_2_-OH	-H	*E*_1_/^2^*E*	−578.74	−514.58	−64.16

**Figure 4:**
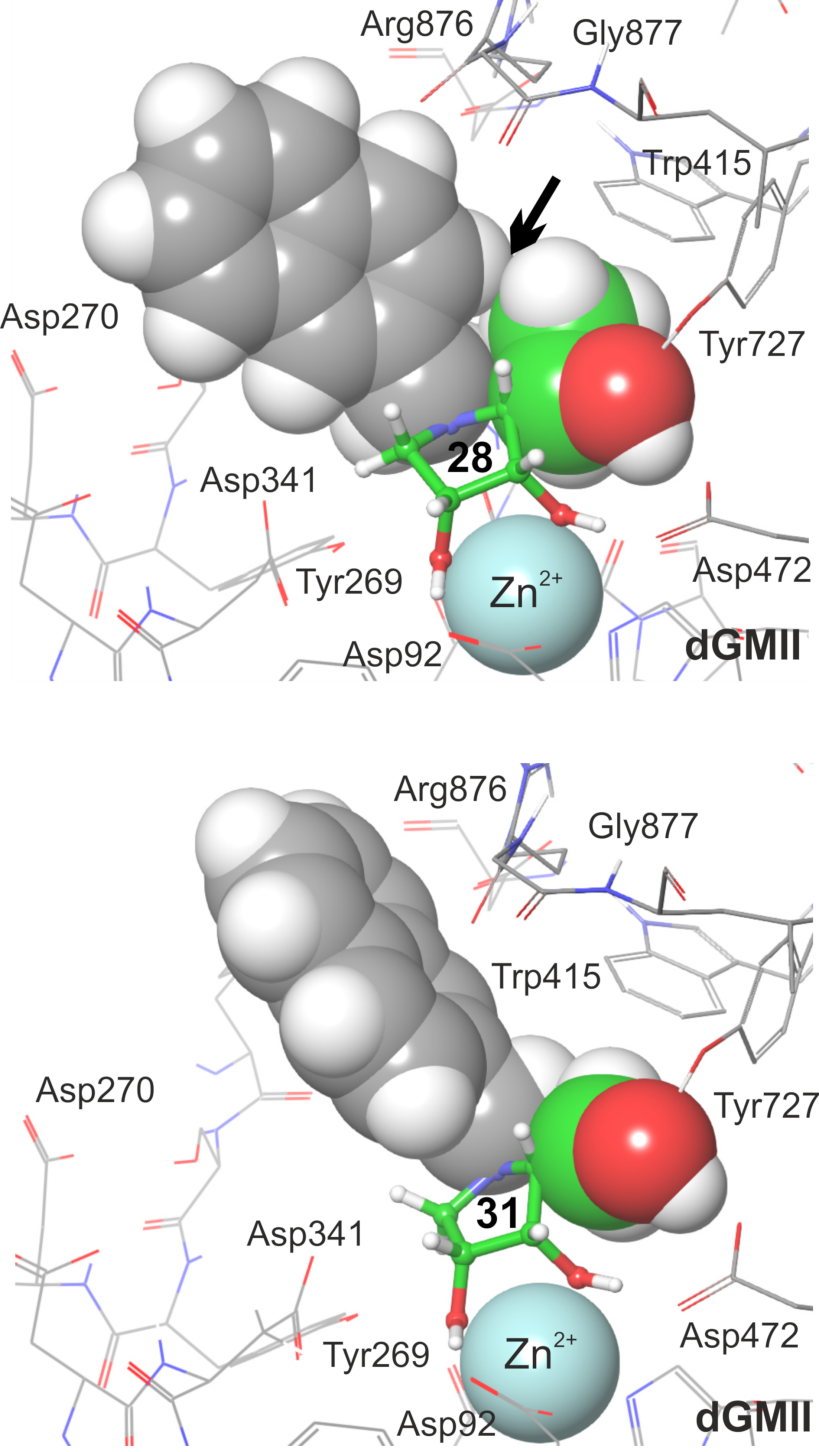
QM/MM optimized complexes **28**:dGMII (top) and **31**:dGMII (bottom) [22]. *N*-2-naphthylmethyl group (grey) and (*R*)-1-hydroxyethyl group (green) of **28** and the hydroxymethyl group (green) of **31** are visualized by van der Waals spheres to highlight the unfavorable interaction (marked with a black arrow) between the *N*-2-naphthylmethyl group and the methyl component of (*R*)-1-hydroxyethyl group at C-5 of **28**. In case of **31**, the smaller hydroxymethyl group at C-5 allows for a different binding conformation of the *N*-2-naphthylmethyl group.

**Figure 5:**
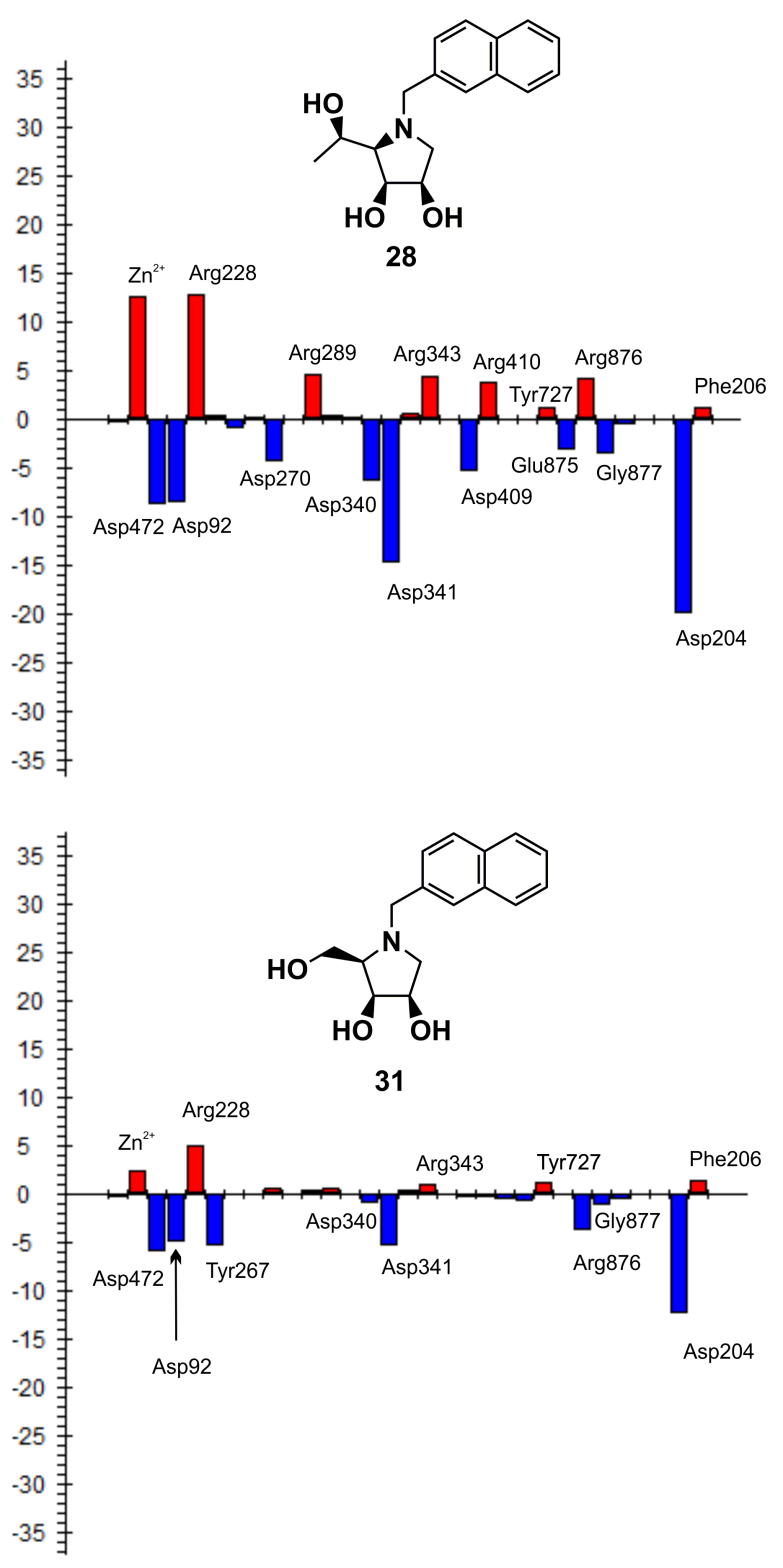
FMO-PIEDA total pair interaction energies (Δ*E*_linker-E_) (in kcal mol^−1^) between the *N*-2-naphthylmethyl moiety of the inhibitors **28** and **31** and the active-site amino acid residues of dGMII. The most significant Δ*E*_linker-E_ are marked.

To evaluate the inhibitory effect of the structural moiety at C-5 of the 1,4-imino-ᴅ-lyxitols, FMO-PIEDA interaction energies were calculated for another five derivatives (**10**, **20**, **30**, **28** and DIM). The inhibitory activities of these derivatives increased in the following order: **10** [IC_50_(AMAN-2) = 415 µM] < **20** [IC_50_(AMAN-2) = 48 µM] < **28** [IC_50_(AMAN-2) = 22 µM] < **30** [IC_50_(AMAN-2) = 2.3 µM] < DIM [IC_50_(AMAN-2) = 0.81 µM] < **29** [IC_50_(AMAN-2) = 0.24 µM]. Interestingly, almost the same trend was found for the overall FMO-PIEDA interaction energies (Δ*E*_I-E_) and the energies (Δ*E*_linker-E_) between the side chain at C-5 and the active site (Table 2). Δ*E*_I-E_ and Δ*E*_linker-E_ increase in the following order: **10** (−512.09 kcal mol^−1^) < **20** (−551.18 kcal mol^−1^) < **28** (−558.78 kcal mol^−1^) < **29** (−563.60 kcal mol^−1^) < **30** (−569.30 kcal mol^−1^) < DIM (−578.74 kcal mol^−1^); and **10** (+4.13 kcal mol^−1^) < **20** (−43.01 kcal mol^−1^) < **29** (−49.25 kcal mol^−1^) < **30** (−50.99 kcal mol^−1^) < **28** (−56.07 kcal mol^−1^) < DIM (−64.16 kcal mol^−1^), respectively. The calculations predict that the functional group attached to C-5 of the inhibitor must bear one or two hydroxy groups (with *R*-configuration in case of **28**, **29** and DIM). The methyl group itself (in **10**) contributes repulsively and decreases the overall interaction energy. Thus, only hydroxymethyl, (*R*)-1-hydroxyethyl and (*R*)-1,2-dihydroxyethyl are suitable substituents at the C-5 position of 1,4-imino-ᴅ-lyxitols. The calculations further predict that alkylation of the nitrogen atom may further increase the interactions of the (*R*)-1-hydroxyethyl group at C-5 with the enzyme (*N*-2-naphthylmethyl in **28**). However, this alkylation also decreases the interactions of the lyxitol core with the enzyme (Δ*E*_ring-E_ = −502.70 kcal mol^−1^ of **28** is weaker than Δ*E*_ring-E_ = −514.35 kcal mol^−1^ of **29**) and the overall Δ*E*_I-E_ for **28** became lower than for **29**, which is in agreement with the measured inhibitory activities of these compounds. This is a surprising result because *N*-alkylation of **30** (structure **31** in Table 1) did not decrease inhibition of Golgi-type α-mannosidases [22]. It seems that the slightly bulkier (*R*)-1-hydroxyethyl group at C-5 of **29** compared to the hydroxymethyl at C-5 of **30** allows for a different conformation of the *N*-2-naphthylmethyl group in the active site of α-mannosidases (Figure 4). This would induce a shift in the binding pose of the lyxitol core in **28** compared to **29** and a subsequent weakening in Δ*E*_ring-E_ (from −514.35 kcal mol^−1^ in **29** to −502.70 kcal mol^−1^ in **28**), which is the major component of the overall interaction energy between the inhibitor and the enzyme. This assumption was further supported by additional FMO-PIEDA calculations for **31**. In both **31** and **28**, the 2-naphthylmethyl group is attached to the ring nitrogen of the inhibitor. The interaction energy between the *N*-2-naphthylmethyl moiety of the inhibitors and the enzyme was almost the same (−30.67 kcal mol^−1^ for **31** and −30.99 kcal mol^−1^ for **28**, Figure 5), confirming that this substituent is suitable for interacting with the enzyme, but at the same time it worsened the interactions of the lyxitol core of **28** (in connection to the mentioned (*R*)-1-hydroxyethyl group at C-5).

## Conclusion

1,4-Imino-ᴅ-lyxitols and their C-5 altered *N*-arylalkyl derivatives were synthesized for α-mannosidase inhibition studies. Their evaluation revealed that deoxygenation at C-5 (derivatives **7**–**10**) provided the least effective inhibitors of the target Golgi enzymes. The comparison between iminosugars **17**–**20** and their C-5 epimers **26**–**29** showed that the hydroxy group must adopt an *R*-configuration in order to exhibit a strong inhibition profile. Also, unsubstituted 6-deoxy-DIM **29** turned out to be the best inhibitor out of all the analogues synthesized in this study as its *N*-arylalkylation significantly reduced potency and barely improved selectivity. This is in sharp contrast to the previously synthesized *N*-2-naphthylmethyl 1,4-imino-ᴅ-lyxitol which showed a high selectivity toward Golgi-type α-mannosidases [22]. The FMO-PIEDA calculations revealed that *N*-2-naphthylmethyl is too bulky for the 6-deoxy-DIM core and causes weaker binding of the inhibitor ring to the enzyme. Therefore, the next efforts should be focused on the identification of a novel *N*-substitution pattern of the DIM skeleton that would have more beneficial effects on the inhibition profile.

## Supporting Information

File 1 Experimental (synthesis, enzyme assay, molecular modelling).

File 2 Copies of NMR spectra.

File 3 Optimized QM/MM complexes (inhibitor:enzyme).
