# Supplementary material for: Synthesis, α-mannosidase inhibition studies and molecular modeling of 1,4-imino-ᴅ-lyxitols and their C-5-altered N-arylalkyl derivatives
Source: Beilstein J Org Chem. 2023 Mar 6;19:282–93. doi: 10.3762/bjoc.19.24 (PMC10012049; doi:10.3762/bjoc.19.24)
Supplement: File 1 — Experimental (synthesis, enzyme assay, molecular modelling). [file Beilstein_J_Org_Chem-19-282-s001.pdf]

BEILSTEIN JOURNAL OF ORGANIC CHEMISTRY

Supporting Information

for

Synthesis, α-mannosidase inhibition studies and molecular modeling of 1,4-imino-D-lyxitols and their C-5-altered *N*-arylalkyl derivatives

Martin Kalník, Sergej Šesták, Juraj Kóňa, Maroš Bella and Monika Poláková

Beilstein J. Org. Chem. **2023,** *19,* 282–293. doi:10.3762/bjoc.19.24

Experimental (synthesis, enzyme assay, molecular modelling)

License and Terms: This is a supporting information file under the terms of the Creative Commons Attribution License (https://creativecommons.org/licenses/by/4.0). Please note that the reuse, redistribution and reproduction in particular requires that the author(s) and source are credited and that individual graphics may be subject to special legal provisions.
The license is subject to the *Beilstein Journal of Organic Chemistry* terms and conditions: (https://www.beilstein-journals.org/bjoc/terms)

Table of contents

Experimental S1
General S1
Synthesis S2
Enzyme assay S14
Molecular modeling S15
References S17

Experimental

General

TLC was performed on aluminum sheets precoated with silica gel 60 F254 (Merck). Visualization was achieved by immersing the plates into a 10% solution of phosphomolybdic acid (PMA) in ethanol followed by heating the plate. Flash column chromatography was carried out on silica gel 60 (0.040–0.060 mm, Merck) with distilled solvents. All commercially available reagents and anhydrous solvents were used as received. *p*-Nitrophenyl α-D-mannopyranoside (*p*NP-Man*p*) and Jack bean α-mannosidase were purchased from Sigma; swainsonine and DIM from Carbosynth. All reactions containing sensitive reagents were carried out under a nitrogen atmosphere. ^{1}H NMR and ^{13}C NMR spectra were recorded at 25 °C with a Bruker AVANCE III HD 400 spectrometer. Chemical shifts are given in ppm (δ) relative to the residual signal of the appropriate deuterated solvent used ($CDCl_3$, CD_3OD, D_2O). Optical rotations were determined on a Jasco P-2000 polarimeter at 20 °C. High-resolution mass spectra were recorded with an Orbitrap Elite (Thermo Scientific) mass spectrometer with ESI ionization in positive mode. The compounds for biological assays were lyophilized before the use.

Synthesis

2,3-*O*-Isopropylidene-1,4-di-*O*-methanesulfonyl-5-*O*-trityl-L-ribitol (2)

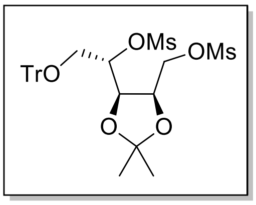

Et_3N (12.16 mL, 87.3 mmol) was added to a stirred solution of diol **1** [1] (15.16 g, 34.9 mmol) in CH_2Cl_2 (400 mL) and the reaction mixture was cooled to 0 °C (ice–water bath). MsCl (6.48 mL, 83.8 mmol) was added dropwise and the reaction mixture was stirred for 15 min. Then, the ice–water bath was removed and the reaction mixture was stirred at rt overnight. Next, the reaction mixture was washed with water (3 × 200 mL), and the aqueous layer was extracted with CH_2Cl_2 (200 mL). The combined organic extracts were dried over Na_2SO_4, filtered, and the solvent was evaporated under reduced pressure. The crude product was purified by column chromatography on silica gel (EtOAc/hexane 1:2) to afford dimesylate **2** as a white solid (16.56 g, 80%). R_F = 0.18 (EtOAc/hexane 1:2); $[\alpha]_D$ = +18.4 (*c* = 1.0; $CHCl_3$); ^{1}H NMR: (400 MHz; $CDCl_3$), δ/ppm: 7.47–7.40 (m, 6H, Ar), 7.36–7.23 (m, 6H, Ar), 7.21–7.16 (m, 3H, Ar), 4.91 (ddd, 1H, *J* = 7.2, 4.9, 2.6 Hz, 4H), 4.58–4.42 (m, 3H, H-1a, H-2, H-3), 4.33 (dt, 1H, *J* = 10.6, 5.4 Hz, H-1b), 3.57 (dd, 1H, *J* = 11.3, 2.6 Hz, H-5a), 3.46 (dd, 1H, *J* =11.3, 4.9 Hz, H-5b), 3.03 and 2.89 (2s, each 3H, 2×SO_2CH_3), 1.43 and 1.37 [2s, each 3H, $C(CH_3)_2$]; ^{13}C NMR: (100 MHz; $CDCl_3$), δ/ppm: 143.0, 128.7, 128.04, 127.4 (Ar), 109.7 [*C*$(CH_3)_2$], 87.5 (CPh_3), 78.7 (C-4), 75.1 (C-3), 74.4 (C-2), 68.2 (C-1), 62.8 (C-5), 39.3 and 37.5 ($OSOCH_3$), 27.5 and 25.4 [C(*C*$H_3)_2$]. HRMS (ESI, m/z): calculated for $C_{29}H_{34}O_9S_2$ [*M*+H]$^+$: 591.1717; found: 591.1715.

(3a*S*,4*R*,6a*R*)-5-Benzyl-2,2-dimethyl-4-((trityloxy)methyl)tetrahydro-3a*H*-[1,3]dioxolo[4,5-*c*]pyrrole (3)

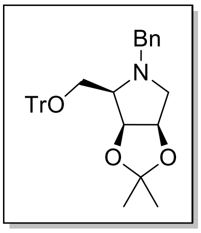

A solution of dimesylate **2** (16.43 g, 28.0 mmol) in $BnNH_2$ (100 mL) was stirred at 120 °C for 7 h. Next, $BnNH_2$ was evaporated under reduced pressure and the residue was dissolved in $CHCl_3$ (250 mL). The reaction mixture was washed with water (3 × 100 mL), the organic layer was separated, dried over Na_2SO_4, filtered and the solvent was evaporated under reduced pressure. The crude product was purified by column chromatography on silica gel (EtOAc/hexane 9:1) to afford pyrrolidine **3** as a thick yellow oil (14.33 g, quant.). R_F = 0.17 (EtOAc/hexane 9:1); $[\alpha]_D$ = −31.1 (c = 1.0; $CHCl_3$); ^{1}H NMR: (400 MHz; $CDCl_3$), δ/ppm: 7.51–7.43 (m, 5H, Ar), 7.32–7.10 (m, 15H, Ar), 4.70 (dd, 1H, *J* = 6.4, 4.9 Hz, H-3a), 4.55 (dd, 1H, *J* = 6.4, 4.6 Hz, H-6a), 4.06 (d, 1H, *J* = 13.7 Hz, NC*H*$_2$Ar), 3.65 (dd, 1H, *J* = 9.5, 6.1 Hz, C*H*$_2$OTr), 3.31 (dd, 1H, *J* = 9.5, 5.4 Hz, C*H*$_2$OTr), 3.09 (d, 1H, *J* = 13.7 Hz, NC*H*$_2$Ar), 2.95 (d, 1H, *J* = 11.1 Hz, H-6), 2.39 (q, 1H, *J* = 5.5 Hz, H-4), 1.98 (dd, 1H, *J* = 11.1, 4.7 Hz, H-6'), 1.38 and 1.29 [2s, each 3H, C(C*H*$_3)_2$]; ^{13}C NMR: (100 MHz; $CDCl_3$), δ/ppm: 144.2, 138.7, 128.9, 128.6, 128.1, 127.7, 126.8, 126.7 (Ar), 111.2 [*C*$(CH_3)_2$], 87.0 (*C*Ph_3), 81.2 (C-3a), 78.2 (C-6a), 67.5 (C-4), 62.3 (*C*H_2OTr), 59.6 (C-6), 57.8 (N*C*H_2Ar), 26.3 and 26.0 [C(*C*$H_3)_2$]. HRMS (ESI, m/z): calculated for $C_{34}H_{35}NO_3$ [*M*+H]$^+$: 506.2690; found: 506.2693.

((3a*S*,4*R*,6a*R*)-5-Benzyl-2,2-dimethyltetrahydro-3a*H*-[1,3]dioxolo[4,5-*c*]pyrrol-4-yl)methanol (4)

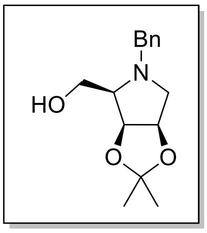

In a manner similar to [2], PTSA·H_2O (1.41 g, 7.42 mmol) was added to a stirred solution of tritylether **3** (2.50 g, 4.94 mmol) in CH_2Cl_2 (50 mL) and MeOH (25 mL) and the reaction mixture was stirred at rt for 24 h. Next, PTSA was neutralized with conc. NH_3 solution (2 mL) and the solvent was evaporated under reduced pressure. The crude product was purified by column chromatography on silica gel (EtOAc/hexane 2:3) to afford alcohol **4** as a thick yellow oil (0.90 g, 69%). R_F = 0.20 (EtOAc/hexane 2:3); $[\alpha]_D$ = −60.9 (c = 1.0; $CHCl_3$); ^{1}H NMR: (400 MHz; $CDCl_3$), δ/ppm: 7.32–7.20 (m, 5H, Ar), 4.72 (dd, 1H, *J* = 6.4, 4.9 Hz, H-3a), 4.59 (dd, 1H, *J* = 6.4, 4.7 Hz, H-6a), 4.05 (d, 1H, *J* = 13.4 Hz, NC*H*$_2$Ar), 3.97–3.93 (m, 2H, C*H*$_2$OTr), 3.23 (d, 1H, *J* = 13.4 Hz, NC*H*$_2$Ar), 3.08 (d, 1H, *J* = 11.1 Hz, H-6), 2.60 (br s, 1H, OH), 2.37 (dd, 1H, *J* = 9.2, 4.8 Hz, H-4), 2.14 (dd, 1H, *J* = 11.1, 4.7 Hz, H-6'), 1.55 and 1.32 [2s, each 2H, C(C*H*$_3$)$_2$]; ^{13}C NMR: (100 MHz; $CDCl_3$), δ/ppm: 138.0, 128.8, 128.3, 127.1 (Ar), 111.5 [*C*(CH$_3$)$_2$], 81.9 (C-3a), 77.9 (C-6a), 67.1 (C-4), 59.7 (*C*H$_2$OTr), 58.7 (C-6), 56.7 (N*C*H$_2$Ar), 26.2 and 25.0 [C(*C*H$_3$)$_2$]; HRMS (ESI, m/z): calculated for $C_{15}H_{21}NO_3$ $[M+Na]^+$: 286.1414; found 286.1422.

((3a*S*,4*R*,6a*R*)-5-Benzyl-2,2-dimethyltetrahydro-3a*H*-[1,3]dioxolo[4,5-*c*]pyrrol-4-yl)methyl 4-methylbenzenesulfonate (5)

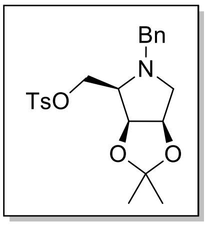

In a manner similar to [2], DMAP (723 mg, 5.92 mmol) was added to a stirred solution of alcohol **4** (780 mg, 2.96 mmol) in CH_2Cl_2 (25 mL) and the reaction mixture was cooled to 0 °C (ice–water bath). TsCl (791 mg, 4.15 mmol) was added and the reaction mixture was stirred for 15 min. Then, the ice–water bath was removed and the reaction mixture was stirred at rt for 3 h. Next, the reaction mixture was washed with water (2 × 15 mL), organic layer was separated, dried over Na_2SO_4, filtered and the solvent was evaporated under reduced pressure. The crude product was purified by column chromatography on silica gel (EtOAc/hexanes 1:4) to afford tosylate **5** as a thick yellow oil (1.1 g, 88%). R_F = 0.27 (EtOAc/hexane 1:4); $[\alpha]_D$ = −65.6 (*c* = 1.0; $CHCl_3$); ^{1}H NMR: (400 MHz; $CDCl_3$), δ/ppm: 7.79–7.75 (m, 2H, Ar), 7.35–7.12 (m, 7H, Ar), 4.61 (dd, 1H, *J* = 6.3, 4.9 Hz, H-3a), 4.55 (dd, 1H, *J* = 6.4, 4.5 Hz, H-6a), 4.31 (dd, 1H, *J* = 10.0, 6.5 Hz, C*H*$_2$OTs), 4.16 (dd, 1H, *J* = 10.0, 5.1 Hz, C*H*$_2$OTs), 3.96 (d, 1H, *J* = 13.7 Hz, NC*H*$_2$Ar), 3.25 (d, 1H, *J* = 13.7 Hz, NC*H*$_2$Ar), 3.01 (d, 1H, *J* = 11.2 Hz, H-6), 2.60 (dd, 1H, *J* = 10.9, 5.3 Hz, H-4), 2.44 (s, 3H, C*H*$_3$-C$_6$H$_4$SO$_2$), 2.09 (dd, 1H, *J* = 11.3, 4.3 Hz, H-6'), 1.37 and 1.24 [2s, each 3H, C(C*H*$_3$)$_2$]; ^{13}C NMR: (100 MHz; $CDCl_3$), δ/ppm: 144.7, 138.0, 132.8, 129.8, 128.5, 128.3, 128.1, 127.1 (Ar), 111.5 [*C*(CH$_3$)$_2$], 80.3 (C-3a), 77.9 (C-6a), 68.9 (*C*H$_2$OTs), 65.8 (C-4), 59.4 (C-6), 57.6 (N*C*H$_2$Ar), 26.1 and 25.4 [C(*C*H$_3$)$_2$], 21.63 (*C*H$_3$-C$_6$H$_4$SO$_2$); HRMS (ESI, m/z): calculated for $C_{22}H_{27}NO_5S$ $[M+H]^+$: 418.1683; found 418.1699.

(3a*S*,4*R*,6a*R*)-5-Benzyl-2,2,4-trimethyltetrahydro-3a*H*-[1,3]dioxolo[4,5-*c*]pyrrole (6)

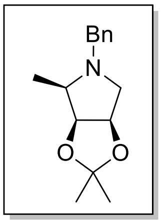

In a manner similar to [2], LiBHEt$_3$ (1.7 M in THF, 9.10 mL, 15.50 mmol) was added dropwise to a stirred solution of tosylate **5** (1.08 g, 2.59 mmol) in anhydrous THF (30 mL) at 0 °C (ice–water bath) under a nitrogen atmosphere. After 15 min of stirring, the ice–water bath was removed and the reaction mixture was stirred at 40 °C overnight. Next, the reaction mixture was carefully quenched with water (2.5 mL) while cooling to 0 °C (ice–water bath). The solvent was evaporated under reduced pressure and the residue was partitioned between water (40 mL)

and EtOAc (40 mL). Layers were separated and the aqueous layer was extracted with EtOAc (40 mL). The combined organic extracts were dried over Na_2SO_4, filtered and the solvent was evaporated under reduced pressure. The crude product was purified by column chromatography (EtOAc/hexane 1:6) to afford pyrrolidine **6** as a colorless oil (530 mg, 83%). R_F = 0.29 (EtOAc/hexane 1:6); $[\alpha]_D = -70.8$ (c = 1.0; $CHCl_3$); ^{1}H NMR: (400 MHz; $CDCl_3$), δ/ppm: 7.33–7.18 (m, 5H, Ar), 4.56 (dd, 1H, J = 6.4, 4.6 Hz, H-6a), 4.49 (dd, 1H, J = 6.4, 4.7 Hz, H-3a), 4.03 (d, 1H, J = 13.5 Hz, NCH_2Ar), 3.09 (d, 1H, J = 13.5 Hz, NCH_2Ar), 3.01 (d, 1H, J = 11.1 Hz, H-6), 2.24–2.14 (m, 1H, H-4), 1.97 (dd, 1H, J = 11.1, 4.6 Hz, H-6'), 1.55 and 1.33 [2s, each 3H, C(CH_3)$_2$], 1.23 (d, 3H, J = 6.3 Hz, CH_3); ^{13}C NMR: (100 MHz; $CDCl_3$), δ/ppm: 138.8, 128.6, 128.2, 126.8 (Ar), 111.1 (C(CH_3)$_2$), 82.5 (C-3a), 78.1 (C-6a), 62.8 (C-4), 59.4 (C-6), 56.6 (NCH$_2$Ar), 26.4 and 25.8 [C(CH$_3$)$_2$], 12.6 (CH$_3$); HRMS (ESI, m/z): calculated for $C_{15}H_{21}NO_2$ $[M+H]^+$: 248.1645; found 248.1648.

(2*R*,3*S*,4*R*)-1-Benzyl-2-methylpyrrolidine-3,4-diol (7)

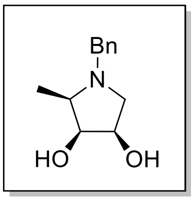

In a manner similar to [2], 20% HCl (1.5 mL) was added to a stirred solution of acetonide **6** (64 mg, 0.26 mmol) in MeOH (3 mL) while cooling to 0 °C (ice–water bath). After 15 min of stirring, the ice–water bath was removed and the reaction mixture was stirred at rt overnight. Next, HCl was carefully neutralized with solid Na_2CO_3 (0.6 g). The resulting suspension was filtered, the filtration cake was washed with MeOH (5 mL) and the filtrate was concentrated. The residue was suspended in CH_2Cl_2 (10 mL), filtered and the solvent was evaporated under reduced pressure. The crude product was purified by column chromatography on silica gel (MeOH/$CHCl_3$ 1:10 containing 0.5% (v/v) conc. NH_3) to afford pyrrolidine **7** as a yellow oil (38 mg, 70%). R_F = 0.28 (MeOH/$CHCl_3$ 1:10 containing 0.5 % (v/v) conc. NH_3); $[\alpha]_D = -41.8$ (c = 1.0; $CHCl_3$); ^{1}H NMR: (400 MHz; $CDCl_3$), δ/ppm: 7.36–7.19 (m, 5H, Ar), 4.11 (td, 1H, J = 5.8, 2.1 Hz, H-4), 3.99 (t, 1H, H-3), 3.97 (d, 1H, J = 13.0 Hz, NCH_2Ar), 3.17 (d, 1H, J = 13.0 Hz, NCH_2Ar), 2.77 (dd, 1H, J = 10.8, 2.0 Hz, H-5), 2.51 (p, 1H, J = 6.3 Hz, H-2), 2.32 (dd, 1H, J = 10.9, 6.0 Hz, H-5'), 1.21 (d, 3H, J = 6.5 Hz, CH_3); ^{13}C NMR: (100 MHz; $CDCl_3$), δ/ppm: 138.1, 129.0, 128.3, 127.2 (Ar), 73.6 (C-3), 70.0 (C-4), 62.0 (C-2), 59.4 (C-5), 57.2 (NCH$_2$Ar), 13.3 (CH$_3$); HRMS (ESI, m/z): calculated for $C_{12}H_{17}NO_2$ $[M+H]^+$: 208.1332; found 208.1335.

(2*R*,3*S*,4*R*)-1-(4-Iodobenzyl)-2-methylpyrrolidine-3,4-diol (8)

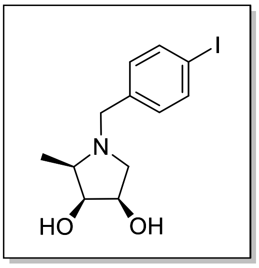

In a manner similar to [2], a suspension of 10% Pd-C (25 mg, 10 wt %) and pyrrolidine **6** (250 mg, 1.01 mmol) in MeOH (10 mL) was stirred at rt under a hydrogen atmosphere (balloon) for 48 h. The catalyst was filtered off, the filtrate was cooled to 0 °C (ice–water bath) and conc. HCl (0.38 mL) was added. The ice–water bath was removed and the reaction mixture was stirred at 40 °C for 2 h. The solvents were evaporated to dryness to give the corresponding hydrochloride as white solid (174 mg), which was used in next reaction without further purification and characterization. To a suspension of the hydrochloride in anhydrous DMF (10 mL), K_2CO_3 (418 mg, 3.03 mmol, 3 equiv) and 4-iodobenzyl bromide (359 mg, 1.21 mmol, 1.2 equiv) were added and the reaction mixture was stirred at rt overnight. Next, the reaction mixture was partitioned between water (40 mL) and EtOAc (40 mL), the organic layer was separated and washed with water (40 mL). The organic layer was dried over Na_2SO_4, filtered and the solvent was evaporated under reduced pressure. The crude product was purified by column chromatography on silica gel (MeOH/$CHCl_3$ 1:10 containing 0.5% (v/v) conc. NH_3) to afford pyrrolidine **8** as an

orange solid (207 mg, 61%). R_F = 0.18 (MeOH/$CHCl_3$ 1:10 containing 0.5 % (v/v) conc. NH_3); $[\alpha]_D$ = −25.6 (*c* = 1.0; $CHCl_3$); ^{1}H NMR: (400 MHz; $CDCl_3$), δ/ppm: 7.63 (d, 2H, *J* = 8.7 Hz, Ar), 7.04 (d, 2H, *J* = 8.2 Hz, Ar), 4.13 (td, 1H, *J* = 5.8, 2.2 Hz, H-4), 4.00 (t, 1H, *J* = 5.5 Hz, H-3), 3.91 (d, 1H, *J* = 13.2 Hz, NCH_2Ar), 3.12 (d, 1H, *J* = 13.2 Hz, NCH_2Ar), 2.78 (dd, 1H, *J* = 10.8, 2.1 Hz, H-5), 2.58 (br s, 2H, OH), 2.52 (p, 1H, *J* = 7.7 Hz, H-2), 2.31 (dd, 1H, *J* = 10.8, 6.0 Hz, H-5'), 1.19 (d, 3H, *J* = 6.5 Hz, CH_3); ^{13}C NMR: (100 MHz; $CDCl_3$), δ/ppm: 137.9, 137.5, 130.9, 92.5 (Ar), 73.6 (C-3), 70.0 (C-4), 62.1 (C-2), 59.4 (C-5), 56.8 (NCH_2Ar), 13.3 (CH_3); HRMS (ESI, m/z): calculated for $C_{12}H_{16}INO_2$ $[M+Na]^+$: 356.0118; found 356.0128.

(2*R*,3*S*,4*R*)-2-Methyl-1-(naphthalen-2-ylmethyl)pyrrolidine-3,4-diol (9)

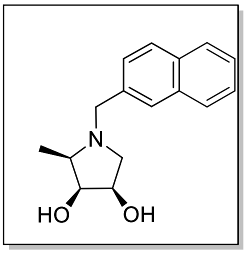

In a manner similar to [2], a suspension of 10% Pd-C (6.5 mg, 10 wt %) and pyrrolidine **6** (65 mg, 0.26 mmol) in MeOH (5 mL) was stirred at rt under a hydrogen atmosphere (balloon) for 48 h. The catalyst was filtered off, the filtrate was cooled to 0 °C (ice–water bath) and conc. HCl (0.10 mL) was added. The ice–water bath was removed and the reaction mixture was stirred at 40 °C for 2 h. The solvents were evaporated to dryness to give the corresponding hydrochloride as white solid (37.7 mg), which was used in next reaction without further purification and characterization. To a suspension of the hydrochloride in anhydrous DMF (2 mL), K_2CO_3 (102 mg, 0.74 mmol, 3 equiv) and 2-(bromomethyl)naphthalene (65 mg, 0.29 mmol, 1.2 equiv) were added and the reaction mixture was stirred at rt overnight. Next, the reaction mixture was partitioned between water (20 mL) and EtOAc (20 mL), the organic layer was separated and washed with water (20 mL). The organic layer was dried over Na_2SO_4, filtered and the solvent was evaporated under reduced pressure. The crude product was purified by column chromatography on silica gel ($CHCl_3$/MeOH 10:1 containing 0.5% (v/v) conc. NH_3) to afford pyrrolidine **9** as a yellow oil (47.4 mg, 70%). R_F 0.16 ($CHCl_3$/MeOH 10 :1 containing 0.5 % (v/v) conc. NH_3); $[\alpha]_D$ = –55.4 (*c* 0.25; MeOH); ^{1}H NMR: (400 MHz; CD_3OD), δ/ppm: 7.89-7.79 (m, 4H, Ar), 7.55-7.46 (m, 3H, Ar), 4.19 (td, 1H, *J* = 5.8, 2.2 Hz, H-4), 4.15 (d, 1H, *J* = 13.2 Hz, NCH_2Ar), 4.02 (t, 1H, *J* = 5.5 Hz, H-3), 3.44 (d, 1H, *J* = 13.0 Hz, NCH_2Ar), 2.85 (dd, 1H, *J* = 10.8, 3.4 Hz, H-5), 2.67 (dt, 1H, *J* = 12.4, 6.2 Hz, H-2), 2.57 (dd, 1H, *J* = 10.8, 6.8 Hz, H-5'), 1.26 (d, 3H, *J* = 6.5 Hz, CH_3); ^{13}C NMR: (100 MHz; CD_3OD), δ/ppm: 137.1, 134.8, 134.3, 128.9, 128.8, 128.7, 128.6, 128.5, 127.0, 126.7 (Ar), 74.4 (C-3), 70.6 (C-4), 64.2 (C-2), 60.6 (C-5), 59.4 (NCH_2Ar), 13.3 (CH_3); HRMS (ESI, m/z): calculated for $C_{16}H_{19}NO_2$ $[M+Na]^+$: 258.1489; found 258.1488.

(2*R*,3*S*,4*R*)-2-Methylpyrrolidine-3,4-diol hydrochloride (10)

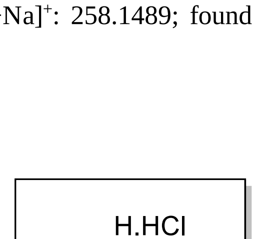

A suspension of 10% Pd-C (9.3 mg, 10 wt %) and pyrrolidine **7** (93.3 mg, 0.45 mmol, 1 equiv) in MeOH (10 mL) was stirred at rt under a hydrogen atmosphere (balloon) for 3 h. The catalyst was filtered, the filtrate was cooled to 0 °C (ice–water bath) and conc. HCl (0.15 mL) was added. The ice–water bath was removed and the reaction mixture was stirred at 40 °C for 2 h. The solvents were evaporated and the product was lyophilized to give **10** as a yellowish solid (47.1 mg, 68%). R_F 0.03 (EtOAc/MeOH 3:1 containing 0.5% (v/v) conc. NH_3); $[\alpha]_D$ = +25.7 (*c* 0.29; H_2O); ^{1}H NMR: (400 MHz; CD_3OD), δ/ppm: 4.44 (td, 1H, *J* = 7.7, 4.0 Hz, H-4), 4.05 (t, 1H, *J* = 3.7 Hz, H-3), 3.63 (qd, 1H, *J* = 6.8, 3.4 Hz, H-2), 3.46 (dd, 1H, *J* = 11.7, 7.9 Hz, H-5), 3.12 (dd, 1H, *J* = 11.7, 7.4 Hz, H-5'), 1.43 (d, 3H, *J* = 6.5 Hz, CH_3). ^{13}C NMR: (100 MHz; CD_3OD),

δ/ppm: δ 72.6 (C-3), 72.1 (C-4), 48.5 (C-2), 59.2 (C-5), 12.2 (CH_3); HRMS (ESI, *m/z*): calculated for $C_5H_{11}NO_2$ $[M+H]^+$: 118.0863; found 118.0863.

(3a*S*,4*R*,6a*R*)-Benzyl 2,2-dimethyl-4-((trityloxy)methyl)dihydro-3a*H*-[1,3]dioxolo[4,5-*c*]pyrrol-5(4*H*)-carboxylate (11)

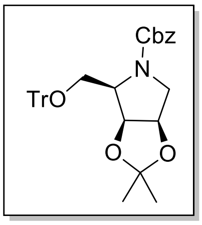

In a manner similar to [2], a suspension of 10% Pd-C (0.5 g, 10 wt %) and *N*-benzylpyrrolidine **3** (5.06 g, 10.0 mmol) in MeOH (10 mL) was stirred at rt under a hydrogen atmosphere (balloon) for 48 h. The catalyst was filtered off and the solvent was evaporated under reduced pressure. The residue was dissolved in CH_2Cl_2 (70 mL) and Et_3N (3.48 mL, 25.0 mmol) was added to the reaction mixture. While cooling to 0 °C (ice–water bath), CbzCl (45% in toluene, 7.50 mL, 0.02 mol) was added to the reaction mixture. After 15 min of stirring, the ice–water bath was removed and the reaction mixture was stirred at rt for 2 h. Next, the reaction mixture was washed with water (2 × 70 mL), the layers separated and the aqueous layer was extracted with CH_2Cl_2 (50 mL). The combined organic extracts were dried over Na_2SO_4, filtered and the solvent was evaporated under reduced pressure. The crude product was purified by column chromatography on silica gel (EtOAc/hexanes 1:4) to afford carboxylate **11** as a white solid (4.99 g, 91%). R_F = 0.21 (EtOAc/hexane 1:4); $[\alpha]_D$ = −26.6 (*c* = 1.0; $CHCl_3$); ^{1}H NMR: (400 MHz; $CDCl_3$), δ/ppm: 7.51–7.42 (m, 5H, Ar), 7.41–7.12 (m, 15H, Ar), 5.16 (d, 1H, *J* = 12.3 Hz, OC*H*$_2$Ph), 4.99 (d, 1H, *J* = 12.3 Hz, OC*H*$_2$Ph), 4.79 (t, 1H, *J* = 6.7 Hz, H-3a), 4.69 (td, 1H, *J* = 7.1, 4.3 Hz, H-6a), 4.28 (q, 1H, *J* = 6.7 Hz, H-4), 3.92 (dd, 1H, *J* = 12.2, 7.3 Hz, H-6), 3.39 (t, 1H, *J* = 7.8 Hz, C*H*$_2$OTr), 3.31 (dd, 1H, *J* = 9.1, 6.8 Hz, C*H*$_2$OTr), 3.23 (dd, 1H, *J* = 12.4, 4.4 Hz, H-6'), 1.29 and 1.24 [2s, each 3H, C(C*H*$_3$)$_2$]; ^{13}C NMR: (100 MHz; $CDCl_3$), δ/ppm: 154.7 (CO), 144.2, 136.6, 128.9, 128.5, 128.1, 127.7, 126.9 (Ar), 113.2 [*C*(CH$_3$)$_2$], 86.8 (*C*Ph$_3$), 79.5 (C-3a), 77.7 (C-6a), 67.1 (O*C*H$_2$Ph), 61.4 (*C*H$_2$OTr), 58.9 (C-4), 50.4 (C-6), 26.1 and 25.0 [C(*C*H$_3$)$_2$]; HRMS (ESI, m/z): calculated for $C_{35}H_{35}NO_5$ $[M+Na]^+$: 572.2407; found 572.2411.

(3a*S*,4*R*,6a*R*)-Benzyl 4-(hydroxymethyl)-2,2-dimethyldihydro-3a*H*-[1,3]dioxolo[4,5-*c*]pyrrol-5(4*H*)-carboxylate (12)

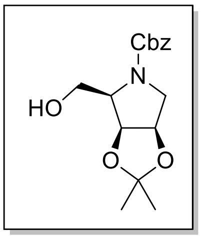

In a manner similar to [2], PTSA·H_2O (24 mg, 0.13 mmol) was added to a stirred solution of trityl ether **11** (1.74 g, 3.17 mmol) in CH_2Cl_2 (30 mL) and MeOH (1 mL) and the reaction mixture was stirred at rt for 20 min. Next, PTSA was neutralized with conc. NH_3 solution (0.24 mL), the reaction mixture was dried over Na_2SO_4, filtered and the solvent was evaporated under reduced pressure. The crude product was purified by column chromatography on silica gel (EtOAc/hexane 1:1) to afford alcohol **12** as a colorless oil (874 mg; 90%). R_F = 0.25 (EtOAc/hexane 1:1); $[\alpha]_D$ = −22.6 (*c* = 1.0; $CHCl_3$); ^{1}H NMR: (400 MHz; $CDCl_3$), δ/ppm: 7.45–7.17 (m, 5H, Ar), 5.16 (d, 1H, *J* = 12.3 Hz, OC*H*$_2$Ph), 5.12 (d, 1H, *J* = 12.3 Hz, OC*H*$_2$Ph), 4.81 (br s, 1H), 4.72 (br s, 1H), 4.35 (br s, 1H), 3.90 (br s, 3H), 3.65 (br s, 2H), 1.49 and 1.33 [2s, each 3H, C(C*H*$_3$)$_2$]; ^{13}C NMR: (100 MHz; $CDCl_3$), δ/ppm: C=O not observed, 136.2, 128.6, 128.2, 128.1 (Ar), 112.6 [*C*(CH$_3$)$_2$], 80.4, 77.6, 67.4 (O*C*H$_2$Ph), 62.1, 51.8, 26.3 and 24.8 [C(*C*H$_3$)$_2$]; HRMS (ESI, m/z): calculated for $C_{16}H_{21}NO_5$ $[M+Na]^+$: 330.1312; found 330.1323;.

(3a*S*,4*R*,6a*R*)-Benzyl 4-((*S*)-1-hydroxyethyl)-2,2-dimethyldihydro-3a*H*-[1,3]dioxolo[4,5-*c*]pyrrol-5(4*H*)-carboxylate (13)

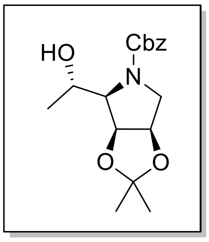

In a manner similar to [2], DMP (1.72 g, 4.05 mmol) was added to a stirred solution of alcohol **12** (860 mg, 2.7 mmol) in CH_2Cl_2 (27 mL) and the reaction mixture was stirred at rt for 1 h. The reaction mixture was washed with saturated $NaHCO_3$ solution (2 × 15 mL) and 10% $Na_2S_2O_3$ solution (15 mL). The organic layer was dried over Na_2SO_4, filtered and the solvent was evaporated to give the corresponding aldehyde (887 mg), which was used in next reaction without further purification and characterization. MeMgBr (3 M in Et_2O, 3.15 mL, 9.45 mmol) was added to the solution of the aldehyde in anhydrous Et_2O (27 mL) while cooling to 0 °C (ice–water bath) under a nitrogen atmosphere. After 15 min of stirring, the ice–water bath was removed and the reaction mixture was stirred at rt for 1 h. The reaction mixture was quenched with saturated NH_4Cl solution (10 mL) and water (30 mL) was added. The layers were separated and the aqueous layer was extracted with Et_2O (20 mL). The combined organic extracts were dried over Na_2SO_4, filtered and the solvent was evaporated under reduced pressure. The crude product was purified by column chromatography (EtOAc/hexane 1:2) to afford pyrrolidine **13** as a white solid (549 mg, 63%). R_F = 0.16 (EtOAc/hexane 1:2); $[\alpha]_D$ = −5.0 (*c* = 1.0; $CHCl_3$); ^1H NMR: (400 MHz; $CDCl_3$), δ/ppm: 7.39–7.25 (m, 5H, Ar), 5.14 (s, 2H, OCH_2Ph), 4.72–4.60 (m, 2H), 4.21 (pd, 1H, *J* = 6.4, 3.8 Hz, C*H*OH), 3.64 (br s, 3H), 1.47 and 1,31 [2s, each 3H, $C(CH_3)_2$], 1.34 (d, 3H, *J* = 6.3 Hz, CH_3); ^{13}C NMR: (100 MHz; $CDCl_3$), δ/ppm: 136.2, 128.6, 128.2, 128.0 (Ar), 112.5 [$C(CH_3)_2$], 80.4, 67.5 (OCH_2Ph), 66.7, 52.5, 26.8 and 24.9 [$C(CH_3)_2$], 20.4 (CH_3); HRMS (ESI, m/z): calculated for $C_{17}H_{23}NO_5$ $[M+Na]^+$: 344.1468; found 344.1483.

General method for alkylation (Method A)

In a manner similar to [2], a suspension of 10% Pd-C (10 wt % of **13**) and alcohol **13** (0.35 mmol, 1 equiv) in MeOH (5 mL) was stirred at rt under a hydrogen atmosphere (balloon) for 2 h. The catalyst was filtered off and the solvent was evaporated under reduced pressure. The crude amine was dissolved in anhydrous DMF (5 mL/0.35mmol of amine) and the reaction mixture was cooled to 0 °C (ice–water bath). K_2CO_3 (1.4 equiv) and the corresponding bromide (1 equiv) were added and the reaction mixture was stirred for 15 min. Then, the ice–water bath was removed and the reaction mixture was stirred at rt overnight. The reaction mixture was partitioned between water (50 mL) and EtOAc (25 mL) and the layers were separated. The organic layer was washed with water (50 mL) and brine (5 mL), dried over Na_2SO_4, filtered and the solvent was evaporated under reduced pressure. The crude product was purified by column chromatography on silica gel.

(*S*)-1-((3a*S*,4*R*,6a*R*)-5-Benzyl-2,2-dimethyltetrahydro-3a*H*-[1,3]dioxolo[4,5-*c*]pyrrol-4-yl)ethanol (14)

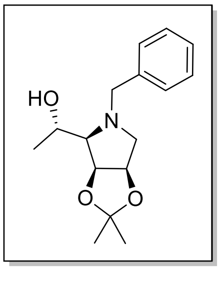

Reduction of **13** (155 mg, 0.48 mmol, 1 equiv) afforded the crude amine, which was directly reacted with K_2CO_3 (93 mg) and BnBr (57 µL) following the general procedure Method A. The crude product was purified by column chromatography on silica gel ($MeOH/CHCl_3$ 1:60) to afford pyrrolidine **14** (46 mg, 35%) as colorless oil. R_F = 0.15 ($MeOH/CHCl_3$ 1:60); $[\alpha]_D$ = −59.9 (*c* = 1.0; $CHCl_3$); ^1H NMR: (400 MHz; $CDCl_3$), δ/ppm: 7.38–7.20 (m, 5H, Ar), 4.64 (dd, 1H, *J* = 6.4, 4.9 Hz, H-3a), 4.58–4.53 (m, 1H, H-6a), 4.33 (d, 1H, *J* = 13.3 Hz, NCH_2Ar), 4.26–4.17 (m, 1H, C*H*OH), 3.20 (d, 1H, *J* = 13.3 Hz, NCH_2Ar), 3.04 (d, 1H, *J* = 11.4 Hz, H-6), 2.54 (br s, 1H, OH), 2.33 (t, 1H, *J* = 4.9 Hz, H-4), 2.14 (dd,

1H, J = 11.4, 4.9 Hz, H-6'), 1.53 and 1.29 [2s, each 3H, C(CH_3)$_2$], 1.41 (d, 3H, J = 6.6 Hz, CH_3); ^{13}C NMR: (100 MHz; $CDCl_3$), δ/ppm: 139.0, 128.5, 128.4 (Ar), 111.2 [C(CH_3)$_2$], 81.8 (C-3a), 77.8 (C-6a), 71.8 (C-4), 66.5 (CHOH), 59.2 (C-6), 58.8 (NCH$_2$Ar), 26.3 and 22.3 [C(CH$_3$)$_2$], 25.0 (CH$_3$); HRMS (ESI, m/z): calculated for $C_{16}H_{23}NO$ [M+H]$^+$: 278.1751; found 278.1758.

(*S*)-1-((3a*S*,4*R*,6a*R*)-5-(4-Iodobenzyl)-2,2-dimethyltetrahydro-3a*H*-[1,3]dioxolo[4,5-*c*]pyrrol-4-yl)ethanol (15)

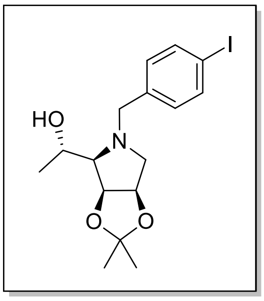

Reduction of **13** (113 mg, 0.35 mmol, 1 equiv) afforded the crude amine, which was directly reacted with K_2CO_3 (67 mg) and 4-iodobenzyl bromide (104 mg) following the general procedure Method A. The crude product was purified by column chromatography on silica gel (MeOH/$CHCl_3$ 1:60) to afford pyrrolidine **15** as a colorless oil (88 mg, 62%). R_F = 0.16 (MeOH/$CHCl_3$ 1:60); $[\alpha]_D$ = −36.3 (c = 1.0; $CHCl_3$); ^{1}H NMR: (400 MHz; $CDCl_3$), δ/ppm: 7.60 (d, 2H, J = 8.3 Hz, Ar), 7.10 (d, 2H, J = 8.2 Hz, Ar), 4.63 (dd, 1H, J = 6.4, 4.8 Hz, H-3), 4.55 (dd, 1H, J = 6.3, 4.9 Hz, H-2), 4.32 (d, 1H, J = 13.5 Hz, NCH_2Ar), 4.25–4.16 (m, 1H, H-5), 3.12 (d, 1H, J = 13.5 Hz, NCH_2Ar), 3.00 (d, 1H, J = 11.3 Hz, H-1a), 2.36 (s, 1H, OH), 2.30 (t, 1H, J = 5.0 Hz, H-4), 2.08 (dd, 1H, J = 11.3, 4.8 Hz, H-1b), 1.52 and 1.28 [2s, each 3H, C(CH_3)$_2$], 1.40 (d, 3H, J = 6.6 Hz, CH_3); ^{13}C NMR: (100 MHz; $CDCl_3$), δ/ppm: 138.9, 137.4, 130.4 (Ar), 111.2 [C(CH_3)$_2$], 92.1 (Ar), 81.8 (C-3), 77.6 (C-2), 71.7 (C-4), 66.9 (C-5), 59.3 (C-1), 58.0 (NCH_2Ar), 26.3 and 25.0 [C(CH$_3$)$_2$], 22.3 (CH_3); HRMS (ESI, m/z): calculated for $C_{16}H_{22}INO_3$ [M+Na]$^+$: 426.0537; found 426.0551.

(*S*)-1-((3a*S*,4*R*,6a*R*)-2,2-Dimethyl-5-(naphthalen-2-ylmethyl)tetrahydro-3a*H*-[1,3]dioxolo[4,5-*c*]pyrrol-4-yl)ethanol (16)

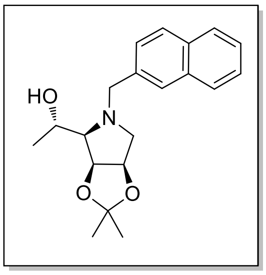

Reduction of **13** (140 mg, 0.44 mmol, 1 equiv) afforded the crude amine, which was directly reacted with K_2CO_3 (84 mg) and 2-(bromomethyl)naphthalene (96 mg) following the general procedure Method A. The crude product was purified by column chromatography on silica gel (EtOAc/hexane 8:1→4:1) to afford pyrrolidine **16** as a colorless oil, (64.4 mg, 46%). R_F = 0.15 (MeOH/$CHCl_3$ 1:60); $[\alpha]_D$ = –137.6 (c 0.22; $CHCl_3$); ^{1}H NMR: (400 MHz; $CDCl_3$), δ/ppm: 7.86-7.71 (m, 4H, Ar), 7.53 (dd, 1H, J = 8.4, 1.6 Hz, Ar), 7.48-7.40 (m, 2H, Ar), 4.66 (dd, 1H, J = 6.4, 4.9 Hz, H-3a), 4.61-4.55 (m, 1H, H-6a), 4.51 (d, 1H, J = 13.3 Hz, NCH_2Ar), 4.30-4.24 (m, 1H, CHOH), 3.41 (d, 1H, J = 13.3 Hz, NCH_2Ar), 3.06 (d, 1H, J = 11.5 Hz, H-6), 2.41 (t, 1H, J = 5.0 Hz, H-4), 2.22 (dd, 1H, J = 11.5, 4.9 Hz, H-6'), 1.56 and 1.30 [2s, each 3H, C(CH_3)$_2$), 1.45 (d, 3H, J = 6.6 Hz, CH_3); ^{13}C NMR: (100 MHz; $CDCl_3$), δ/ppm: 136.6, 133.5, 132.9, 128.2, 127.9, 127.8, 127.1, 127.0, 126.1, 125.7 (Ar), 111.4 [C(CH_3)$_2$], 81.9 (C-3a), 77.9 (C-6a), 72.0 (C-4), 66.7 (CHOH), 59.3 (C-6), 59.0 (NCH$_2$Ar), 26.4 and 22.4 [C(CH$_3$)$_2$], 25.1 (CH$_3$); HRMS (ESI, m/z): calculated for $C_{20}H_{25}NO_3$ [M+H]$^+$: 328.1907; found 328.1905.

General procedure for deprotection (Method B)

In a manner similar to [2], 20% HCl (1 mL) was added to a stirred solution of protected acetonide (0.15 mmol) in MeOH (2 mL) while cooling to 0 °C (ice–water bath). After 15 min of stirring, the ice–water bath was removed and the reaction mixture was stirred at rt for 72 h. Next, HCl was carefully neutralized with solid Na_2CO_3 (950 mg, 1.5 equiv to HCl). The resulting suspension was filtered, the filtration cake was washed with MeOH (5 mL) and the filtrate was concentrated. The residue was suspended in DCM (5 mL), filtered and the solvent was evaporated under reduced pressure. The crude product was purified by column chromatography.

(2*R*,3*S*,4*R*)-1-Benzyl-2-((*S*)-1-hydroxyethyl)pyrrolidine-3,4-diol (17)

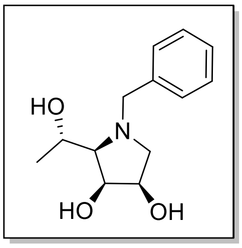

Deprotection of acetonide **14** (49 mg, 0.16 mmol) following the general procedure Method B afforded after purification by column chromatography on silica gel ($MeOH/CHCl_3$ 1:20 containing 0.5% (v/v) conc. NH_3) triol **17** as a colorless oil (29 mg, 78%). R_F = 0.10 ($MeOH/CHCl_3$ 1:20 containing 0.5 % (v/v) conc. NH_3); $[\alpha]_D = -19.0$ (c = 0.5; $CHCl_3$); ^{1}H NMR: (400 MHz; $CDCl_3$), δ/ppm: 7.37–7.23 (m, 5H, Ar), 4.33 (dd, 1H, J = 8.7, 4.9 Hz, H-3), 4.15–4.07 (m, 2H, NCH_2Ar, C*H*OH), 4.05 (td, 1H, J = 4.7, 2.0 Hz, H-4), 3.60 (d, 1H, J = 13.5 Hz, NCH_2Ar), 3.01 (dd, 1H, J = 11.3, 1.9 Hz, H-5), 2.97 (dd, 1H, J = 8.7, 1.5 Hz, H-2), 2.56 (dd, 1H, J = 11.3, 4.4 Hz, H-5'), 1.32 (d, 3H, J = 6.7 Hz, CH_3); ^{13}C NMR: (100 MHz; $CDCl_3$), δ/ppm: 138.5, 128.5, 128.4, 127.4 (Ar), 73.4 (C-3), 71.5 (C-4), 68.2 (C-2), 65.0 (*C*HOH), 62.4 (NCH_2Ar), 58.4 (C-5), 21.9 (*C*H_3); HRMS (ESI, m/z): calculated for $C_{13}H_{19}NO_3$ $[M+H]^+$: 238.1438; found 238.1442.

(2*R*,3*S*,4*R*)-2-((*S*)-1-Hydroxyethyl)-1-(4-iodobenzyl)pyrrolidine-3,4-diol (18)

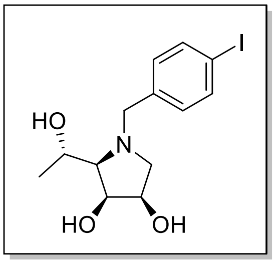

Deprotection of acetonide **15** (62 mg, 0.15 mmol) following the general procedure Method B afforded after purification by column chromatography on silica gel ($MeOH/CHCl_3$ 1:20 containing 0.5% (v/v) conc. NH_3) triol **18** as white crystals (38 mg, 68%). R_F = 0.11 ($MeOH/CHCl_3$ 1:20 containing 0.5 % (v/v) conc. NH_3); $[\alpha]_D = -11.2$ (c = 1.0; $CHCl_3$); ^{1}H NMR: (400 MHz; $CDCl_3$), δ/ppm: 7.66 (d, 2H, J = 8.3 Hz, Ar), 7.08 (d, 2H, J = 8.2 Hz, Ar), 4.34 (dd, 1H, J = 8.7, 4.9 Hz, H-3), 4.11 (qd, 1H, J = 6.7, 1.5 Hz, C*H*OH), 4.08–3.99 (m, 2H, H-2, NCH_2Ar, H-2), 3.54 (d, 1H, J = 13.8 Hz, NCH_2Ar), 2.98 (dd, 1H, J = 11.2, 1.9 Hz, H-5), 2.95 (dd, 1H, J = 8.8, 1.6 Hz, H-2), 2.50 (dd, 1H, J = 11.2, 4.3 Hz, H-5'), 1.31 (d, 3H, J = 6.7 Hz, CH_3); ^{13}C NMR: (100 MHz; $CDCl_3$), δ/ppm: 138.3, 137.6, 130.2, 92.5 (Ar), 73.4 (C-3), 71.5 (C-4), 68.3 (C-2), 65.2 (*C*HOH), 61.8 (N*C*H_2Ar), 58.4 (C-5), 21.8 (*C*H_3); HRMS (ESI, m/z): calculated for $C_{13}H_{18}INO_3$ $[M+Na]^+$: 386.0224; found 386.0222.

(2*R*,3*S*,4*R*)-2-((*S*)-1-Hydroxyethyl)-1-(naphthalen-2-ylmethyl)pyrrolidine-3,4-diol (19)

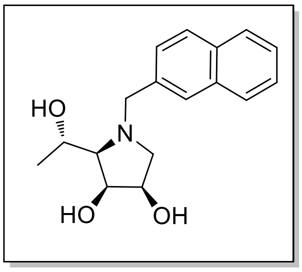

Deprotection of acetonide **16** (57 mg, 0.17 mmol) following the general procedure Method B afforded after purification by column chromatography on silica gel ($MeOH/CHCl_3$ 1:20 containing 0.5% (v/v) conc. NH_3) triol **19** as a yellowish oil (28 mg, 56%). R_F = 0.10 ($MeOH/CHCl_3$ 1:20 containing 0.5 % (v/v) conc. NH_3); $[\alpha]_D = -20.6$ (c 0.24; CH_3OH); ^{1}H NMR: (400 MHz; CD_3OD), δ/ppm: 7.89-7.76 (m, 4H, Ar), 7.60 (dd, 1H, J = 8.5, 1.5 Hz, Ar), 7.50-7.35 (m, 2H, Ar), 4.37 (d, 1H, J = 13.5 Hz, NCH_2Ar), 4.25 (dd, 1H, J = 7.2, 4.7 Hz, H-3), 4.16-4.09 (m, 2H,

H-4, C*H*OH), 3.75 (d, 1H, *J* = 13.5 Hz, NCH_2Ar), 2.95-2.88 (m, 2H, H-5, H-2), 2.66 (dd, 1H, *J* = 10.9, 5.4 Hz, H-5‘), 1.35 (d, 3H, *J* = 6.5 Hz, CH_3); ^{13}C NMR: (100 MHz; CD_3OD), δ/ppm: 138.3, 134.9, 134.3, 128.9, 128.7, 128.6, 128.3, 127.0, 126.6 (Ar), 74.1 (C-3), 72.2 (C-4), 71.6 (C-2), 67.9 (*C*HOH), 63.4 (N*C*H_2Ar), 59.0 (C-5), 21.4 (*C*H_3); HRMS (ESI, *m/z*): calculated for $C_{17}H_{21}NO_3$ [*M*+H]$^+$: 288.1594; found 288.1595.

(2*R*,3*S*,4*R*)-2-((*S*)-1-Hydroxyethyl)pyrrolidine-3,4-diol·HCl (20)

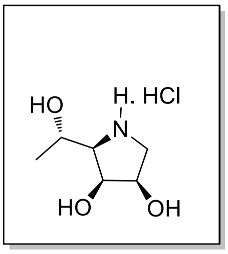

A suspension of 10% Pd-C (6.0 mg, 20 wt %) and pyrrolidine **17** (30.3 mg, 0.13 mmol) in MeOH (5 mL) was stirred at rt under a hydrogen atmosphere (balloon) for 5 h. The catalyst was filtered off, the filtrate was cooled to 0 °C (ice–water bath) and acidified with conc. HCl (0.05 mL). The ice–water bath was removed and the solvents were evaporated. The product was lyophilized to give **20** as a yellowish solid (18 mg, 76%). R_F = 0.05 (MeOH/EtOAc 1:3 containing 0.5 % (v/v) conc. NH_3); $[\alpha]_D$ = +196.6 (*c* 0.31; H_2O). ^{1}H NMR: (400 MHz; D_2O), δ/ppm: 4.49 (td, 1H, *J* = 8.4, 3.9 Hz, H-4), 4.26 (t, 1H, *J* = 3.6 Hz, H-3), 4.15 (dq, 1H, *J* = 9.1, 6.3 Hz, H- C*H*OH), 3.55 (dd, 1H, *J* = 12.0, 8.3 Hz, H-5), 3.44 (dd, 1H, *J* = 9.2, 3.2 Hz, H-2), 3.16 (dd, 1H, *J* = 12.0, 8.6 Hz, H-5’), 1.29 (d, 3H, *J* = 6.3 Hz, CH_3); ^{13}C NMR: (100 MHz; D_2O), δ/ppm: 70.3 (C-4), 69.7 (C-3), 67.3 (C-2), 64.3 (*C*HOH), 46.4 (C-5), 19.6 (*C*H_3); HRMS (ESI, m/z): calculated for $C_6H_{13}N_3O_3$ [*M*+H]$^+$: 148.0968; found 148.0969.

(3a*S*,3b*R*,4*S*,8a*R*)-2,2,4-Trimethyltetrahydro-[1,3]dioxolo[4',5':3,4]pyrrolo[1,2-*c*]oxazol-6(3a*H*)-one (21)

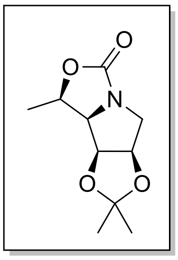

In a manner similar to [2], anhydrous pyridine (25 µL, 0.31 mmol) and Tf_2O (65 µL, 0.31 mmol) were added to a solution of alcohol **13** (50 mg, 155 µmol) in anhydrous CH_2Cl_2 (2 mL) under a nitrogen atmosphere while cooling to 0 °C (ice–water bath) and the reaction mixture was stirred for 1.5 h. Then, the reaction mixture was partitioned between water (10 mL) and $CHCl_3$ (2 mL) and the layers were separated. The aqueous layer was extracted with $CHCl_3$ (10 mL), the combined organic extracts were dried over Na_2SO_4, filtered and the solvent was evaporated under reduced pressure. The crude product was purified by column chromatography (EtOAC/hexane 2:1) to afford cyclic carbamate **21** as white crystals (21 mg, 64%). R_F = 0.10 (EtOAc/hexane 2:1); $[\alpha]_D$ = −14.4 (*c* = 1.0; $CHCl_3$); ^{1}H NMR: (400 MHz; $CDCl_3$), δ/ppm: 4.86 (p, 1H, *J* = 6.7 Hz, H-4), 4.79 (td, 1H, *J* = 5.3, 1.1 Hz, H-8a), 4.61 (dd, 1H, *J* = 5.1, 3.3 Hz, H-3a), 3.88 (dd, 1H, *J* = 13.3, 1.1 Hz, H-8), 3.63 (dd, 1H, *J* = 6.9, 3.3 Hz, H-3b), 3.13 (dd, 1H, *J* = 13.3, 5.5 Hz, H-8´), 1.65 (d, 3H, *J* = 6.7 Hz; CH_3), 1.46 and 1.28 [2s, each 3H, C(CH_3)$_2$]; ^{13}C NMR: (100 MHz; $CDCl_3$), δ/ppm: 161.2 (CO), 112.9 [*C*(CH_3)$_2$], 81.9 (C-8a), 80.0 (C-3a), 72.1 (C-4), 65.9 (C-3b), 52.5 (C-8), 26.5 and 24.5 [C(*C*H_3)$_2$], 14.6 (*C*H_3); HRMS (ESI, m/z): calculated for $C_{10}H_{15}NO_4$ [*M*+H]$^+$: 214.1074; found 214.1075.

(*R*)-1-((3a*S*,4*R*,6a*R*)-2,2-Dimethyltetrahydro-3a*H*-[1,3]dioxolo[4,5-*c*]pyrrol-4-yl)ethanol (22)

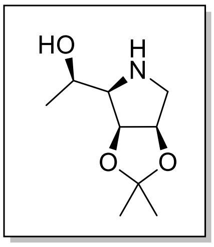

In a manner similar to [2], a 10% NaOH solution (5 mL) was added to a solution of carbamate **21** (289 mg, 1.36 mmol) in EtOH (25 mL) and the reaction mixture was refluxed for 24 h. The solvent was evaporated under reduced pressure and the residue was partitioned between brine (30 mL) and $CHCl_3$ (30 mL). The layers were separated and the aqueous layer was extracted with $CHCl_3$ (30 mL). The combined organic extracts were dried over Na_2SO_4, filtered and the solvent was evaporated under reduced pressure. The

crude product was purified by column chromatography on silica gel (MeOH/$CHCl_3$ 1:20 containing 0.5% (v/v) conc. NH_3) to afford pyrrolidine **22** as white crystals (172 mg, 67%). R_F = 0.13 (MeOH/$CHCl_3$ 1:20 containing 0.5 % (v/v) conc. NH_3); $[\alpha]_D$ = −61.0 (*c* = 0.5; $CHCl_3$); ^{1}H NMR: (400 MHz; $CDCl_3$), δ/ppm: 4.76 (dd, 1H, *J* = 5.6, 4.1 Hz, H-6a), 4.70 (dd, 1H, *J* = 5.6, 3.8 Hz, H-3a), 4.10–4.01 (m, 1H, C*H*OH), 3.16 (d, 1H, *J* = 13.5 Hz, H-6), 2.82 (br s, 1H), 2.66 (dd, 1H, *J* = 13.5, 3.8 Hz, H-6'), 2.56 (dd, 1H, *J* = 4.8, 4.4 Hz, H-4), 2.27 (br s, 1H), 1.48 and 1.32 [2s, each 3H, C(CH_3)$_2$], 1.40 (d, 3H, *J* = 6.6 Hz, CH_3); ^{13}C NMR: (100 MHz; $CDCl_3$), δ/ppm: 110.9 [*C*(CH_3)$_2$], 82.3 (C-3a), 82.2 (C-6a), 68.0 (C-4), 67.4 (*C*HOH), 52.6 (C-6), 25.7 and 23.6 [C(*C*H_3)$_2$], 21.5 (*C*H_3); HRMS (ESI, m/z): calculated for $C_9H_{17}NO_3$ $[M+H]^+$: 188.1281; found 188.1281.

General method for alkylation (Method C)

In a manner similar to [2], K_2CO_3 (1.7 equiv) was added to a stirred solution of pyrrolidine **22** (1 eq.) in anhydrous DMF (5 mL/0.46 mmol of **22**) and the reaction mixture was cooled to 0 °C (ice–water bath). The corresponding bromide was added (1.3 equiv) and the reaction mixture was stirred for 15 min. The ice–water bath was removed and the reaction mixture was stirred at rt overnight. Then, the reaction mixture was partitioned between water (10 mL) and EtOAc (10 mL), the layers were separated and the aqueous layer was extracted with EtOAc (5 mL). The combined organic extracts were washed with water (3 × 10 mL), dried over Na_2SO_4, filtered and the solvent was evaporated under reduced pressure. The crude product was purified by column chromatography.

(*R*)-1-((3a*S*,4*R*,6a*R*)-5-Benzyl-2,2-dimethyltetrahydro-3a*H*-[1,3]dioxolo[4,5-*c*]pyrrol-4-yl)ethanol (23)

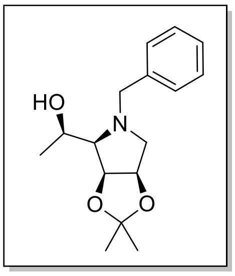

Reaction of **22** (87 mg, 0.47 mmol) with K_2CO_3 (109 mg) and BnBr (72 µL) following the general procedure Method C afforded after column chromatography on silica gel (MeOH/$CHCl_3$ 1:150) pyrrolidine **23** as a white solid (120 mg, 93%). R_F = 0.30 (MeOH/$CHCl_3$ 1:150); $[\alpha]_D$ = −77.0 (*c* = 0.5; $CHCl_3$); ^{1}H NMR: (400 MHz; $CDCl_3$), δ/ppm: 7.35–7.20 (m, 5H, Ar), 4.76 (dd, 1H, *J* = 6.4, 4.2 Hz, H-6a), 4.54 (dd, 1H, *J* = 6.3, 5.1 Hz, H-3a), 4.28–4.18 (m, 2H, C*H*OH, NC*H*$_2$Ar), 3.47 (d, 1H, *J* = 7.5 Hz, OH), 3.11 (d, 1H, *J* = 13.1 Hz, NC*H*$_2$Ar), 3.04 (d, 1H, *J* = 11.0 Hz, H-6), 2.11–2.02 (m, 2H, H-4, H-6'), 1.53 and 1.3 [2s, each 3H, C(CH_3)$_2$], 1.44 (d, 3H, *J* = 6.6 Hz CH_3); ^{13}C NMR: (100 MHz; $CDCl_3$), δ/ppm: 138.0, 128.9, 128.2, 127.0 (Ar), 111.3 [*C*(CH_3)$_2$], 81.2 (C-6a), 77.2 (C-3a), 70.2 (C-4), 64.4 (*C*HOH), 58.8 (C-6), 55.8 (N*C*H_2Ph), 26.2 and 24.8 [C(*C*H_3)$_2$], 20.7 (*C*H_3); HRMS (ESI, m/z): calculated for $C_{16}H_{23}NO_3$ $[M+H]^+$: 278.1751; found 278.1757.

(*R*)-1-((3a*S*,4*R*,6a*R*)-5-(4-Iodobenzyl)-2,2-dimethyltetrahydro-3a*H*-[1,3]dioxolo[4,5-*c*]pyrrol-4-yl)ethanol (24)

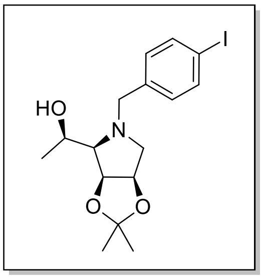

Reaction of **22** (70 mg, 0.37 mmol) with K_2CO_3 (88 mg) and 4-iodobeznyl bromide (144 mg) following the general procedure Method C afforded after column chromatography on silica gel (MeOH/$CHCl_3$ 1:150) pyrrolidine **24** as a white solid (142 mg, 94%). R_F = 0.24 (MeOH/$CHCl_3$ 1:150); $[\alpha]_D$ = −36.7 (*c* = 0.5; $CHCl_3$); ^{1}H NMR: (400 MHz; $CDCl_3$), δ/ppm: 7.63 (d, 2H, *J* = 8.3 Hz, Ar), 7.09 (d, 2H, *J* = 8.2 Hz, Ar), 4.77 (dd, 1H, *J* = 6.4, 4.2 Hz, H-6a), 4.54 (dd, 1H, *J* = 6.3, 5.0 Hz, H-3a), 4.22–4.11 (m, 2H, H-5, NC*H*$_2$Ar), 3.43 (d, 1H, *J* = 8.0 Hz, OH), 3.01 (d, 1H, *J* = 13.3 Hz, NC*H*$_2$Ar), 2.99 (s, 1H, *J* = 10.8 Hz, H-6), 2.06 (t, 1H, *J* = 3.9 Hz, H-4), 2.03 (dd, 1H, *J* = 10.9, 5.0 Hz, H-6'),

1.52 and 1.30 [2s, each 3H, C(C*H*$_3$)$_2$], 1.43 (d, 3H, *J* = 6.6 Hz, C*H*$_3$); ^{13}C NMR: (100 MHz; $CDCl_3$), δ/ppm: 138.1, 137.4, 130.8 (Ar), 111.3 [*C*(CH$_3$)$_2$], 92.3 (Ar), 81.1 (C-6a), 77.2 (C-3a), 70.3 (C-4), 64.4 (*C*HOH), 58.9 (C-6), 55.3 (N*C*H$_2$Ar), 26.2 and 24.7 [C(*C*H$_3$)$_2$], 20.8 (*C*H$_3$); HRMS (ESI, m/z): calculated for $C_{16}H_{22}INO_3$ [*M*+H]$^+$: 404.0717; found 404.0714.

(*R*)-1-((3a*S*,4*R*,6a*R*)-2,2-Dimethyl-5-(naphthalen-2-ylmethyl)tetrahydro-3a*H*-[1,3]dioxolo[4,5-*c*]pyrrol-4-yl)ethanol (25)

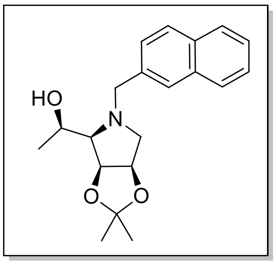

Reaction of **22** (58 mg, 0.31 mmol) with K_2CO_3 (73 mg) and 2-(bromomethyl)naphthalene (89 mg) following the general procedure Method C afforded after column chromatography on silica gel (EtOAc/hexane 8:1→4:1) pyrrolidine **25** as a yellow oil (91 mg, 90%). R_F = 0.22 (MeOH/CHCl$_3$ 1:150); $[\alpha]_D$ = −56.3 (*c* 0.23; CHCl$_3$); ^{1}H NMR: (400 MHz; CD_3OD), δ/ppm: 7.86-7.81 (m, 4H, Ar), 7.56 (dd, 1H, *J* = 8.6, 1.4 Hz, Ar), 7.49-7.44 (m, 2H, Ar), 4.84 (m, 1H, H-3a), 4.62 (dd, 1H, *J* = 6.3, 5.0 Hz, H-6a), 4.46 (d, 1H, *J* = 13.2 Hz, NC*H*$_2$Ar), 4.33 (qd, 1H, *J* = 6.5, 3.3 Hz, C*H*OH), 3.46 (d, 1H, *J* = 13.7 Hz, NC*H*$_2$Ar), 3.05 (d, 1H, *J* = 11.4 Hz, H-6), 2.49 (s, 1H, H-4), 2.37 (d, 1H, *J* = 6.9 Hz, H-6‘), 1.52 and 1.29 [2s, each 3H, C(C*H*$_3$)$_2$], 1.48 (d, 3H, *J* = 6.6 Hz, C*H*$_3$); ^{13}C NMR: (100 MHz; CD_3OD), δ/ppm: 134.8, 134.5, 129.1, 128.8, 128.7, 128.5, 127.2, 127.0 (10C, Ar), 112.5 (*C*(CH$_3$)$_2$), 82.1 (C-3a), 78.4 (C-6a), 72.2 (C-4), 66.0 (*C*HOH), 59.6 (C-6), 57.8 (N*C*H$_2$Ar), 26.0 and 24.4 [C(*C*H$_3$)$_2$], 20.5 (*C*H$_3$); HRMS (ESI, m/z): calculated for $C_{20}H_{25}NO_3$ [*M*+H]$^+$: 328.1907; found 328.1918.

(2*R*,3*S*,4*R*)-1-Benzyl-2-((*R*)-1-hydroxyethyl)pyrrolidine-3,4-diol (26)

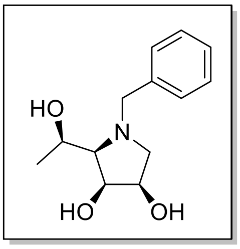

Deprotection of **23** (116 mg, 0.31 mmol) following general procedure Method C afforded after purification by column chromatography on silica gel (MeOH/CHCl$_3$ 1:20 containing 0.5% (v/v) conc. NH$_3$) triol **26** as a yellowish oil (67 mg, 67%). R_F = 0.11 (MeOH/CHCl$_3$ 1:20 containing 0.5 % (v/v) conc. NH$_3$); $[\alpha]_D$ = −92.3 (*c* = 0.5; CHCl$_3$); ^{1}H NMR: (400 MHz; $CDCl_3$), δ/ppm: 7.36–7.23 (m, 5H, Ar), 4.37 (t, 1H, *J* = 5.7 Hz, H-3), 4.13–4.03 (m, 2H, H-4, C*H*OH), 3.94 (d, 1H, *J* = 13.5 Hz, NC*H*$_2$Ar), 3.42 (d, 1H, *J* = 13.5 Hz, NC*H*$_2$Ar), 3.01 (dd, 1H, *J* = 11.0, 3.9 Hz, H-5), 2.62–2.55 (m, 2H, H-2, H-5'), 1.43 (d, 3H, *J* = 6.7 Hz, C*H*$_3$); ^{13}C NMR: (100 MHz; $CDCl_3$), δ/ppm: 138.4, 128.6, 128.5, 127.3 (Ar), 73.6 (C-3), 70.5 (C-4), 69.1 (C-2), 66.8 (*C*HOH), 58.8 (N*C*H$_2$Ar), 58.5 (C-5), 18.2 (*C*H$_3$); HRMS (ESI, m/z): calculated for $C_{13}H_{19}NO_3$[*M*+H]$^+$: 238.1437; found 238.1432.

(2*R*,3*S*,4*R*)-2-((*R*)-1-Hydroxyethyl)-1-(4-iodobenzyl)pyrrolidine-3,4-diol (27)

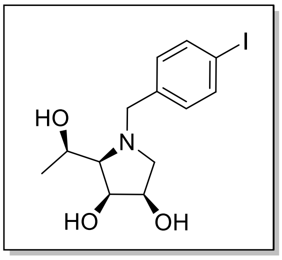

Deprotection of **24** (140 mg, 0.35 mmol) following general procedure Method C afforded after purification by column chromatography on silica gel (MeOH/CHCl$_3$ 1:20 containing 0.5% (v/v) conc. NH$_3$) triol **27** as an orange solid (116 mg, 92%). R_F = 0.17 (MeOH/CHCl$_3$ 1:20 containing 0.5% (v/v) conc. NH$_3$); $[\alpha]_D$ = −50.9 (*c* = 0.5; CHCl$_3$); ^{1}H NMR: (400 MHz; $CDCl_3$), δ/ppm: 7.65 (d, 2H, *J* = 8.3 Hz, Ar), 7.05 (d, 2H, *J* = 8.2 Hz, Ar), 4.38 (t, 1H, *J* = 5,6 Hz, H-3), 4.24 (br s, 1H, OH), 4.14–4.01 (m, 2H, H-4, C*H*OH), 3.89 (d, 1H, *J* = 13.7 Hz, NC*H*$_2$Ar), 3.34 (d, 1H, *J* = 13.7 Hz, NC*H*$_2$Ar), 2.98 (dd, 1H, *J* = 10.9, 3.7 Hz, H-5), 2.85 (br s, 1H, OH), 2.58 (dd, 1H, *J* = 6.4, 2.9 Hz, H-2), 2.51 (dd, 1H, *J* = 10.9, 5.8 Hz, H-5'),

1.43 (d, 3H, J = 6.7 Hz, CH_3); ^{13}C NMR: (100 MHz; $CDCl_3$), δ/ppm: 138.1, 137.7, 130.4, 92.6 (Ar), 73.6 (C-3), 70.5 (C-4), 69.1 (C2), 66.9 (*C*HOH), 58.7 (N*C*H_2Ar), 58.0 (C-5), 18.3 (*C*H_3); HRMS (ESI, m/z): calculated for $C_{13}H_{18}INO_3$ [*M*+H]$^+$: 364.0404; found 364.0399.

(2*R*,3*S*,4*R*)-2-((*R*)-1-Hydroxyethyl)-1-(naphthalen-2-ylmethyl)pyrrolidine-3,4-diol (28)

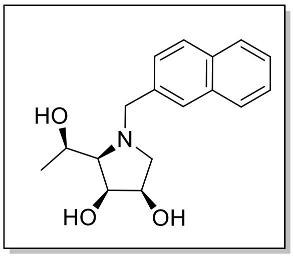

Deprotection of **25** (80 mg, 0.24 mmol) following general procedure Method C afforded after purification by column chromatography on silica gel ($CHCl_3$/MeOH 20:1 containing 0.5% (v/v) conc. NH_3) triol **28** as a yellow oil (40 mg, 57%). R_F = 0.10 (MeOH/$CHCl_3$ 1:20 containing 0.5 % (v/v) conc. NH_3); $[\alpha]_D$ = −31.6 (*c* 0.2; CH_3OH); ^{1}H NMR: (400 MHz; CD_3OD), δ/ppm: 7.87-7.84 (m, 4H, Ar), 7.57 (dd, 1H, J = 8.6, 1.4 Hz, Ar), 7.50-7.47 (m, 2H, Ar), 4.40 (dd, 1H, J = 6.1, 4.8 Hz, H-3), 4.27 (d, 1H, J = 13.3 Hz, NCH_2Ar), 4.17-4.09 (m, 2H, H-4, C*H*OH), 3.76 (d, 1H, J = 13.3 Hz, NCH_2Ar), 3.01 (dd, 1H, J = 10.9, 4.7 Hz, H-5), 2.86 (dd, 1H, J = 6.1, 4.8 Hz, H-2), 2.77 (dd, 1H, J = 10.9, 6.0 Hz, H-5'), 1.39 (d, 3H, J = 6.6 Hz, CH_3); ^{13}C NMR: (100 MHz; CD_3OD), δ/ppm: 136.4, 134.8, 134.4, 129.1, 129.0, 128.8, 128.6, 128.3, 127.2, 127.0 (Ar), 74.5 (C-3), 71.5 (C-4), 71.2 (C-2), 68.4 (*C*HOH), 60.8 (N*C*H_2Ar), 58.6 (C-5), 20.5 (*C*H_3); HRMS (ESI, *m/z*): calculated for $C_{17}H_{21}NO_3$ [*M*+H]$^+$: 288.1594; found 288.1593.

(2*R*,3*S*,4*R*)-2-((*R*)-1-Hydroxyethyl)pyrrolidine-3,4-diol (29)

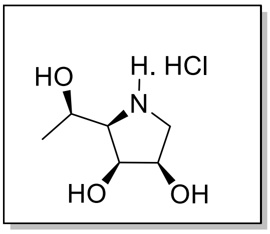

A suspension of 10% Pd-C (7.5 mg, 20 wt %) and pyrrolidine **26** (37 mg, 0.16 mmol) in MeOH (5 mL) was stirred at rt under a hydrogen atmosphere (balloon) for 5 h. The catalyst was filtered off, the filtrate was cooled to 0 °C (ice–water bath) and acidified with conc. HCl (50 µL). The ice–water bath was removed and the solvents were evaporated. The product was lyophilized to give **29** as a yellowish solid (25 mg, 86%). R_F = 0.05 (MeOH/EtOAc 1:3 containing 0.5% (v/v) conc. NH_3); $[\alpha]_D$ = +83.7 (*c* 0.42; H_2O); ^{1}H NMR: (400 MHz; D_2O), δ/ppm: 4.55 (dt, 1H, J = 8.5, 3.9 Hz, H-4), 4.43 (t, 1H, J = 3.4 Hz, H-3), 4.25 (dq, 1H, J = 8.4, 6.4 Hz C*H*OH), 3.64 (dd, 1H, J = 12.0, 8.4 Hz, H-5), 3.47 (dd, 1H, J = 8.4, 3.0 Hz, H-2), 3.22 (dd, 1H, J = 12.0, 8.6 Hz, H-5'), 1.36 (d, 3H, J = 6.4 Hz, CH_3); ^{13}C NMR: (100 MHz; D_2O), δ/ppm: 70.0 (C-4), 69.5 (C-3), 65.8 (C-2), 62.9 (*C*HOH), 47.0 (C-5), 19.3 (*C*H_3); HRMS (ESI, m/z): calculated for $C_6H_{13}NO_3$ [*M*+H]$^+$: 148.0968; found 148.0975.

Enzyme assay

Recombinant soluble forms of *Drosophila melanogaster* Golgi (GMIIb) and lysosomal (LManII) α-mannosidases as well as *Caenorhabditis elegans* Golgi α-mannosidase AMAN-2 were produced in *Pichia pastoris* and enriched by ammonium sulfate precipitation or nickel chelation chromatography as previously described [3,4]. The α-mannosidase from *Canavalia ensiformis* (JBMan) was purchased from Sigma. The mannosidase activity of these enzyme preparations were measured using *p*-nitrophenyl α-D-mannopyranoside (*p*NP-Man; Sigma; 100 mM stock in dimethylsulfoxide) as a substrate at 2 mM final concentration in 50 mM acetate buffer of the relevant previously defined optimal pH, GMIIb and AMAN-2 at pH 6.0, LManII at pH 5.2, and JBMan at pH 5.0) and 0.5 µL of the enzyme (0.05 µg of protein for JBMan), in a total volume of 50 µL for 1–2 h at 37 °C. GMIIb was assayed in the presence of 0.5 mM $CoCl_2$.

The lyophilized derivatives used in the assay were dissolved in DMSO to the final concentration 50 mM and further diluted to a desired concentration in water. These derivatives were preincubated with the enzyme in the buffer for 5 min at rt and the reaction was started by addition of the substrate. The reactions were terminated with two volumes (0.1 mL) of 0.5 M sodium carbonate and the production of *p*-nitrophenol was measured at 405 nm using a multimode reader Mithras LB943 (Berthold Technologies). The average or representative result of three independent experiments made in duplicate is presented. The IC_{50} values were determined with 2 mM *p*NP-Man. The K_i values were determined from Dixon plots of assays performed with *p*NP-Man (0.5-4 mM).

Molecular modeling

Docking with Glide. The X-ray structure of recombinant *Drosophila melanogaster* Golgi α-mannosidase II (dGMII, PDB ID: 3BLB) [5,6] was used as 3D enzyme models of human GMII and LMan for docking of the synthesized compounds with the GLIDE program [7,8] of the Schrödinger package. Protonation states of amino acid residues of the enzymes was calculated for the pH 6.0 (dGMII) using the Propka v.2 program [9,10]. For docking with dGMII all crystallographic molecules of water at the active site of dGMII were deleted except one (WAT1820, numbering according to PDB ID: 3BLB). This water has been shown to be conserved in crystal structures of dGMII either with intact substrates or inhibitors [11-13]. In docking calculations, the catalytic acid (Asp341 of dGMII) was modeled either in the neutral (as Ash^0) or ionized (as Asp^-) form to see differences in prediction of binding poses of the docked ligands. The receptor box for the docking conformational search was centered at the Zn^{2+} ion cofactor at the bottom of the active site with a size of 39 × 39 × 39 Å using partial atomic charges for the receptor from the OPLS2005 force field except for the Zn^{2+} and side chains of His90, Asp92, Asp204, Arg228, Tyr269, Asp270, Asp340, Asp341 and His471. For these structural fragments the charges were calculated at the quantum mechanics level with the DFT (Density Functional Theory) method (M06-2X) [13] using a hybrid QM/MM model (M06-2X/LACVP**:OPLS2005) [15-17] with the QSite [18,19] program of the Schrödinger package. The grid maps were created with no Van der Waals radius and charge scaling for the atoms of the receptor. Flexible docking in standard (SP) precision was used. The partial atomic charges of the docked ligands were calculated at the DFT level (M06-2X/LACVP**) [14,15] using the Jaguar program [20] of the Schrödinger package. All ligands were docked with the amino group at the pyrrolidine ring either in protonated and neutral forms. The potential for nonpolar parts of the ligands was softened by scaling the van der Waals radii by a factor of 0.8 for atoms of the ligands with partial atomic charges less than specified cut-off of 0.15. The 5000 poses were kept per ligand for the initial docking stage with scoring window of 100 kcal mol^{-1} for keeping initial poses; the best 400 poses were kept per ligand for energy minimization. The ligand poses with RMS deviations less than 0.5 Å and maximum atomic displacement less than 1.3 Å were discarded as duplicates. The post-docking minimization for 10 ligand poses with the best docking score was performed and optimized structures were saved for subsequent analyses using the MAESTRO viewer [21] of the Schrödinger package.

QM/MM geometry optimizations. Geometries of selected complexes (inhibitor:enzyme) from molecular docking were subsequently optimized at the QM/MM level (BP86/LACVP*:OPLS2005) [15,17,22] using the Qsite [18,19] program of the Schrödinger package. The following decomposed scheme was used: the QM part (more than 280 atoms) of the inhibitor:dGMII system consisted of Zn^{2+} ion, inhibitor, water molecule WAT1820 (described in the previous section) and amino acid residues (Asp92, Asp204, Asp341, Asp340, Asp270, Asp409, Asp472, Arg228, Arg876, His90, His471, Tyr267, Tyr269, Tyr727, Ser268, Trp95, Trp415, Phe206). The rest of the enzyme was included into the MM part and described by the OPLS2005 force field [17]. The QM/MM methodology (an additive scheme) with hydrogen caps on boundary QM atoms and electrostatic treatment at the interface between the QM and MM regions using Gaussian charge distributions represented on a grid (keyword HCAPESCHG=3) was employed. The neutral form of the pyrrolidine ring of the inhibitors and the ionized form of the catalytic acid Asp341 of dGMII were considered. These ionized forms were used based on previous pK_a calculations of imino-D-lyxitols bound at the active site of dGMII [32].

FMO-PIEDA calculations. Pair interaction energy decomposition analysis (PIEDA) was used along the two-body FMO method [23-25]. From the QM/MM-optimized inhibitor:enzyme complexes active-site clusters

consisted of more than 30 amino acid residues, Zn^{2+} ion and the bound inhibitor were built using the Facio program [26]. The hybrid orbital projection operator (HOP) technique was used in the generation of fragments for the covalently bounded amino acids. The FMO calculations were performed using the second-order Møller-Plesset theory [27,28] (MP2) with the 6-31G(d) basis and polarizable continuum model (PCM) [29]. The Gamess package [30,31] [version 30 June 2021 (R1)] was used. The virtue of the FMO technique is to predict pair interactions between the two structural fragments of the molecular system embedded within the electrostatic potential of the surroundings (IFIE – inter fragment interaction energy). FMO-PIEDA enables the separation of the interaction energy into physically interpretable

$$E_{\mathrm{int}} = E_{\mathrm{els}} + E_{\mathrm{exch}} + E_{\mathrm{ct\text{-}mix}} + E_{\mathrm{disp}}, \qquad (1)$$

The electrostatic energy E_{els} originates from Coulomb-like interactions between the fragments, the exchange energy E_{exch} arises for fermion particles, the electrons, and accounts for the Pauli repulsion of electrons between the fragments. $E_{\mathrm{ct+mix}}$ is somewhat peculiar; it includes the charge transfer that results from electron transfer from occupied molecular orbitals of one fragment to the vacant virtual orbitals on the second fragment. The mixing part is basically an approximate polarization. Dispersion energy E_{disp} originates from interactions of instantaneous fluctuations of dipoles on the fragments due to electron correlation. This method was recently used to analyze interaction energy in different biomolecular systems [32-37].

To understand an inhibitory effect of a substituent at C-5 of the inhibitor ring of **10**, **20**, **28**, **29**, **30**, **31** and DIM, the inhibitor was divided into two fragments, the pyrrolidine ring structure (I_{ring}) and the methyl moiety (I_{linker}) (in **10**), (1*S*)-1-hydroxyethyl (in **20**), (1*R*)-1-hydroxyethyl (in **28** and **29**), hydroxymethyl (in **30** and **31**) and (1*R*)-1,2-dihydroxyethyl moiety in (DIM). Then, the interaction energy between the inhibitor and enzyme ($\Delta E_{\mathrm{I\text{-}E}}$) consists of the interaction energy between the pyrrolidine ring of the inhibitor and enzyme ($\Delta E_{\mathrm{ring\text{-}E}}$) and the interaction energy between the linker of the inhibitor and enzyme ($\Delta E_{\mathrm{linker\text{-}E}}$):

$$\Delta E_{\mathrm{I\text{-}E}} = \Delta E_{\mathrm{ring\text{-}E}} + \Delta E_{\mathrm{linker\text{-}E}} \qquad (2)$$

References

1. An, S.; Kim, G.; Kim, H.J.; Ahn, S.; Kim, H.Y.; Ko, H.; Hyun, Y.E.; Nguyen, M.; Jeong, J.; Liu, Z.; Han, J.; Choi, H.; Yu, J.; Kim, J.W.; Lee, H.W.; Jacobson, K.A.; Cho, W. J.; Kim, Y.M.; Kang, K.W.; Noh, M.; Jeong, L S. *J. Med. Chem.* **2020**, *63*, 16012. DOI: 10.1021/acs.jmedchem.0c01874
2. Bella, M.; Šesták, S.; Moncoľ, J.; Koóš, M.: Poláková, M. *Beilstein J. Org. Chem.* **2018**, *14*, 2156. DOI: 10.3762/bjoc.14.189
3. Nemčovičová, I.; Šesták, S.; Rendić, D.; Plšková, M.; Mucha J.; Wilson, I.B.H. *Glycoconj. J.* **2013**, *30*, 899. DOI: 10.1007/s10719-013-9495-5
4. Paschinger, K.; Hackl, M.; Gutternigg, M.; Kretschmer-Lubich, D.; Stemmer, U.; Jantsch, V.; Lochnit, G.; Wilson, I.B.H. *J. Biol. Chem.* **2006**, *281*, 28265. DOI: 10.1074/jbc.M602878200
5. van den Elsen, J. M. H.; Kuntz, D. A.; Rose, D. R. *EMBO J.* **2001**, *20*, 3008. DOI: 10.1093/emboj/20.12.3008
6. Kuntz, D. A.; Rose, D. R. (2007) Golgi Mannosidase II in complex with swainsonine at 1.3 Angstrom doi: 10.2210/pdb3BLB/pdb
7. Friesner, R. A.; Banks, J. L.; Murphy, R. B.; Halgren, T. A.; Klicic, J. J.; Mainz, D. T.; Repasky, M. P.; Knoll, E. H.; Shelley, M.; Perry, J. K.; Shaw, D. E.; Francis, P.; Shenkin, P. S. *J. Med. Chem.* **2004**, *47*, 1739. DOI: 10.1021/jm0306430
8. Glide, version 7.0, Schrödinger, LLC: New York, NY, **2016**.
9. Li, H.; Robertson, A. D.; Jensen, J. H. *Proteins: Struct., Funct., Bioinf.* **2005**, *61*, 704. DOI: 10.1002/prot.20660
10. Bas, D. C.; Rogers, D. M.; Jensen, J. H. *Proteins: Struct., Funct., Bioinf.* **2008**, *73*, 765. DOI: 10.1002/prot.22102
11. Kuntz, D. A.; Nakayama, S.; Shea, K.; Hori, H.; Uto, Y.; Nagasawa, H.; Rose, D. R. *ChemBioChem* **2010**, *11*, 673. DOI: 10.1002/cbic.200900750
12. Kuntz, D. A.; Zhong, W.; Guo, J.; Rose, D. R.; Boons, G. J. *ChemBioChem* **2009**, *10*, 268. DOI: 10.1002/cbic.200800538
13. Shah, N.; Kuntz, D. A.; Rose, D. R. *Proc. Natl. Acad. Sci. U.S.A.* **2008**, *105* (28), 9570. DOI: 10.1073/pnas.0802206105
14. Zhao, Y.; Truhlar, D. G. *Theor. Chem. Acc.* **2008**, *120*, 215. DOI: 10.1007/s00214-007-0310-x
15. Wadt, W. R.; Hay, P. J. *J. Chem. Phys.* **1985**, *82*, 284. DOI: 10.1063/1.448800
16. Hay, P. J.; Wadt, W. R. *J. Chem. Phys.* **1985**, *82*, 270. DOI: 10.1063/1.448799
17. Kaminski, G. A.; Friesner, R. A.; Tirado-Rives, J.; Jorgensen, W. L. *J. Phys. Chem. B* **2001**, *105*, 6474. DOI: 10.1021/jp003919d
18. Murphy, R. B.; Philipp, D. M.; Friesner, R. A. *J. Comput. Chem.* **2000**, *21*, 1442. DOI: 10.1002/1096-987X(200012)21:16<1442::AID-JCC3>3.0.CO;2-O
19. Qsite; Schrödinger, LLC: New York, NY, 2016.
20. Jaguar, version 9.1, Schrödinger, LLC: New York, NY, 2016.
21. Schrödinger Suite 2016 Protein Preparation Wizard; Epik v. 3.5; Impact v. 7.0, Schrödinger; Maestro v.10.5, LLC: New York, NY, **2016**.
22. Becke, A. D. *Phys. Rev. A* **1988**, *38*, 3098. DOI: 10.1103/PhysRevA.38.3098
23. Kitaura, K.; Ikeo, E.; Asada, T.; Nakano, T.; Uebayasi, M. *Chem. Phys. Lett.* **1999**, *313*, 701. DOI: 10.1016/S0009-2614(99)00874-X
24. Fedorov, D. G.; Kitaura, K. In *The Fragnemt Molecular Orbital Method - Practical Applications to Large Molecular Systems*, Fedorov, D. G.; Kitaura, K., Eds. CRC Press, Taylor and Francis Group: **2009**; pp 5-36.
25. Fedorov, D. G.; Nagata, T.; Kitaura, K. *Phys. Chem. Chem. Phys* **2012**, *14*, 7562. DOI: 10.1039/C2CP23784A
26. Suenaga, M. Development of GUI for GAMESS / FMO Calculation. *J. Comput. Chem.- Japan* **2008**, *7*, 33. DOI: 10.2477/jccj.H1920
27. Møller, C.; Plesset, M. S. *Phys. Rev.* **1934**, *46*, 618. DOI: 10.1103/PhysRev.46.618
28. Frisch, M. J.; Headgordon, M.; Pople, J. A. *Chem. Phys. Lett.* **1990,** *166*, 275. DOI: 10.1016/0009-2614(90)80029-D
29. Fedorov, D. G.; Kitaura, K.; Li, H.; Jensen, J. H.; Gordon, M. S. *J. Comput. Chem.* **2006**, *27*, 976. DOI: 10.1002/jcc.20406
30. Barca, G. M. J.; Bertoni, C.; Carrington, L.; Datta, D.; De Silva, N.; Deustua, J. E.; Fedorov, D. G.; Gour, J. R.; Gunina, A. O.; Guidez, E.; Harville, T.; Irle, S.; Ivanic, J.; Kowalski, K.; Leang, S. S.; Li, H.; Li, W.; Lutz, J. J.; Magoulas, I.; Mato, J.; Mironov, V.; Nakata, H.; Pham, B. Q.; Piecuch, P.; Poole, D.; Pruitt, S. R.; Rendell, A. P.; Roskop, L. B.; Ruedenberg, K.; Sattasathuchana, T.; Schmidt, M. W.; Shen, J.; Slipchenko, L.; Sosonkina, M.; Sundriyal, V.; Tiwari, A.; Vallejo, J. L. G.; Westheimer, B.; Wloch, M.; Xu, P.; Zahariev, F.; Gordon, M. S. *J. Chem. Phys.* **2020**, *152*, 154102. DOI: 10.1063/5.0005188
31. Schmidt, M. W.; Baldridge, K. K.; Boatz, J. A.; Elbert, S. T.; Gordon, M. S.; Jensen, J. H.; Koseki, S.; Matsunaga, N.; Nguyen, K. A.; Su, S. J.; Windus, T. L.; Dupuis, M.; Montgomery, J. A. *J. Comput. Chem.* **1993**, *14*, 1347. DOI: 10.1002/jcc.540141112

32. Kóňa, J.; Šesták, S.; Wilson, I.B.H.; Poláková, M. *Org. Biomol. Chem.* **2022**, *20,* 8932. DOI: 10.1039/D2OB01545E
33. Lim, H.; Chun, J.; Jin, X.; Kim, J.; Yoon, J.; No, K. T. *Sci. Rep.* **2019**, *9,* 16727. DOI: 10.1038/s41598-019-53216-z
34. Sogawa, H.; Sato, R.; Suzuki, K.; Tomioka, S.; Shinzato, T.; Karpov, P.; Shulga, S.; Blume, Y.; Kurita, N. *Chem. Phys.* **2020**, *530*, 110603. DOI: 10.1016/j.chemphys.2019.110603
35. Anan, R.; Nakamura, T.; Shimamura, K.; Matsushita, Y.; Ohyama, T.; Kurita, N. *J. Mol. Model.* **2019**, *25*, 192. DOI: 10.1007/s00894-019-4087-3
36. Sladek, V.; Kóňa, J.; Tokiwa, H. *Phys. Chem. Chem. Phys* **2017**, *19*, 12527. DOI: 10.1039/C7CP01200D
37. Takaya, D.; Niwa, H.; Mikuni, J.; Nakamura, K.; Handa, N.; Tanaka, A.; Yokoyama, S.; Honma, T. *J. Mol. Graph. Model.* **2020,** *99*, 107599. DOI: 10.1016/j.jmgm.2020.107599
