# Supplementary material for: Synthesis, α-mannosidase inhibition studies and molecular modeling of 1,4-imino-ᴅ-lyxitols and their C-5-altered N-arylalkyl derivatives
Source: Beilstein J Org Chem. 2023 Mar 6;19:282–93. doi: 10.3762/bjoc.19.24 (PMC10012049; doi:10.3762/bjoc.19.24)
Supplement: File 2 — Copies of NMR spectra. [file Beilstein_J_Org_Chem-19-282-s002.pdf]

BEILSTEIN JOURNAL OF ORGANIC CHEMISTRY

Supporting Information

for

Synthesis, α-mannosidase inhibition studies and molecular modeling of 1,4-imino-D-lyxitols and their C-5-altered *N*-arylalkyl derivatives

Martin Kalník, Sergej Šesták, Juraj Kóňa, Maroš Bella and Monika Poláková

Beilstein J. Org. Chem. **2023,** *19,* 282–293. doi:10.3762/bjoc.19.24

Copies of NMR spectra

License and Terms: This is a supporting information file under the terms of the Creative Commons Attribution License (https://creativecommons.org/licenses/by/4.0). Please note that the reuse, redistribution and reproduction in particular requires that the author(s) and source are credited and that individual graphics may be subject to special legal provisions.
The license is subject to the *Beilstein Journal of Organic Chemistry* terms and conditions: (https://www.beilstein-journals.org/bjoc/terms)

Table of contents

^{1}H and ^{13}C NMR spectra of the target compounds S2–S13

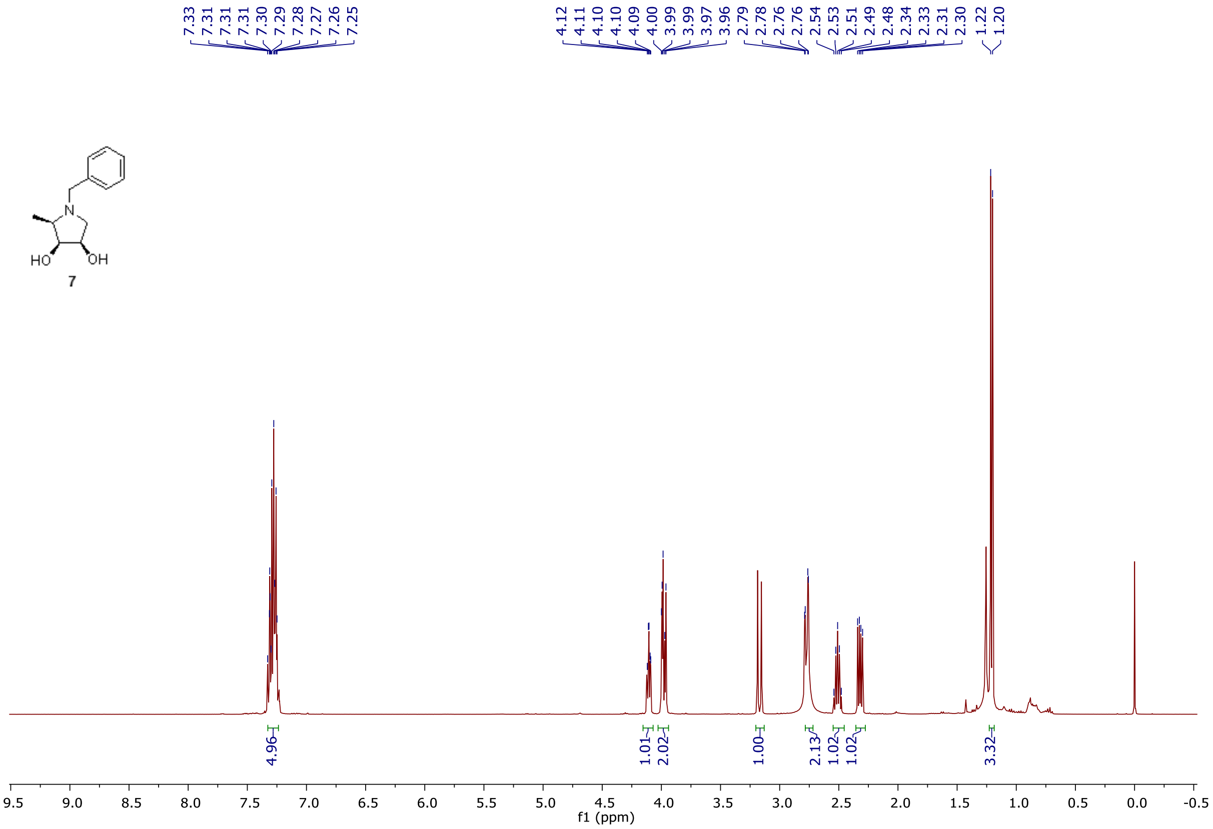
7.33 7.31 7.31 7.30 7.29 7.28 7.27 7.26 7.25
4.12 4.11 4.10 4.10 4.09 4.00 3.99 3.99 3.97 3.96 2.79 2.78 2.76 2.76 2.54 2.53 2.51 2.49 2.48 2.34 2.33 2.31 2.30 1.22 1.20
HO
OH
N
7
4.96
1.01
2.02
1.00
2.13
1.02
1.02
3.32
9.5 9.0 8.5 8.0 7.5 7.0 6.5 6.0 5.5 5.0 4.5 4.0 3.5 3.0 2.5 2.0 1.5 1.0 0.5 0.0 -0.5
f1 (ppm)

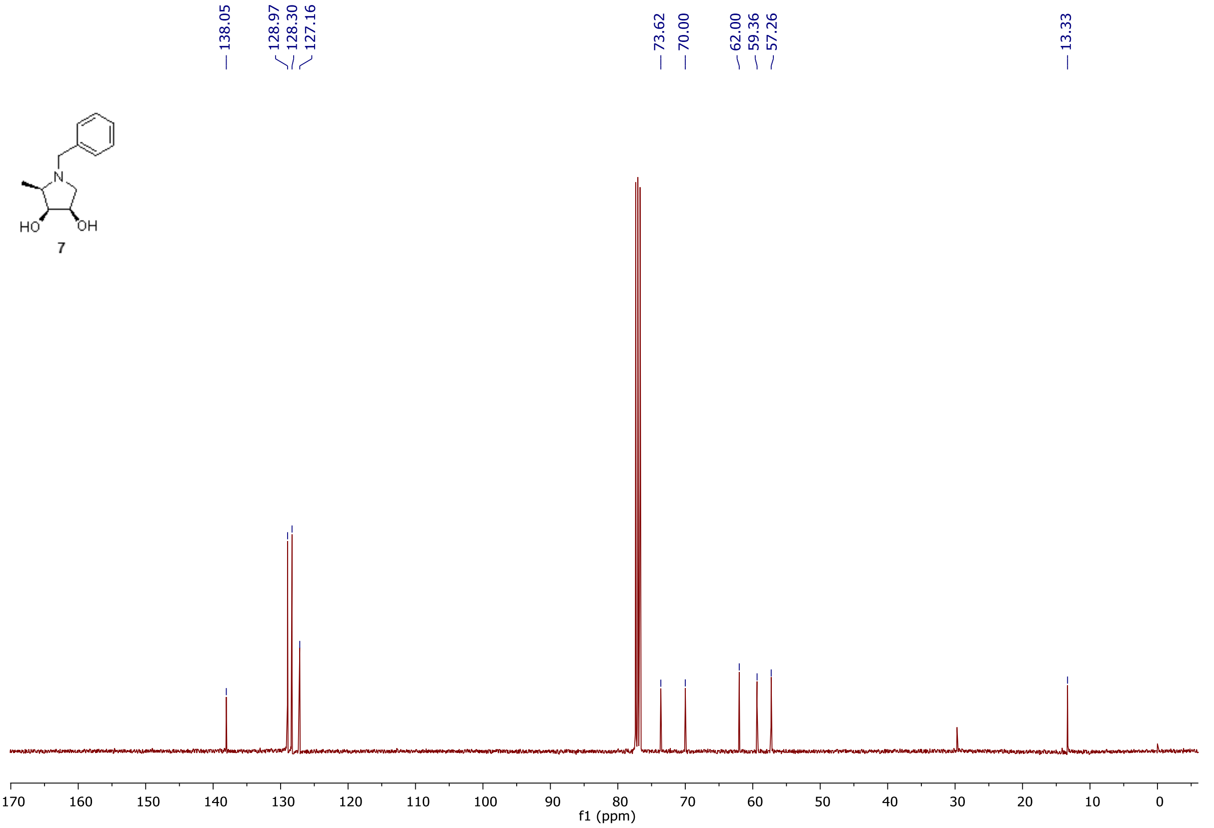
138.05
128.97 128.30 127.16
73.62
70.00
62.00 59.36 57.26
13.33
HO
OH
N
7
170 160 150 140 130 120 110 100 90 80 70 60 50 40 30 20 10 0
f1 (ppm)

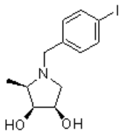

8

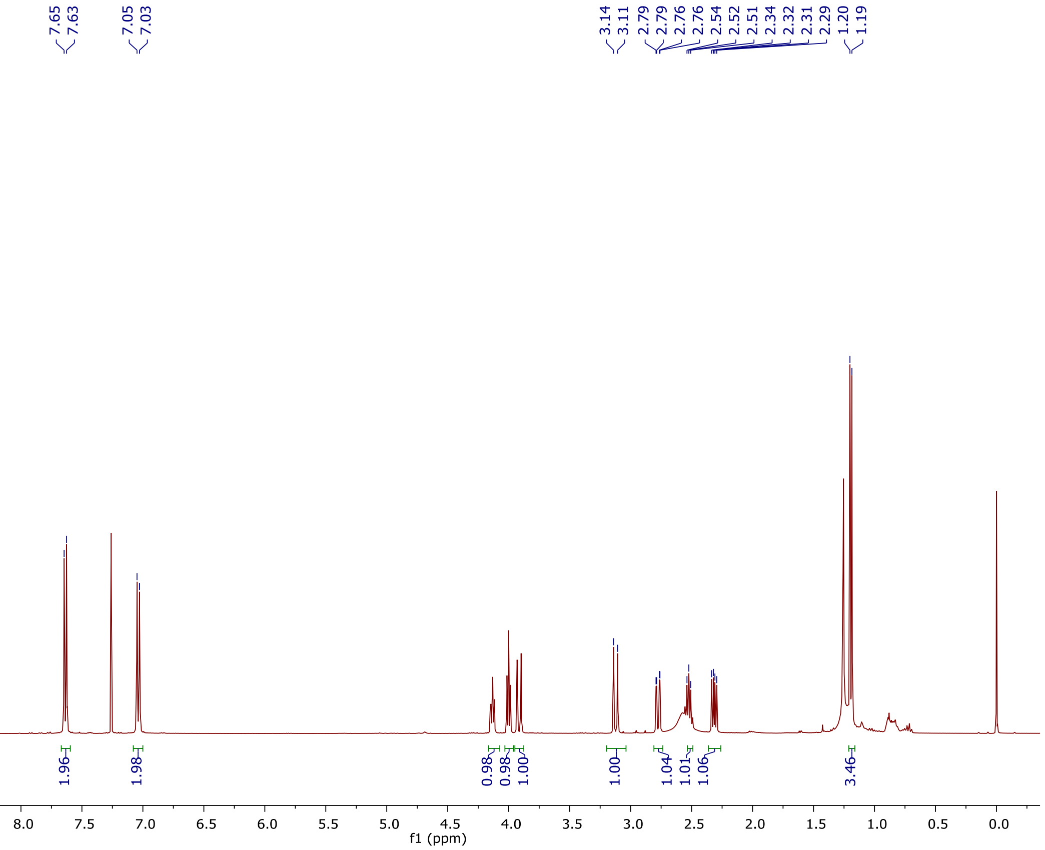

7.65
7.63
7.05
7.03
3.14
3.11
2.79
2.79
2.76
2.76
2.54
2.52
2.51
2.34
2.32
2.31
2.29
1.20
1.19
1.96
1.98
0.98
0.98
1.00
1.00
1.04
1.01
1.06
3.46
9.5 9.0 8.5 8.0 7.5 7.0 6.5 6.0 5.5 5.0 4.5 4.0 3.5 3.0 2.5 2.0 1.5 1.0 0.5 0.0
f1 (ppm)

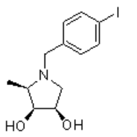

8

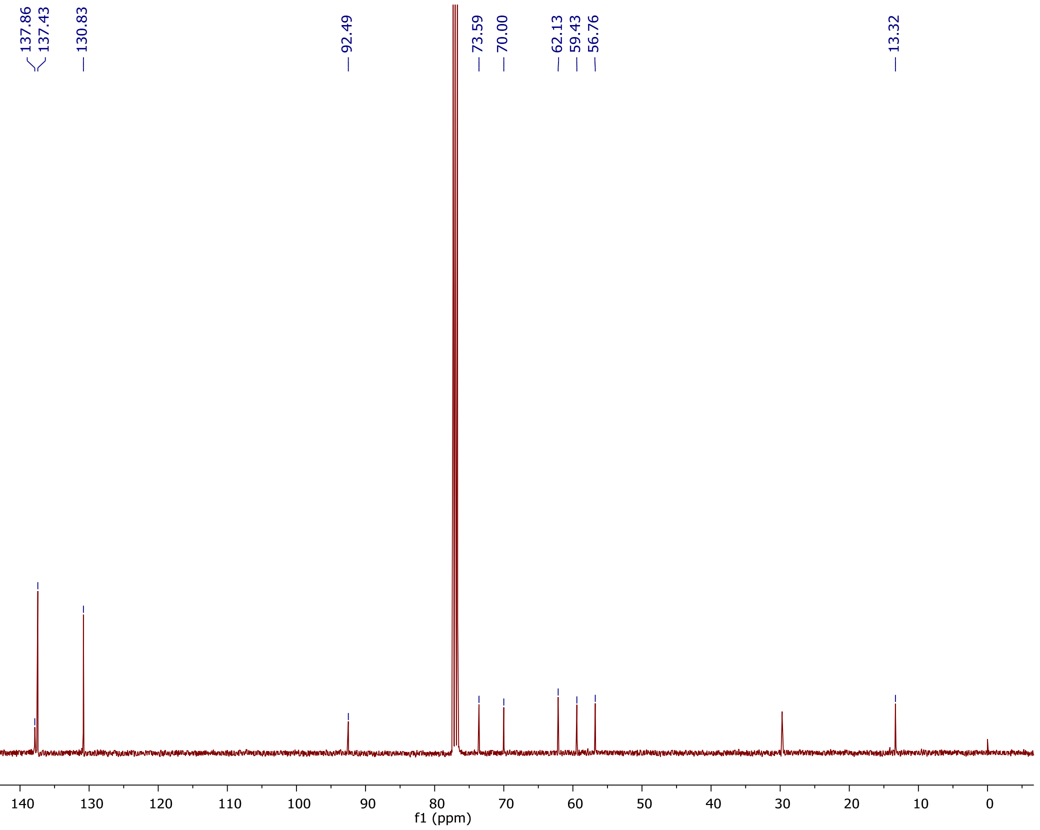

137.86
137.43
130.83
92.49
73.59
70.00
62.13
59.43
56.76
13.32
160 150 140 130 120 110 100 90 80 70 60 50 40 30 20 10 0
f1 (ppm)

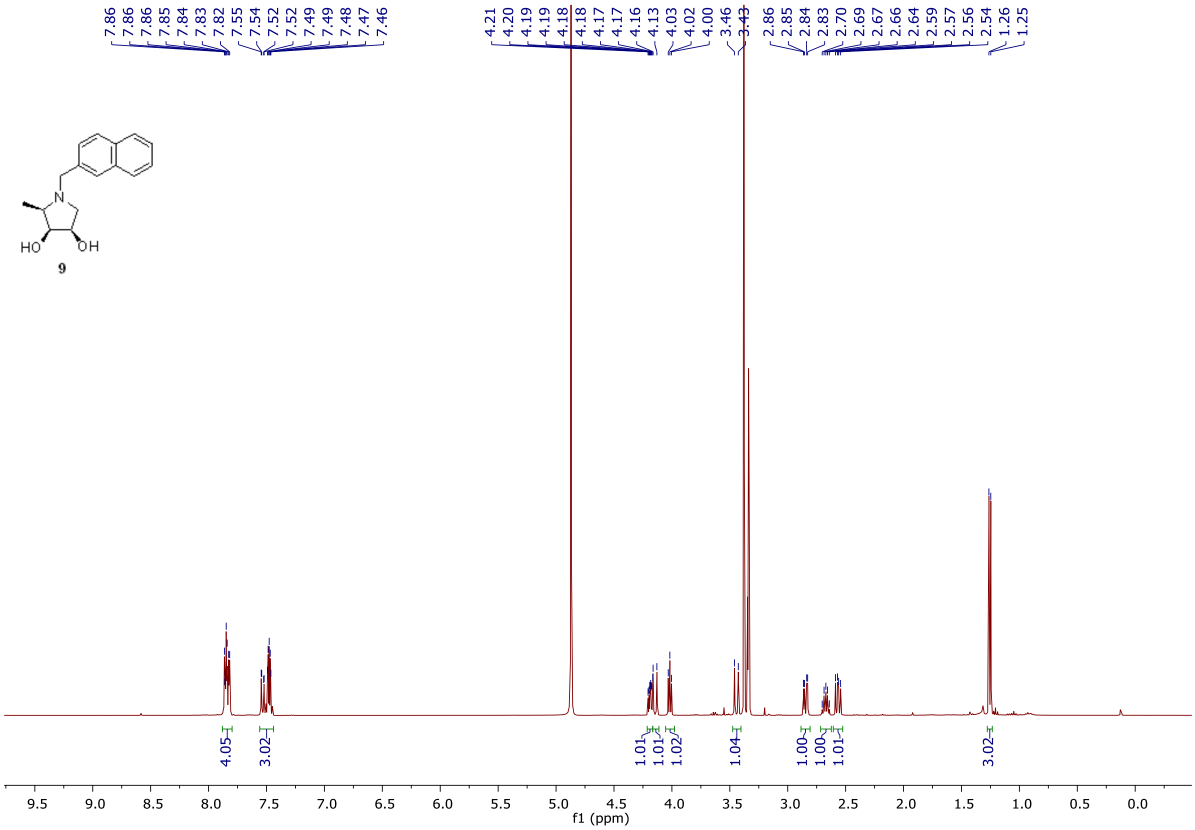
7.86
7.86
7.86
7.85
7.84
7.83
7.82
7.55
7.54
7.52
7.52
7.49
7.49
7.48
7.47
7.46
4.21
4.20
4.19
4.19
4.18
4.18
4.17
4.17
4.16
4.13
4.03
4.02
4.00
3.46
3.43
2.86
2.85
2.84
2.83
2.70
2.69
2.67
2.66
2.64
2.59
2.57
2.56
2.54
1.26
1.25
N
HO
OH
9
4.05
3.02
1.01
1.01
1.02
1.04
1.00
1.00
1.01
3.02
f1 (ppm)

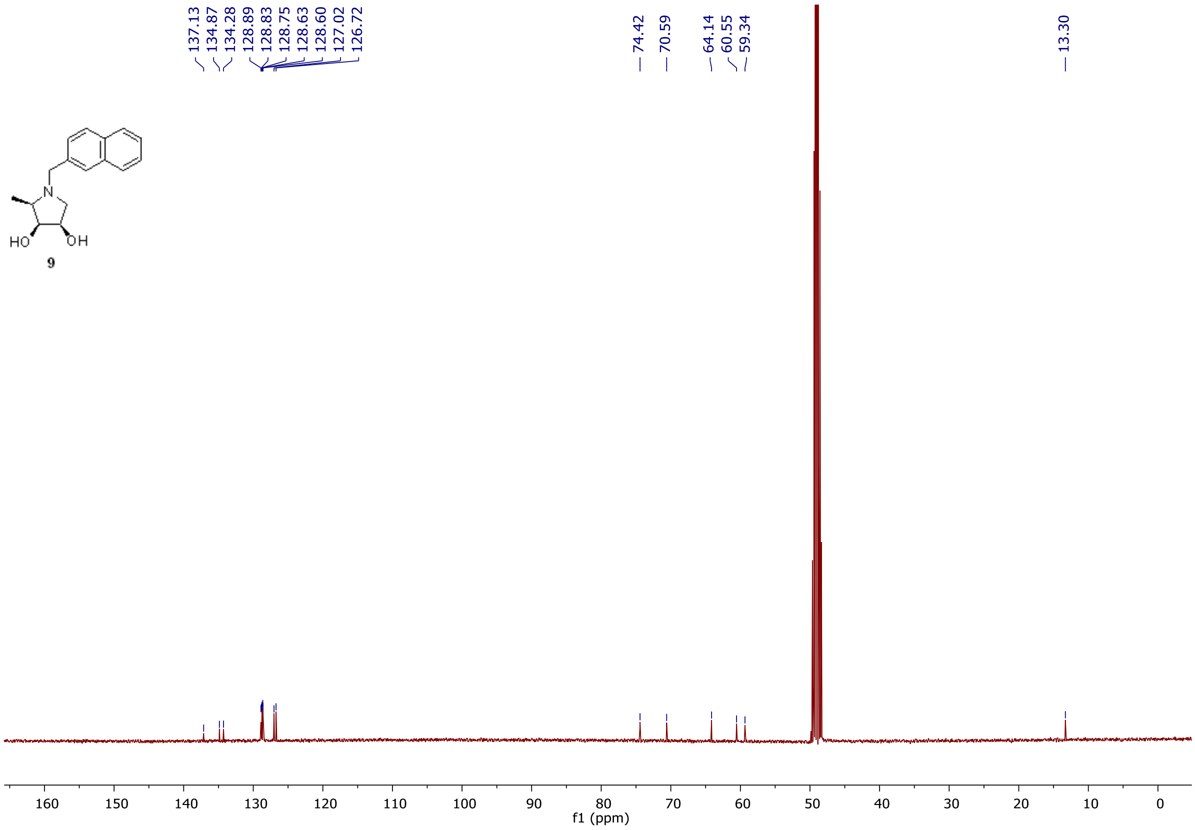
137.13
134.87
134.28
128.89
128.83
128.75
128.63
128.60
127.02
126.72
74.42
70.59
64.14
60.55
59.34
13.30
N
HO
OH
9
f1 (ppm)

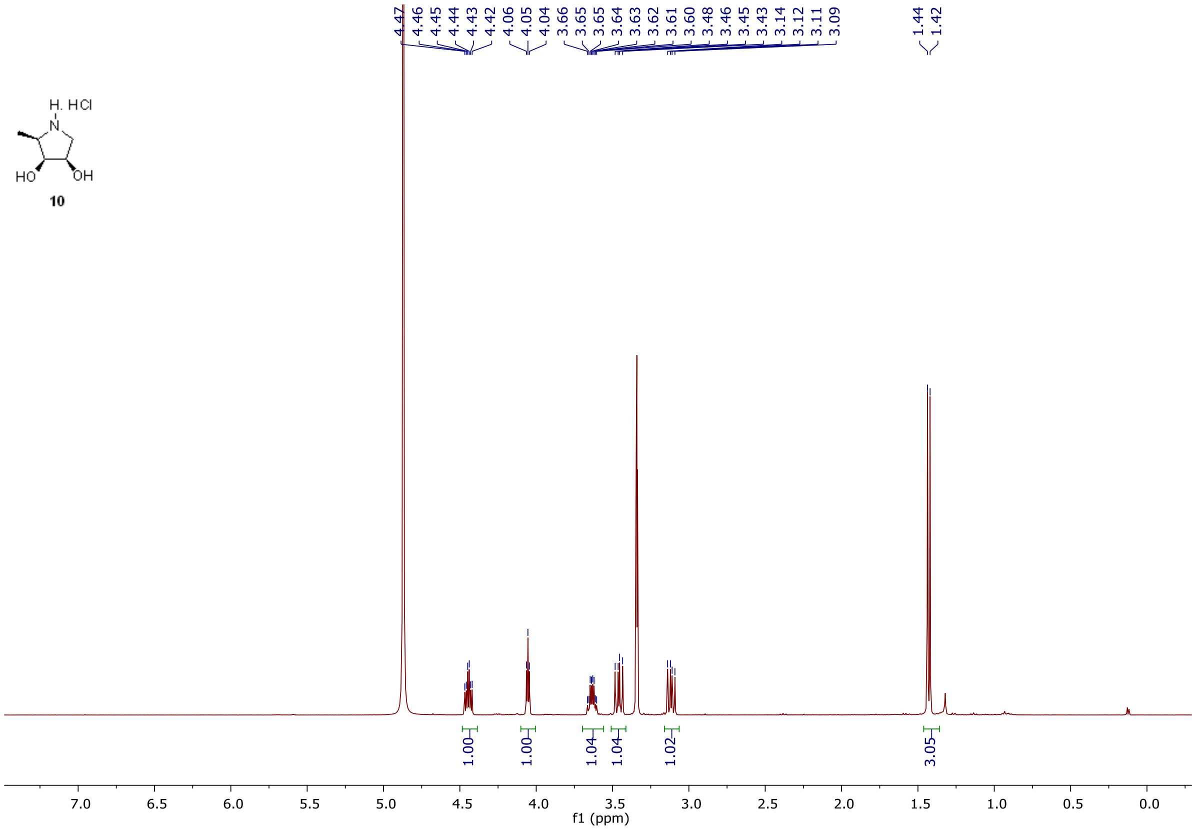
H. HCl
N
HO
OH
10
4.47
4.46
4.45
4.44
4.43
4.42
4.06
4.05
4.04
3.66
3.65
3.65
3.64
3.63
3.62
3.61
3.60
3.48
3.46
3.45
3.43
3.14
3.12
3.11
3.09
1.44
1.42
1.00
1.00
1.04
1.04
1.02
3.05
7.0
6.5
6.0
5.5
5.0
4.5
4.0
3.5
3.0
2.5
2.0
1.5
1.0
0.5
0.0
f1 (ppm)

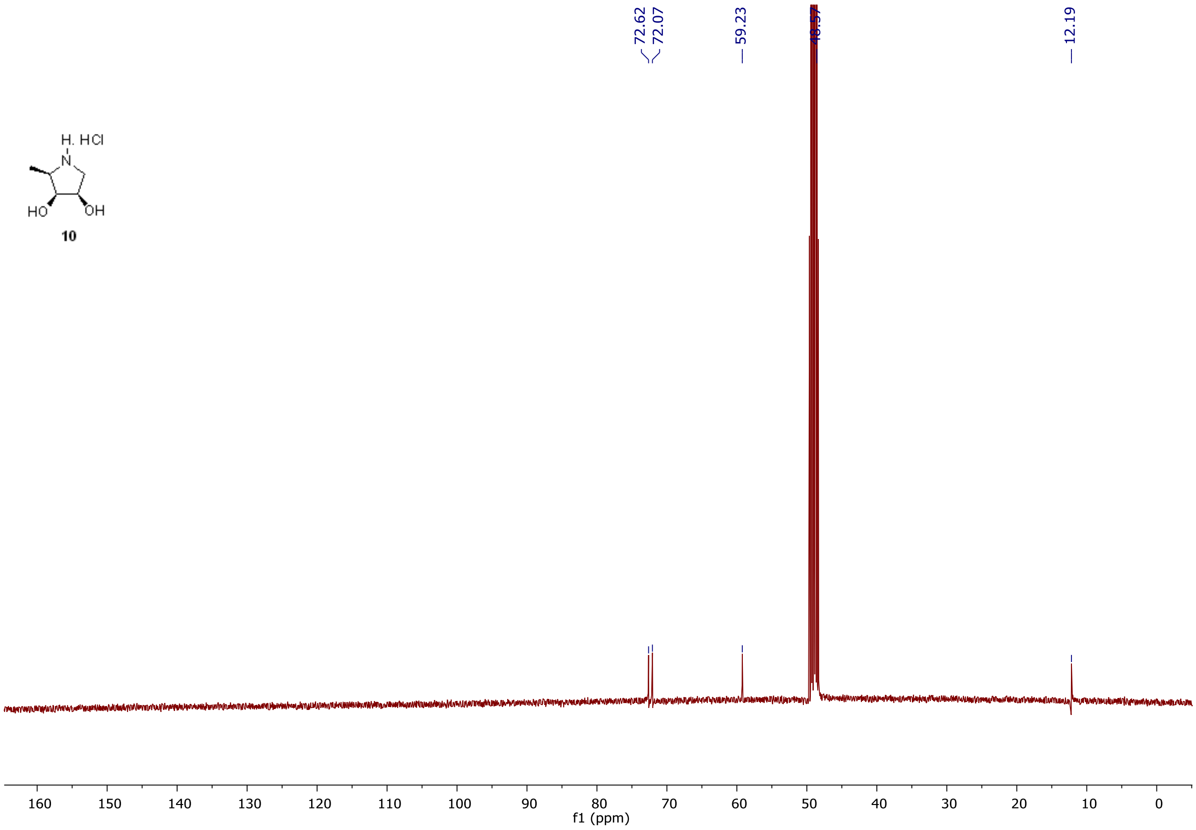
H. HCl
N
HO
OH
10
72.62
72.07
59.23
48.57
12.19
160
150
140
130
120
110
100
90
80
70
60
50
40
30
20
10
0
f1 (ppm)

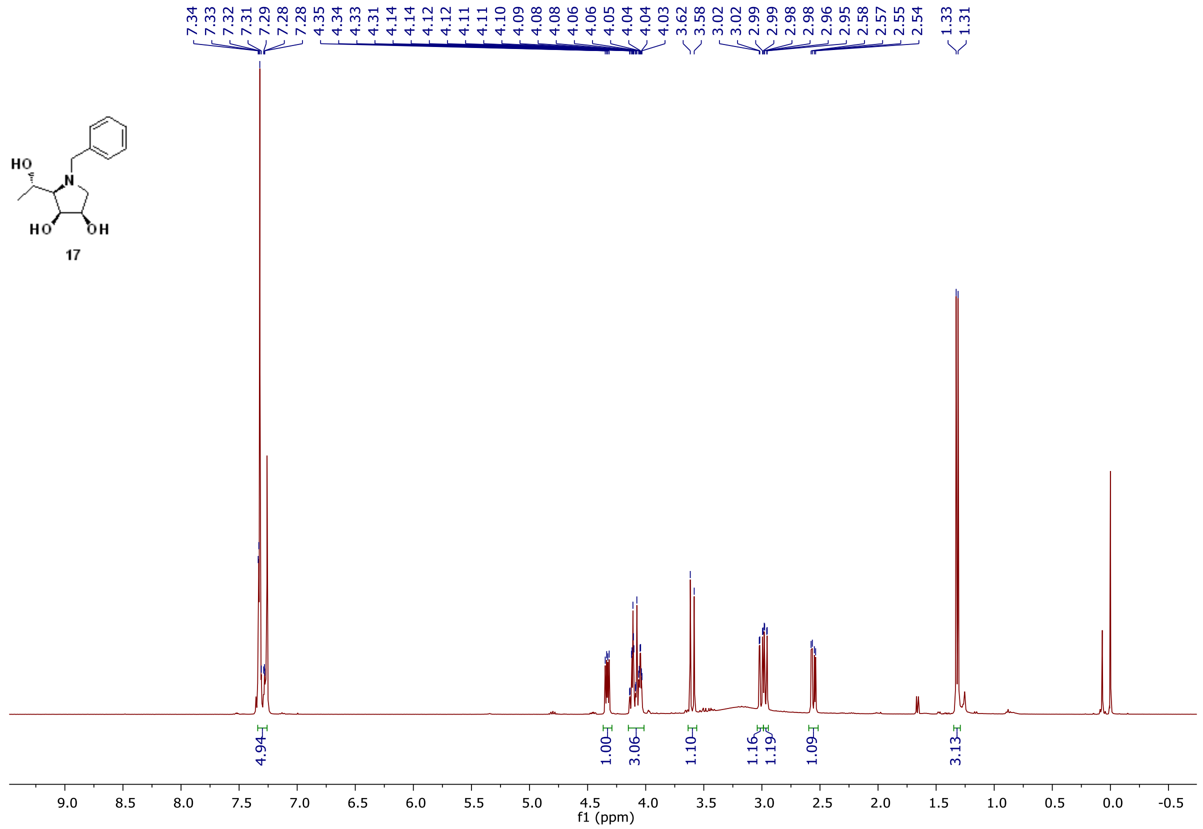
7.34 7.33 7.32 7.31 7.29 7.28 7.28 4.35 4.34 4.33 4.31 4.14 4.14 4.12 4.12 4.11 4.11 4.10 4.09 4.08 4.08 4.06 4.06 4.05 4.04 4.04 4.03 3.62 3.58 3.02 2.99 2.99 2.98 2.96 2.95 2.58 2.57 2.55 2.54 1.33 1.31
HO
N
HO
OH
17
4.94
1.00
3.06
1.10
1.16
1.19
1.09
3.13
9.0 8.5 8.0 7.5 7.0 6.5 6.0 5.5 5.0 4.5 4.0 3.5 3.0 2.5 2.0 1.5 1.0 0.5 0.0 -0.5
f1 (ppm)

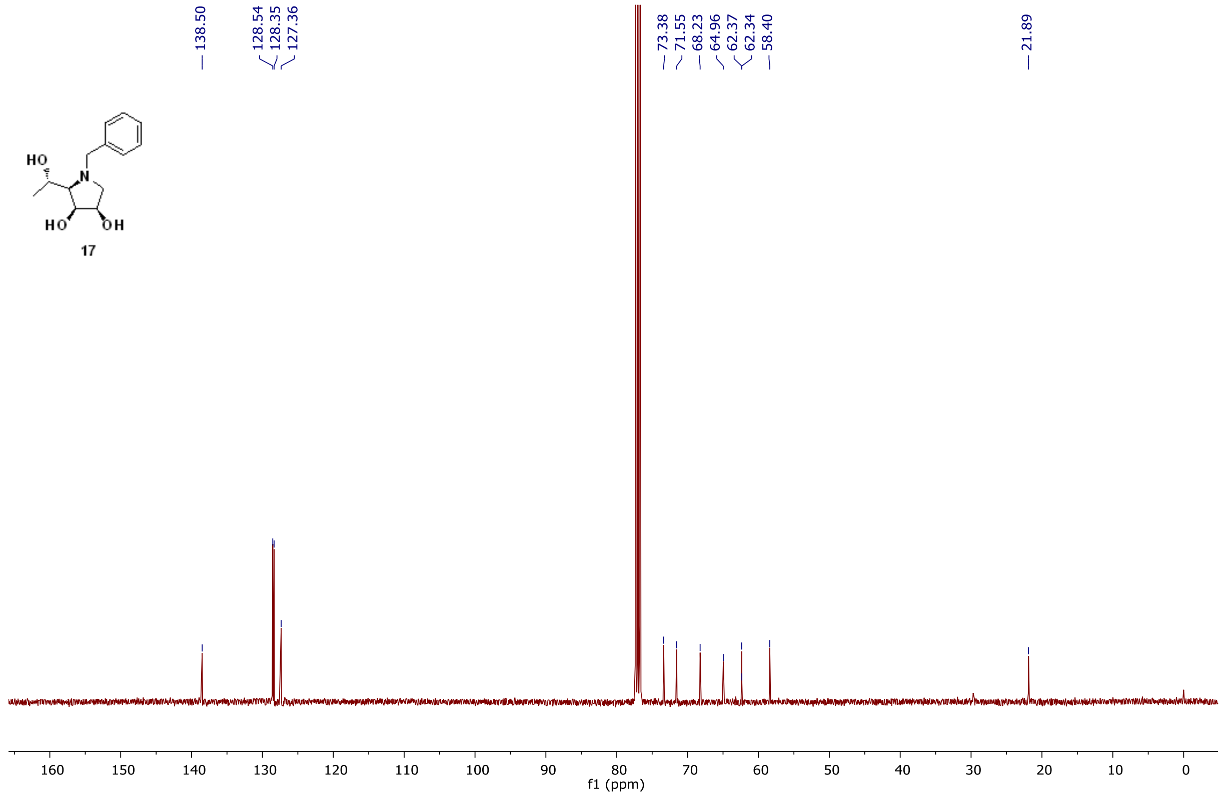
138.50
128.54 128.35 127.36
73.38 71.55 68.23 64.96 62.37 62.34 58.40
21.89
HO
N
HO
OH
17
160 150 140 130 120 110 100 90 80 70 60 50 40 30 20 10 0
f1 (ppm)

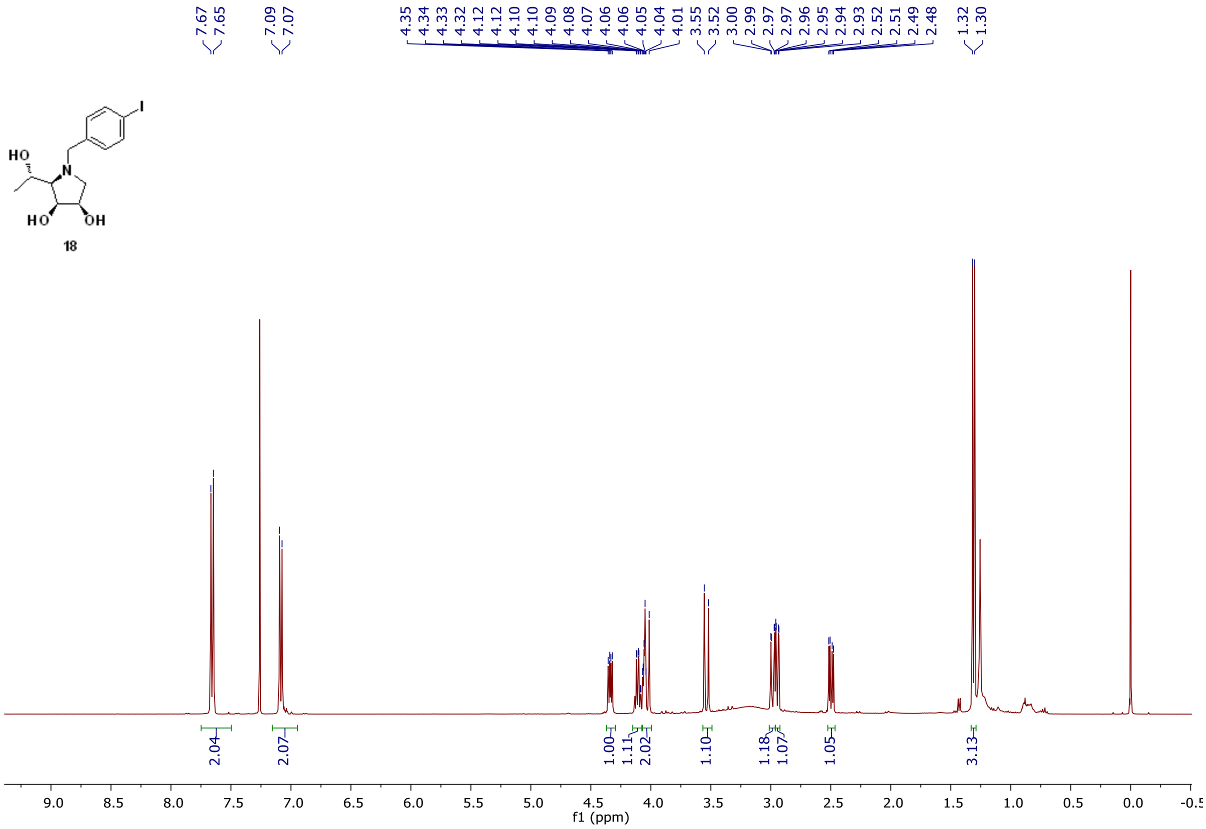
7.67
7.65
7.09
7.07
4.35
4.34
4.33
4.32
4.12
4.12
4.10
4.10
4.09
4.08
4.07
4.06
4.06
4.05
4.04
4.01
3.55
3.52
3.00
2.99
2.97
2.97
2.96
2.95
2.94
2.93
2.52
2.51
2.49
2.48
1.32
1.30
HO
N
I
HO
OH
18
2.04
2.07
1.00
1.11
2.02
1.10
1.18
1.07
1.05
3.13
f1 (ppm)

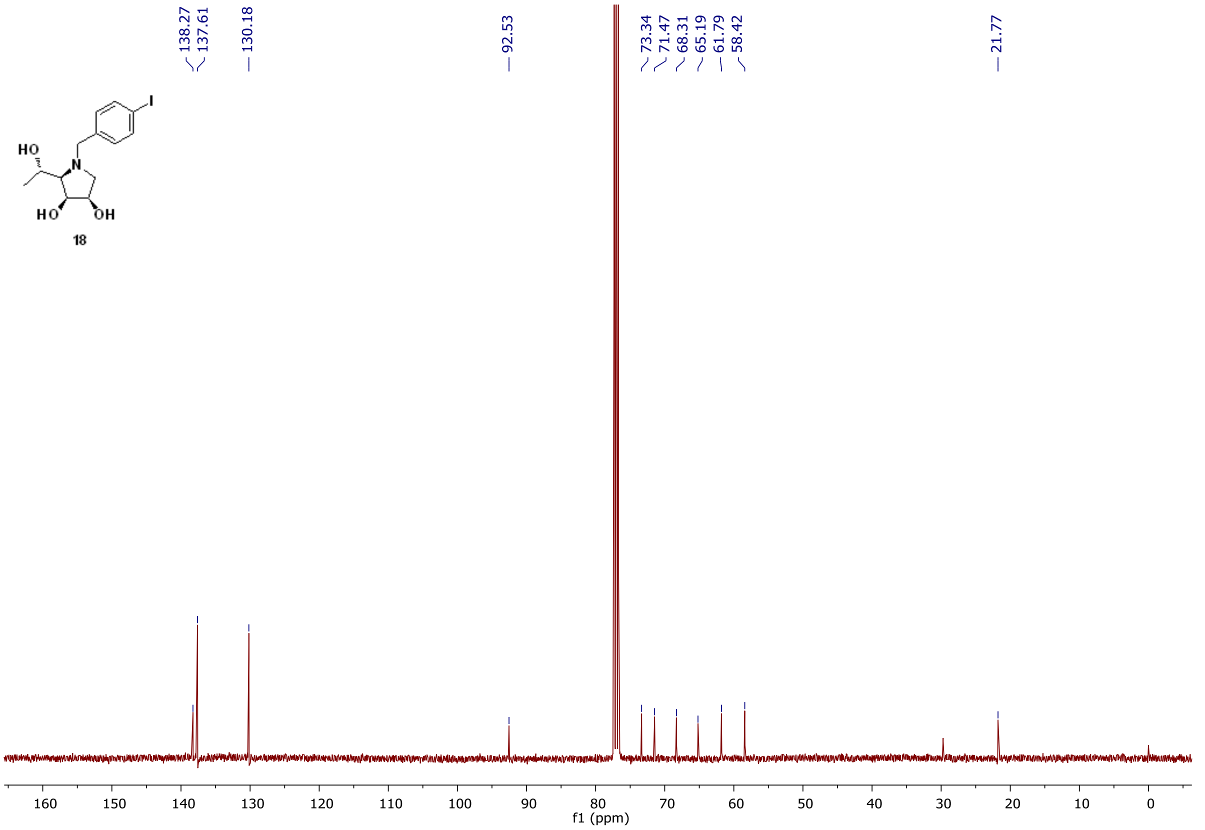
138.27
137.61
130.18
92.53
73.34
71.47
68.31
65.19
61.79
58.42
21.77
HO
N
I
HO
OH
18
f1 (ppm)

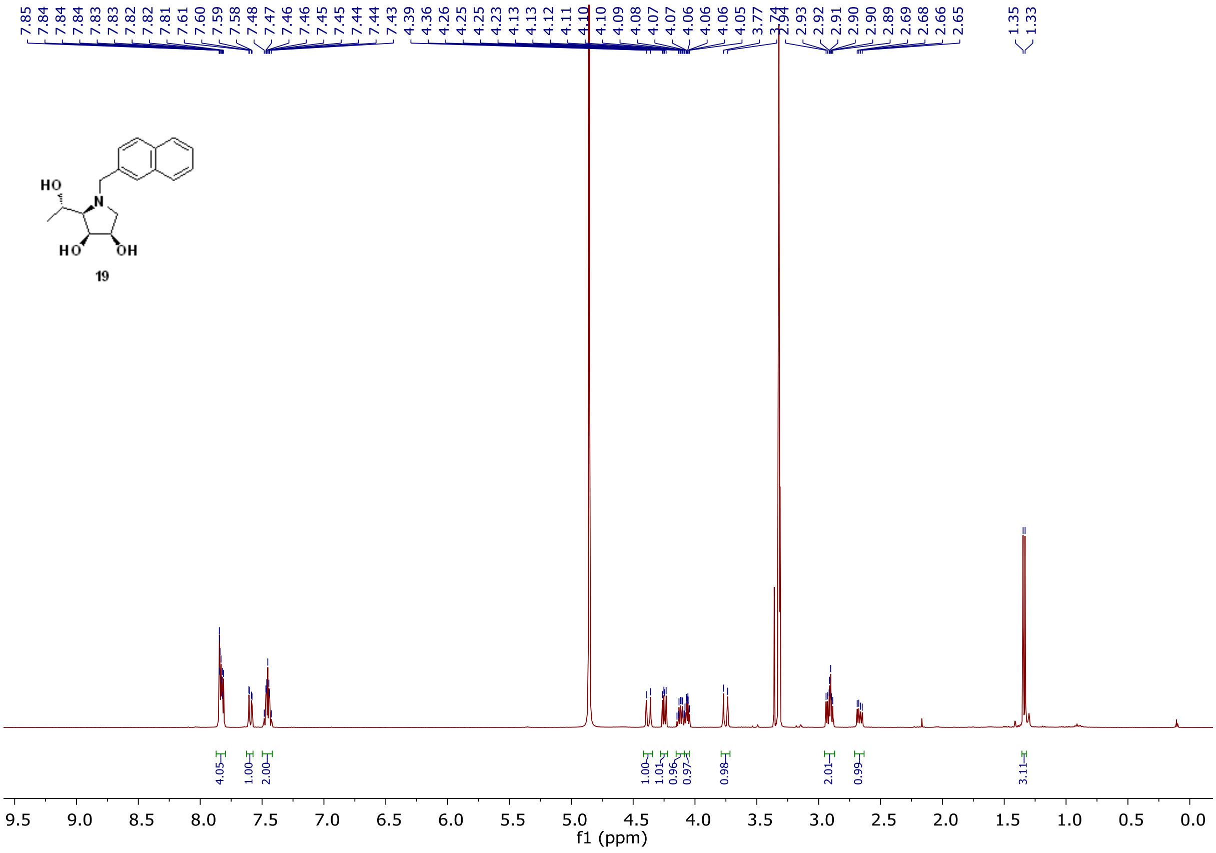
7.85 7.84 7.84 7.83 7.83 7.82 7.82 7.81 7.61 7.60 7.59 7.58 7.48 7.47 7.46 7.46 7.45 7.45 7.44 7.43 4.39 4.36 4.26 4.25 4.25 4.23 4.13 4.13 4.12 4.11 4.10 4.10 4.09 4.08 4.07 4.07 4.06 4.06 4.06 4.05 3.77 3.74 2.94 2.93 2.92 2.91 2.90 2.90 2.89 2.69 2.68 2.66 2.65 1.35 1.33
HO
N
HO
OH
19
4.05 1.00 2.00 1.00 1.01 0.96 0.97 0.98 2.01 0.99 3.11
9.5 9.0 8.5 8.0 7.5 7.0 6.5 6.0 5.5 5.0 4.5 4.0 3.5 3.0 2.5 2.0 1.5 1.0 0.5 0.0
f1 (ppm)

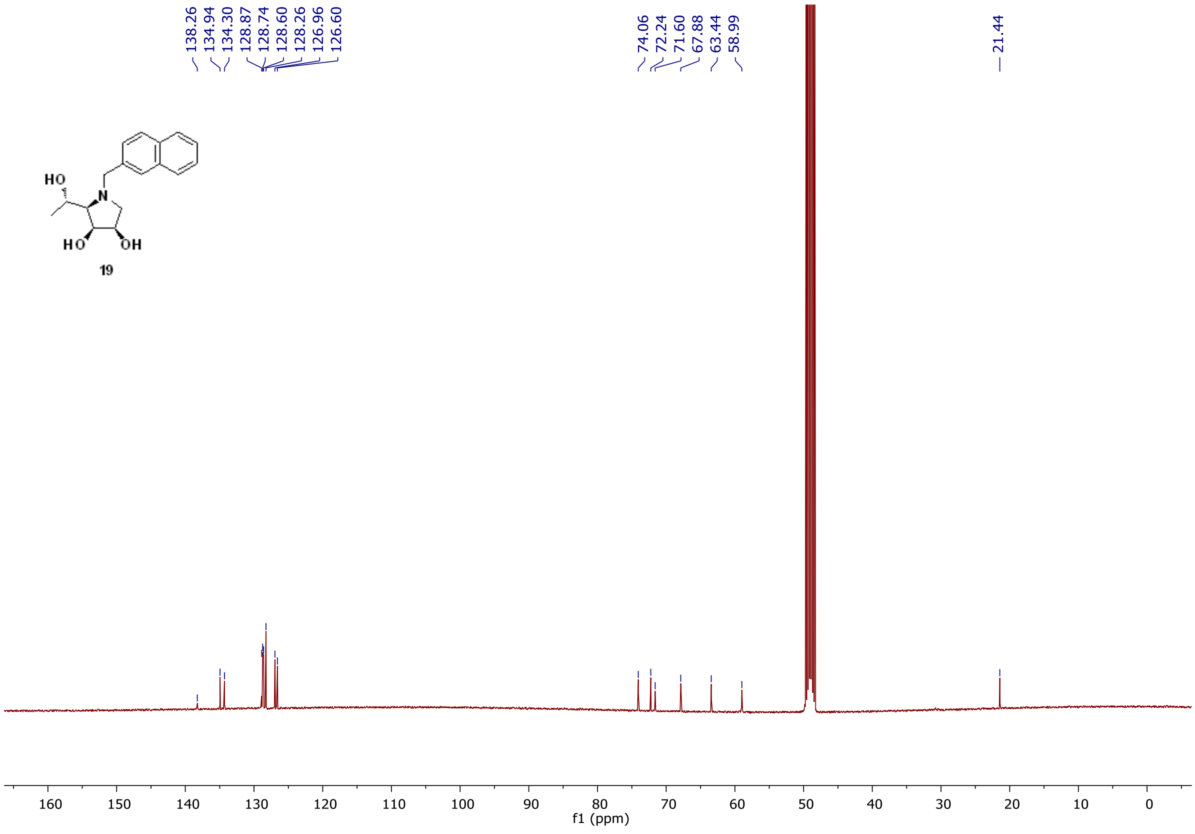
138.26 134.94 134.30 128.87 128.74 128.60 128.26 126.96 126.60
74.06 72.24 71.60 67.88 63.44 58.99
21.44
HO
N
HO
OH
19
160 150 140 130 120 110 100 90 80 70 60 50 40 30 20 10 0
f1 (ppm)

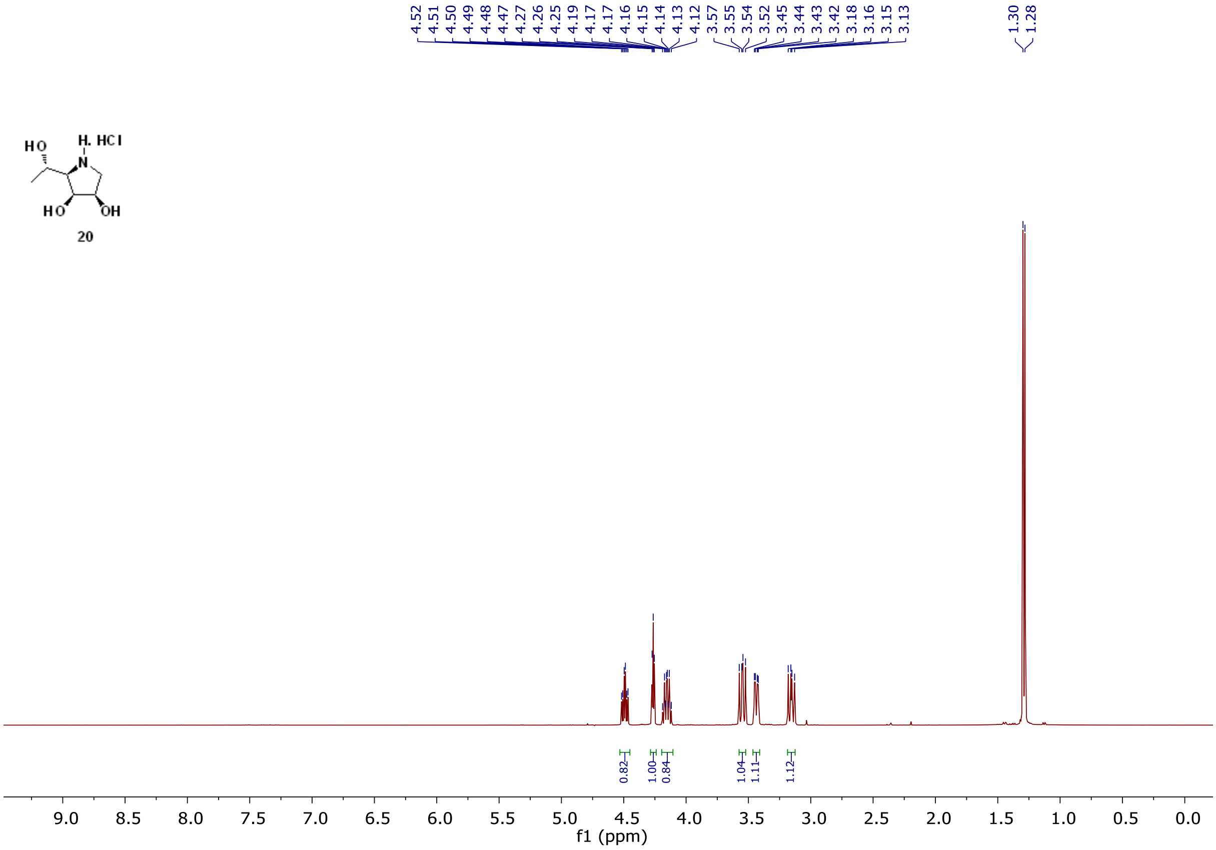
4.52
4.51
4.50
4.49
4.48
4.47
4.27
4.26
4.25
4.19
4.17
4.17
4.16
4.15
4.14
4.13
4.12
3.57
3.55
3.54
3.52
3.45
3.44
3.43
3.42
3.18
3.16
3.15
3.13
1.30
1.28
HO
H. HCl
N
HO
OH
20
0.82
1.00
0.84
1.04
1.11
1.12
9.0
8.5
8.0
7.5
7.0
6.5
6.0
5.5
5.0
4.5
4.0
3.5
3.0
2.5
2.0
1.5
1.0
0.5
0.0
f1 (ppm)

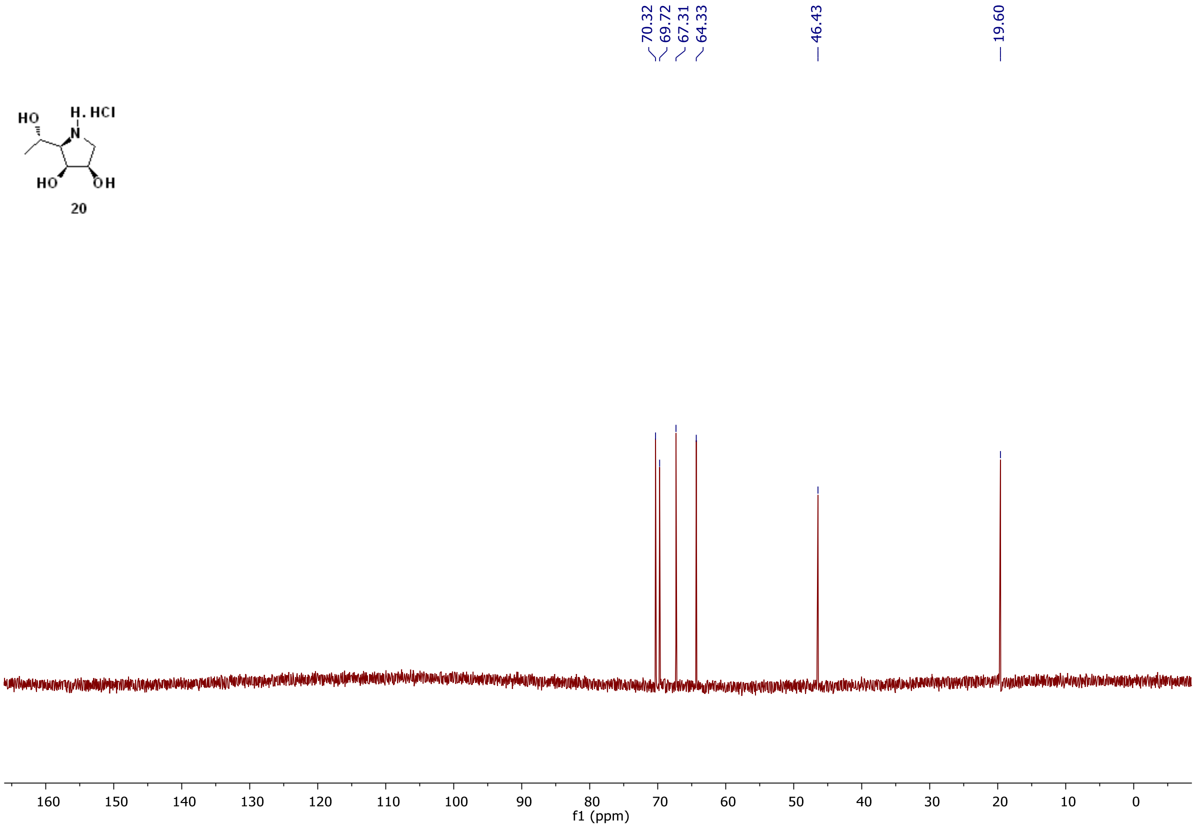
70.32
69.72
67.31
64.33
46.43
19.60
HO
H. HCl
N
HO
OH
20
160
150
140
130
120
110
100
90
80
70
60
50
40
30
20
10
0
f1 (ppm)

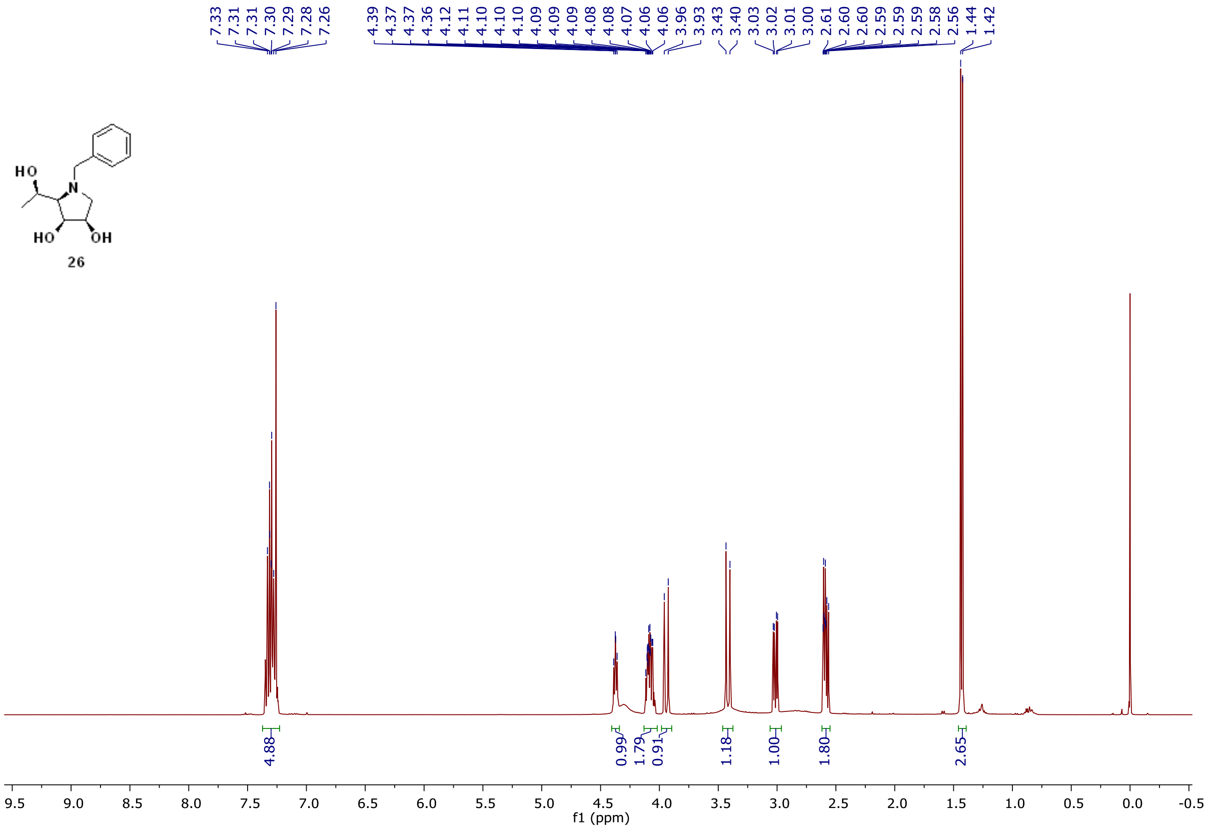
7.33
7.31
7.31
7.30
7.29
7.28
7.26
4.39
4.37
4.37
4.36
4.12
4.11
4.10
4.10
4.09
4.09
4.09
4.08
4.08
4.07
4.06
4.06
3.96
3.93
3.43
3.40
3.03
3.02
3.01
3.00
2.61
2.60
2.60
2.59
2.59
2.59
2.58
2.56
1.44
1.42
HO
N
HO
OH
26
4.88
0.99
1.79
0.91
1.18
1.00
1.80
2.65
f1 (ppm)

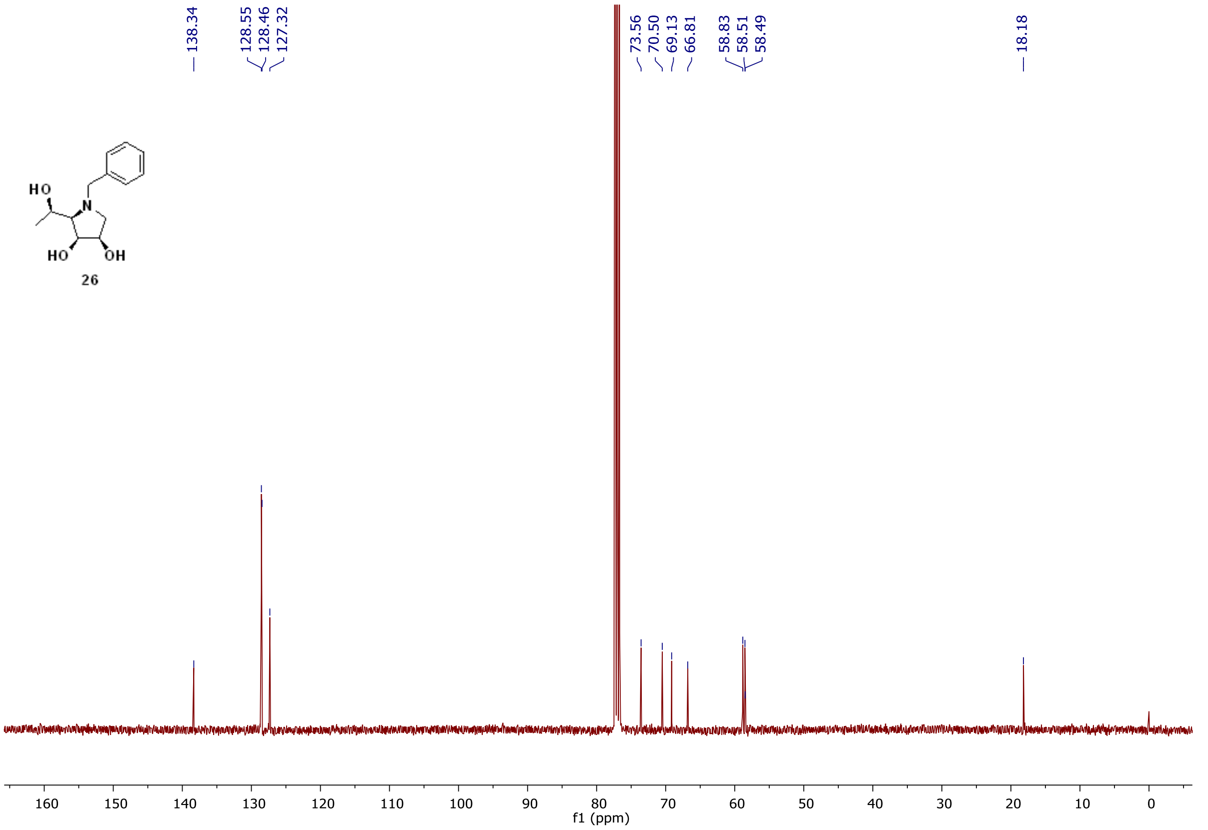
138.34
128.55
128.46
127.32
73.56
70.50
69.13
66.81
58.83
58.51
58.49
18.18
HO
N
HO
OH
26
f1 (ppm)

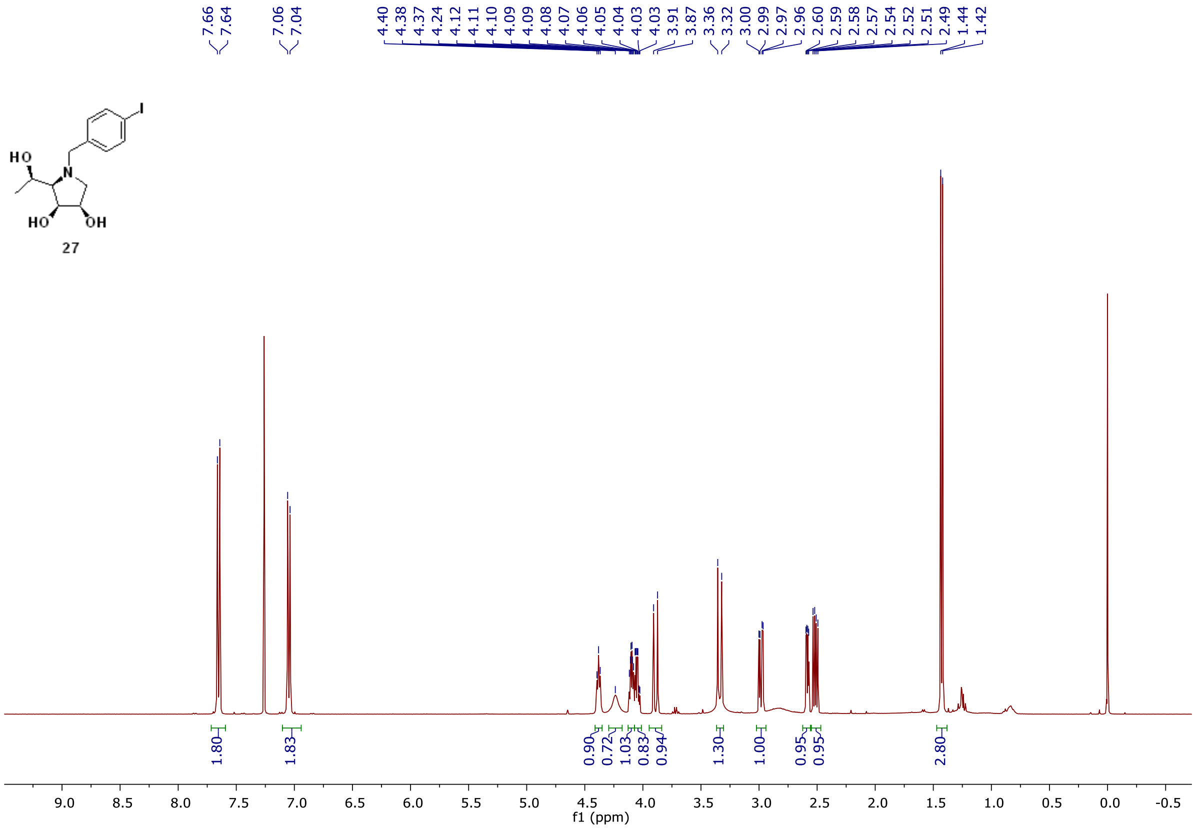
7.66
7.64
7.06
7.04
4.40
4.38
4.37
4.24
4.12
4.11
4.10
4.09
4.09
4.08
4.07
4.06
4.05
4.04
4.03
4.03
3.91
3.87
3.36
3.32
3.00
2.99
2.97
2.96
2.60
2.59
2.58
2.57
2.54
2.52
2.51
2.49
1.44
1.42
HO
N
I
HO
OH
27
1.80
1.83
0.90
0.72
1.03
0.83
0.94
1.30
1.00
0.95
0.95
2.80
9.0 8.5 8.0 7.5 7.0 6.5 6.0 5.5 5.0 4.5 4.0 3.5 3.0 2.5 2.0 1.5 1.0 0.5 0.0 -0.5
f1 (ppm)

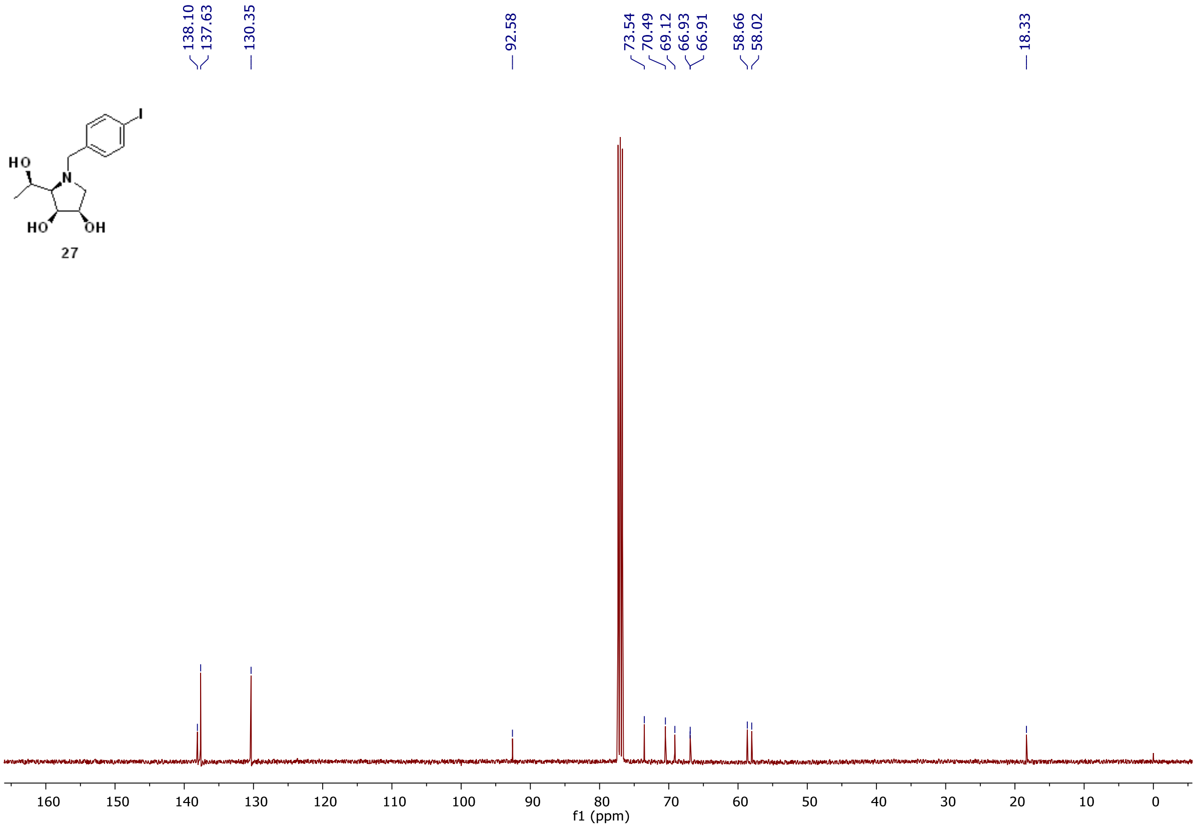
138.10
137.63
130.35
92.58
73.54
70.49
69.12
66.93
66.91
58.66
58.02
18.33
HO
N
I
HO
OH
27
160 150 140 130 120 110 100 90 80 70 60 50 40 30 20 10 0
f1 (ppm)

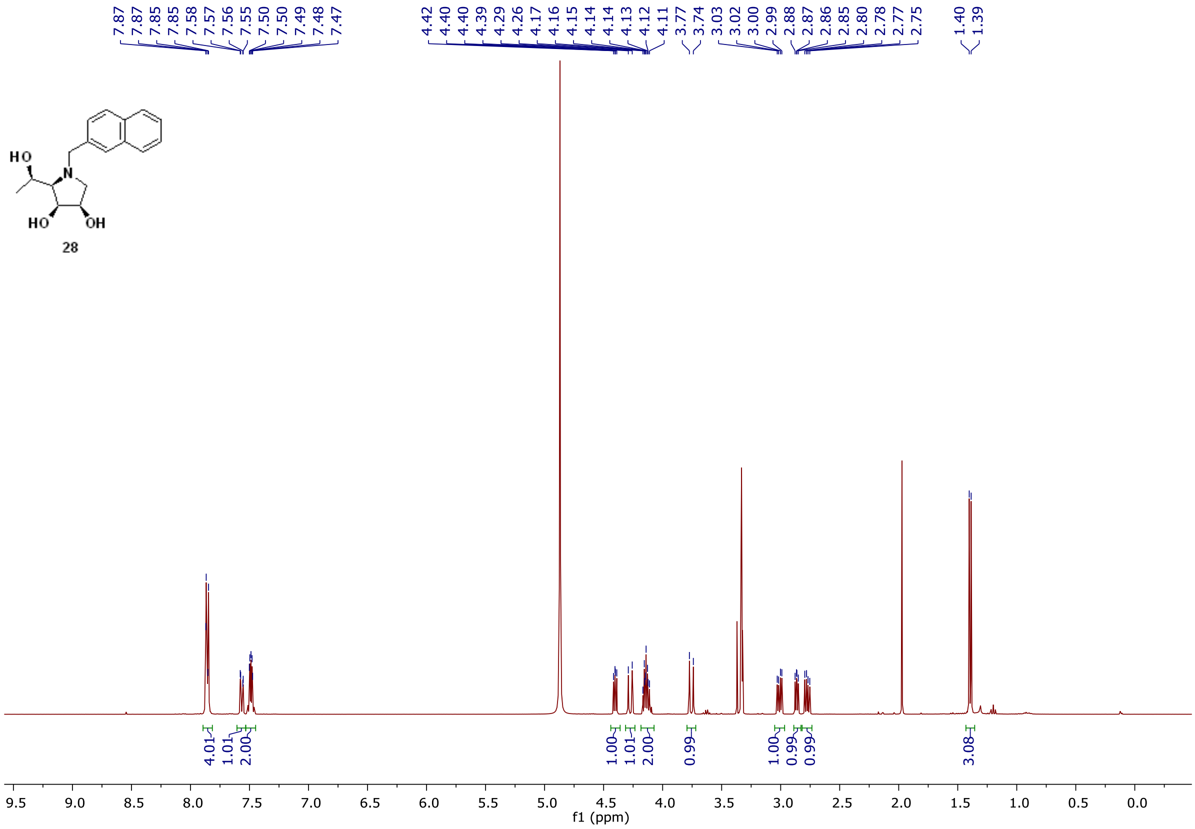
7.87
7.87
7.85
7.85
7.58
7.57
7.56
7.55
7.50
7.50
7.49
7.48
7.47
4.42
4.40
4.40
4.39
4.29
4.26
4.17
4.16
4.15
4.14
4.14
4.13
4.12
4.11
3.77
3.74
3.03
3.02
3.00
2.99
2.88
2.87
2.86
2.85
2.80
2.78
2.77
2.75
1.40
1.39
HO
N
HO
OH
28
4.01
1.01
2.00
1.00
1.01
2.00
0.99
1.00
0.99
0.99
3.08
f1 (ppm)

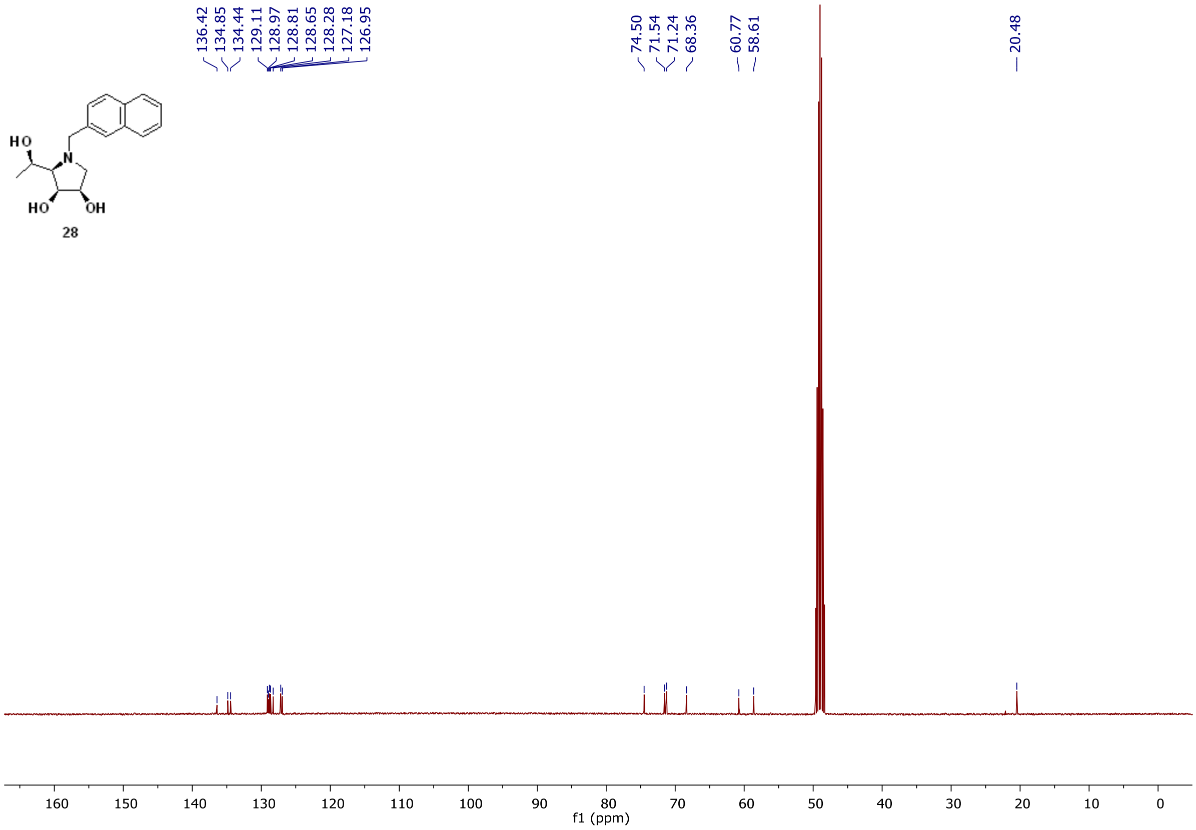
136.42
134.85
134.44
129.11
128.97
128.81
128.65
128.28
127.18
126.95
74.50
71.54
71.24
68.36
60.77
58.61
20.48
HO
N
HO
OH
28
f1 (ppm)

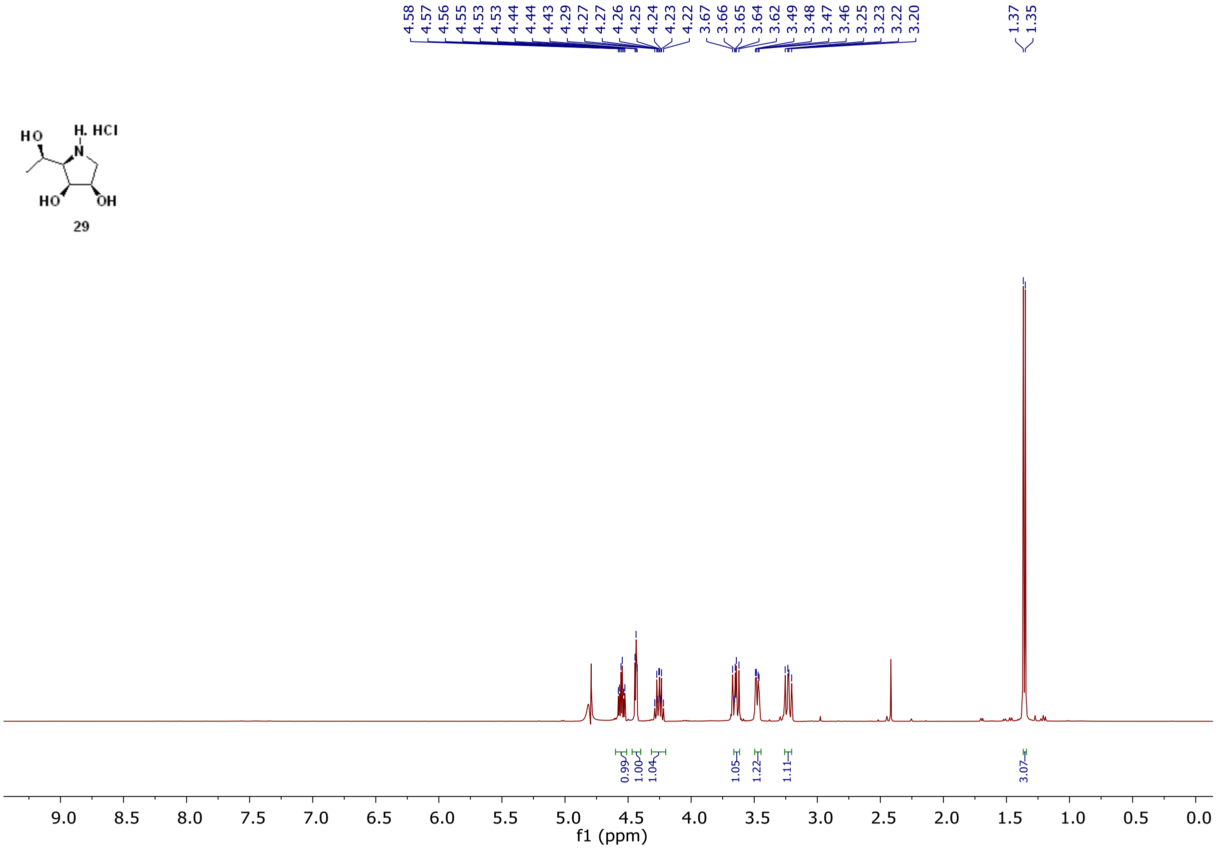
H. HCl
HO
HO
OH
29
4.58
4.57
4.56
4.55
4.53
4.53
4.44
4.44
4.43
4.29
4.27
4.27
4.26
4.25
4.24
4.23
4.22
3.67
3.66
3.65
3.64
3.62
3.49
3.48
3.47
3.46
3.25
3.23
3.22
3.20
1.37
1.35
0.99
1.00
1.04
1.05
1.22
1.11
3.07
9.0 8.5 8.0 7.5 7.0 6.5 6.0 5.5 5.0 4.5 4.0 3.5 3.0 2.5 2.0 1.5 1.0 0.5 0.0
f1 (ppm)

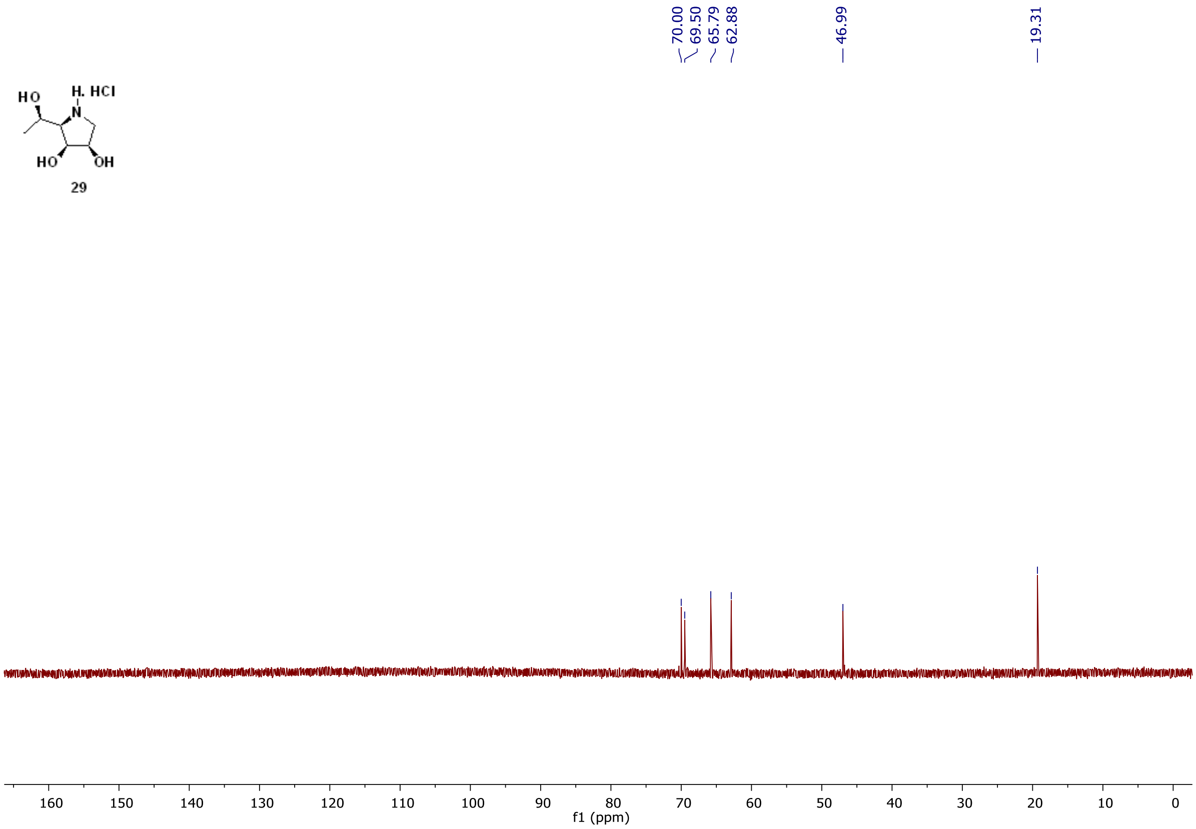
H. HCl
HO
HO
OH
29
70.00
69.50
65.79
62.88
46.99
19.31
160 150 140 130 120 110 100 90 80 70 60 50 40 30 20 10 0
f1 (ppm)
